# A bifunctional kinase–phosphatase module balances mitotic checkpoint strength and kinetochore–microtubule attachment stability

**DOI:** 10.15252/embj.2022112630

**Published:** 2023-09-15

**Authors:** Andrea Corno, Marilia H Cordeiro, Lindsey A Allan, Qian‐Wei Lim, Elena Harrington, Richard J Smith, Adrian T Saurin

**Affiliations:** ^1^ Cellular and Systems Medicine, School of Medicine University of Dundee Dundee UK

**Keywords:** error‐correction signalling, KNL1, mitotic checkpoint, PLK1, PP2A‐B56, Cell Cycle, Post-translational Modifications & Proteolysis

## Abstract

Two major mechanisms safeguard genome stability during mitosis: the mitotic checkpoint delays mitosis until all chromosomes have attached to microtubules, and the kinetochore–microtubule error‐correction pathway keeps this attachment process free from errors. We demonstrate here that the optimal strength and dynamics of these processes are set by a kinase–phosphatase pair (PLK1‐PP2A) that engage in negative feedback from adjacent phospho‐binding motifs on the BUB complex. Uncoupling this feedback to skew the balance towards PLK1 produces a strong checkpoint, hypostable microtubule attachments and mitotic delays. Conversely, skewing the balance towards PP2A causes a weak checkpoint, hyperstable microtubule attachments and chromosome segregation errors. These phenotypes are associated with altered BUB complex recruitment to KNL1‐MELT motifs, implicating PLK1‐PP2A in controlling auto‐amplification of MELT phosphorylation. In support, KNL1‐BUB disassembly becomes contingent on PLK1 inhibition when KNL1 is engineered to contain excess MELT motifs. This elevates BUB‐PLK1/PP2A complex levels on metaphase kinetochores, stabilises kinetochore–microtubule attachments, induces chromosome segregation defects and prevents KNL1‐BUB disassembly at anaphase. Together, these data demonstrate how a bifunctional PLK1/PP2A module has evolved together with the MELT motifs to optimise BUB complex dynamics and ensure accurate chromosome segregation.

## Graphical abstract

PLK1 and PP2A‐B56 are engaged in an intramolecular negative feedback loop on BUBR1, with their levels being set by the number of MELT motifs in KNL1.

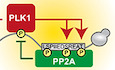

## Introduction

Two key mitotic surveillance pathways have evolved to preserve genome stability by safeguarding the chromosome segregation process. The mitotic checkpoint (also known as the spindle assembly checkpoint or SAC) prevents mitotic exit until all chromosomes have attached to microtubules via the kinetochore (Lara‐Gonzalez *et al*, 2021b). The kinetochore–microtubule (KT‐MT) error‐correction pathway continually monitors and corrects these attachments to ensure they remain free from errors (Lampson & Grishchuk, 2017). Both of these processes are regulated by dynamic phosphorylation events at the kinetochore (Vallardi *et al*, 2017; Saurin, 2018).

The SAC is activated by the phosphorylation of MELT repeats on the kinetochore signalling scaffold KNL1 (London *et al*, 2012; Shepperd *et al*, 2012; Yamagishi *et al*, 2012), which localises to the outer kinetochore as part of the KNL1/MIS12/NDC80 (KMN) network. The phosphorylated MELT repeats recruit the BUB complex to kinetochores, which act as a platform for the assembly of an inhibitory complex that can block mitotic exit: termed the mitotic checkpoint complex or MCC (Lara‐Gonzalez *et al*, 2021b). Phosphorylation is also needed on multiple MCC components to drive complex assembly (Faesen *et al*, 2017; Ji *et al*, 2017, 2018; Qian *et al*, 2017; Zhang *et al*, 2017; Piano *et al*, 2021; Lara‐Gonzalez *et al*, 2021a; Fischer *et al*, 2022; Fischer, 2023), and when MCC is generated, it is released from kinetochores to inhibit the anaphase‐promoting complex/cyclosome (APC/C); a large E3 ubiquitin ligase that otherwise induces chromosome segregation and mitotic exit by degrading securin and cyclin B (Lara‐Gonzalez *et al*, 2021b). The key phosphorylation events that drive MCC formation at kinetochores must be dynamic (i.e. responsive to change) because as soon as microtubule attaches, MCC assembly must be rapidly shut down on KNL1. The kinetochore phosphatases PP2A‐B56 and PP1, which bind to the BUB complex and KNL1, respectively, are crucial for dephosphorylating key sites to allow this rapid SAC silencing (Meadows *et al*, 2011; Rosenberg *et al*, 2011; Espeut *et al*, 2012; London *et al*, 2012; Espert *et al*, 2014; Nijenhuis *et al*, 2014; Cordeiro *et al*, 2020). This ultimately helps to ensure that the SAC signal is switched off and the APC/C is activated within seconds after the last kinetochore attaches to microtubules (Collin *et al*, 2013; Dick & Gerlich, 2013).

The error‐correction process also critically relies on rapidly switching phospho‐sites, but in this case, the phosphorylation events are inhibitory to the microtubule attachment process (Saurin, 2018). That is because they are located on the microtubule attachment interface, including on multiple residues in the N‐terminal tail of NDC80, where they electrostatically interfere with microtubule binding (Wimbish & DeLuca, 2020). The purpose of these phosphorylations is to help correct attachment errors, and they achieve this by responding differently depending on the type of microtubule attachments that form. If the attachments are the correct amphitelic configuration—that is, each sister kinetochore is attached to opposite spindle poles—then this exerts pulling force across the kinetochores. This “tension” is sensed, by a still poorly understood pathway, and the inhibitory phosphorylation sites on the attachment interface are dephosphorylated to stabilise microtubule binding. If, however, tension is not generated because the attachments are incorrect, then phospho‐signals persist and those faulty attachments are destabilised, thus freeing the kinetochore to try again to form the correct amphitelic configuration (Lampson & Grishchuk, 2017; McVey *et al*, 2021). Responsive phosphorylation sites are crucial here too because if they cannot be dephosphorylated rapidly following tension, then even the correct microtubule attachments are destabilised and mitotic progression is delayed or prevented. That is essentially the phenotype observed when PP2A‐B56 is removed from the BUB complex (Suijkerbuijk *et al*, 2012; Kruse *et al*, 2013; Xu *et al*, 2013), demonstrating that this phosphatase complex plays a crucial role in error correction, as well as in SAC silencing.

PP2A‐B56 regulates both SAC silencing and KT‐MT attachments by associating with the MCC component BUBR1 (Suijkerbuijk *et al*, 2012; Kruse *et al*, 2013; Xu *et al*, 2013), which localises to phosphorylated MELT repeats on KNL1 by binding to BUB1 and BUB3 (this BUB1‐BUB3/BUB3‐BUBR1 heterodimer is hereafter referred to as the BUB complex) (London *et al*, 2012; Shepperd *et al*, 2012; Yamagishi *et al*, 2012; Primorac *et al*, 2013; Vleugel *et al*, 2013; Zhang *et al*, 2014; Overlack *et al*, 2015). PP2A‐B56 binds to a domain in BUBR1 known as the KARD (amino acids 664–681), which contains a phospho‐dependent PP2A‐B56‐binding motif (Suijkerbuijk *et al*, 2012; Kruse *et al*, 2013; Hertz *et al*, 2016; Wang *et al*, 2016a, 2016b). We demonstrated recently that PP2A‐B56 controls SAC silencing principally by removing PLK1 from adjacent phospho‐binding motifs on BUBR1 (pThr620) and BUB1 (pThr609) (Cordeiro *et al*, 2020). From these residues, PLK1 can promote SAC signalling by phosphorylating the KNL1‐MELT repeats and enhancing BUB complex recruitment to KNL1. In addition to phosphorylating the MELT motifs, PLK1 also phosphorylates Ser676 and Thr680 within the KARD of BUBR1 to enhance PP2A‐B56 recruitment to kinetochores (Elowe *et al*, 2007; Suijkerbuijk *et al*, 2012; Kruse *et al*, 2013; Wang *et al*, 2016a, 2016b), thereby indirectly stabilising KT‐MT attachments. Together, this implies that PLK1 and PP2A co‐regulate two key mitosis processes from their adjacent phospho‐binding sites on BUBR1, which have co‐evolved throughout metazoa (Cordeiro *et al*, 2020). We set out to characterise this bifunctional kinase–phosphatase module and examine its role in regulating chromosome segregation.

## Results

### PLK1 and PP2A engage in an intramolecular negative feedback loop on BUBR1

**Figure 1:**
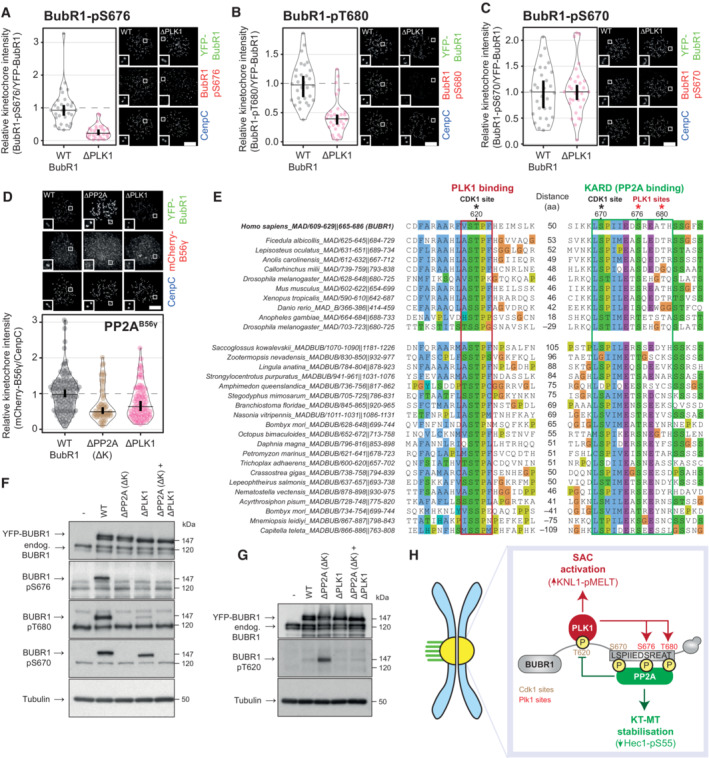
PLK1 and PP2A are engaged in an intramolecular negative feedback loop on BUBR1 A–DEffects of preventing PLK1 binding to BUBR1 (BUBR1‐T620A referred to as BUBR1^ΔPLK1^) on levels of BUBR1‐pS676 (A), BUBR1‐pT680 (B), BUBR1‐pS670 (C) and mCherry‐B56γ (D) at unattached kinetochores, in nocodazole‐arrested HeLa FRT cells expressing indicated YFP‐tagged BUBR1 constructs. Kinetochore intensities from 30 to 80 cells, 3–5 experiments. Kinetochore intensities are normalised to BUBR1 WT control. Violin plots show the distributions of kinetochore intensities between cells. For each violin plot, each dot represents an individual cell, the horizontal line represents the median and the vertical one the 95% CI of the median, which can be used for statistical comparison of different conditions (see Materials and Methods). Representative example immunofluorescence images of the kinetochore quantifications are shown for each BUBR1 phospho‐site in (A–D). The insets show magnifications of the outlined regions. Scale bars: 5 μm. Inset size: 1.5 μm.EAlignment of PLK1‐ and PP2A‐binding region on all MADBUB homologues in metazoa that contain a predicted PP2A‐binding motif. The number of residues between PLK1 and PP2A binding is reported between the two alignments.F, GMitotic HeLa FRT cells expressing indicated exogenous BUBR1 constructs were harvested and lysed. Lysates were then blotted with indicated antibodies.HSchematic illustrating how PLK1 and PP2A regulate each other's binding to BUBR1.

**Figure EV1:**
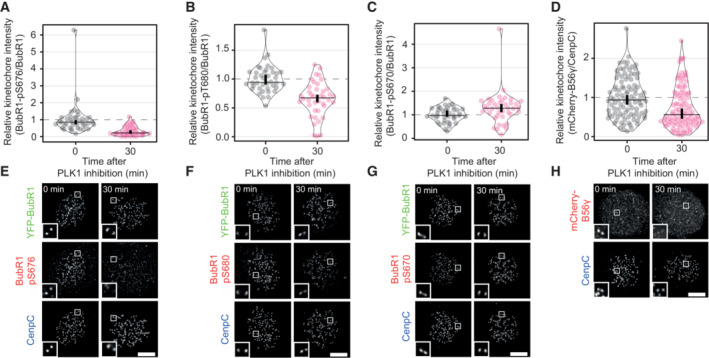
(Related to Fig 1). Effect of PLK1 inhibition on PP2A‐B56 recruitment sites and kinetochore localisation A–DEffects of PLK1 inhibition on levels of BUBR1‐pS676 (A), BUBR1‐pT680 (B), BUBR1‐pS670 (C) and mCherry‐B56γ (D) at unattached kinetochores, in nocodazole‐arrested HeLa FRT cells untreated or treated with the PLK1 inhibitor BI‐2536 (100 nM). Kinetochore intensities from 40 to 100 cells, 4–5 experiments. Kinetochore intensities are normalised to the time point 0′. Violin plots show the distributions of kinetochore intensities. For each violin plot, each dot represents an individual cell, the horizontal line represents the median and the vertical one the 95% CI of the median, which can be used for statistical comparison of different conditions (see Materials and Methods).E–HExample immunofluorescence images of the kinetochore quantifications shown in (A–D). The insets show magnifications of the outlined regions. Scale bars: 5 μm. Inset size: 1.5 μm.

To examine if PLK1 phosphorylates the KARD from its phospho‐Thr620 binding site on BUBR1, we analysed KARD phosphorylation in BUBR1^WT^ and BUBR1^T620A^ (hereafter referred to as BUBR1^ΔPLK1^) cells. Note, all mutant experiments were performed after knockdown and replacement of the endogenous gene, unless stated otherwise. Figure 1A and B show that S676 and T680 phosphorylation is reduced in BUBR1^ΔPLK1^ cells, demonstrating that local PLK1 recruitment is important for KARD phosphorylation. In contrast, phosphorylation of S670—a CDK1 site that also enhances PP2A‐B56 binding (Suijkerbuijk *et al*, 2012; Kruse *et al*, 2013; Wang *et al*, 2016a, 2016b)—is unaffected by mutation of the PLK1‐binding site (Fig 1C). The PLK1 phosphorylation sites in the KARD are important for PP2A‐B56 binding because BUBR1^ΔPLK1^ reduces B56γ at kinetochores (note B56γ/δ are the B56 isoforms that localise to the outer kinetochore: Vallardi *et al*, 2019) to a similar extent as deletion of the KARD domain (hereafter referred to a BUBR1^ΔPP2A(ΔK)^—Fig 1D). Catalytic activity of PLK1 is crucial for these effects because a 30 min incubation with the PLK1 inhibitor, BI‐2536 (Lenart *et al*, 2007), reduces pS676, pS680 and PP2A‐B56 levels, similarly to BUBR1^ΔPLK1^ mutation (Fig EV1A–H). A crucial role for PLK1‐mediated phosphorylation of the KARD is reinforced by the fact that the PLK1‐regulated S676 is completely conserved in the KARD of BUBR1, and in the ancestral MADBUB homologue, throughout metazoa (as either a Ser or Thr residue; Fig 1E) (Cordeiro *et al*, 2020). Furthermore, the phospho‐regulated PLK1‐binding motif is also fully conserved and almost always immediately adjacent to the KARD in BUBR1, or MADBUB homologues, and is often positioned around 50 amino acids prior to the KARD. Considering that the distance between these two binding domains was tightly conserved, we hypothesised that cross‐regulation between PLK1 and PP2A occurs intramolecularly.

To test this, we examined cross‐regulation between endogenous BUBR1 and BUBR1 mutants that were unable to bind to either PLK1 (BUBR1^ΔPLK1^) or PP2A (BUBR1^ΔPP2A(ΔK)^) (i.e. by expressing mutants without knocking down the endogenous BUBR1). Figure 1F demonstrates that phosphorylation of S676 and T680 is reduced on YFP‐tagged BUBR1^ΔPLK1^, but these sites remain largely unaltered on the endogenous BUBR1 protein, which is present in the same cells at similar levels. Similarly, T620 phosphorylation is only increased on YFP‐BUBR1^ΔPP2A(ΔK)^, and not on the endogenous BUBR1^WT^ protein that is also present in the same cells (Fig 1G). Therefore, PLK1 and PP2A‐B56 are engaged in an intramolecular negative feedback loop on BUBR1, with PLK1 enhancing PP2A and PP2A decreasing PLK1 (Fig 1H). Two functions of negative feedback loops are to achieve homeostasis and limit signalling output (Brandman & Meyer, 2008). PLK1 amplifies SAC signalling by phosphorylating MELT repeats (Espeut *et al*, 2015; von Schubert *et al*, 2015; Ikeda & Tanaka, 2017; Cordeiro *et al*, 2020), whereas PP2A‐B56 stabilises KT‐MT attachments by antagonising Aurora B to promote NDC80 dephosphorylation (Foley *et al*, 2011; Suijkerbuijk *et al*, 2012; Kruse *et al*, 2013; Xu *et al*, 2013; Smith *et al*, 2019) (Fig 1H). Excessive activity of either PLK1 or PP2A could be detrimental because it could lead to a hyperactive SAC that cannot switch off or hyperstable microtubules that cannot be corrected. We, therefore, hypothesised that negative feedback between PLK1 and PP2A recruitment was needed to balance their recruitment to BUBR1 and optimise SAC strength and KT‐MT attachment stability.

### PLK1 and PP2A co‐recruitment to BUBR1 is needed to balance SAC strength and KT‐MT attachment stability

**Figure 2:**
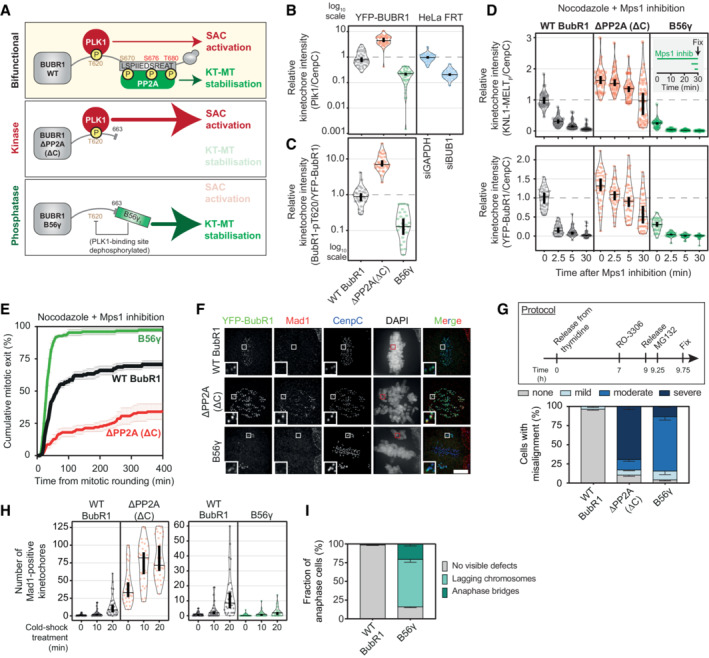
Locking the PLK1‐ or PP2A‐bound states on BUBR1 alters the strength of the SAC and KT‐MT attachments ASchematic illustrating PLK1 and PP2A bound to WT BUBR1 (Bifunctional, on top) and the BUBR1 mutants used to lock PLK1 (Kinase, mid panel) or PP2A (Phosphatase, bottom panel).B, CEffect of locking PLK1 or PP2A on levels of PLK1 (B) and BUBR1‐pT620 (C) at unattached kinetochores, in nocodazole‐arrested HeLa FRT cells expressing the indicated BUBR1 mutants or treated with control or BUB1 siRNAs. Kinetochore intensities from 30 to 40 cells, 3–4 experiments.D, EEffects of locking PLK1 or PP2A on KNL1‐MELT dephosphorylation and YFP‐BUBR1 levels at unattached kinetochores (D) and duration of mitotic arrest (E) in nocodazole‐arrested cells treated with the MPS1 inhibitor AZ‐3146 (2.5 μM). In (D), treatment with MG132 (10 μM) was included to prevent mitotic exit after the addition of the MPS1 inhibitor, and kinetochore intensities were from 40 cells per condition, four experiments. Graph in (E) displays 50 cells per condition per experiment, three experiments.F–HEffects of locking PLK1 or PP2A on chromosome alignment and kinetochore–microtubules attachments. (F) Example immunofluorescence images to show the presence of chromosome misalignments (DAPI) and the presence of unattached kinetochores (MAD1) in MG132‐treated cells. The insets show magnifications of the outlined regions. Scale bars: 5 μm. Inset size: 1.5 μm. (G) Top panel: protocol used to visualise chromosome misalignment in fixed samples (see Materials and Methods for details). Bottom panel: graph showing mean frequencies (± SEM) of three experiments, 100 cells quantified per condition per experiment. (H) The number of kinetochores positive for MAD1 was measured as a readout of unattached kinetochores. The measurement was performed on 30–40 cells from four experiments, before and after a cold‐shock treatment to disrupt unstable kinetochore–microtubules attachments. Treatment with MG132 (10 μM) was included in (F–H) to prevent cells from exiting mitosis.IFrequencies of errors in anaphase in BUBR1 WT and B56γ cells (see also Fig EV2I–K). The graph shows mean frequencies (± SEM) of three experiments, 46–50 cells per experiment. Data information: Kinetochore intensities are normalised to BUBR1 WT control (B, C) at time point 0′ (D). Violin plots show the distributions of kinetochore intensities (B–D) or the number of MAD1‐positive kinetochores (H). For each violin plot, each dot represents an individual cell, the horizontal line represents the median and the vertical one the 95% CI of the median, which can be used for statistical comparison of different conditions (see Materials and Methods).

**Figure EV2:**
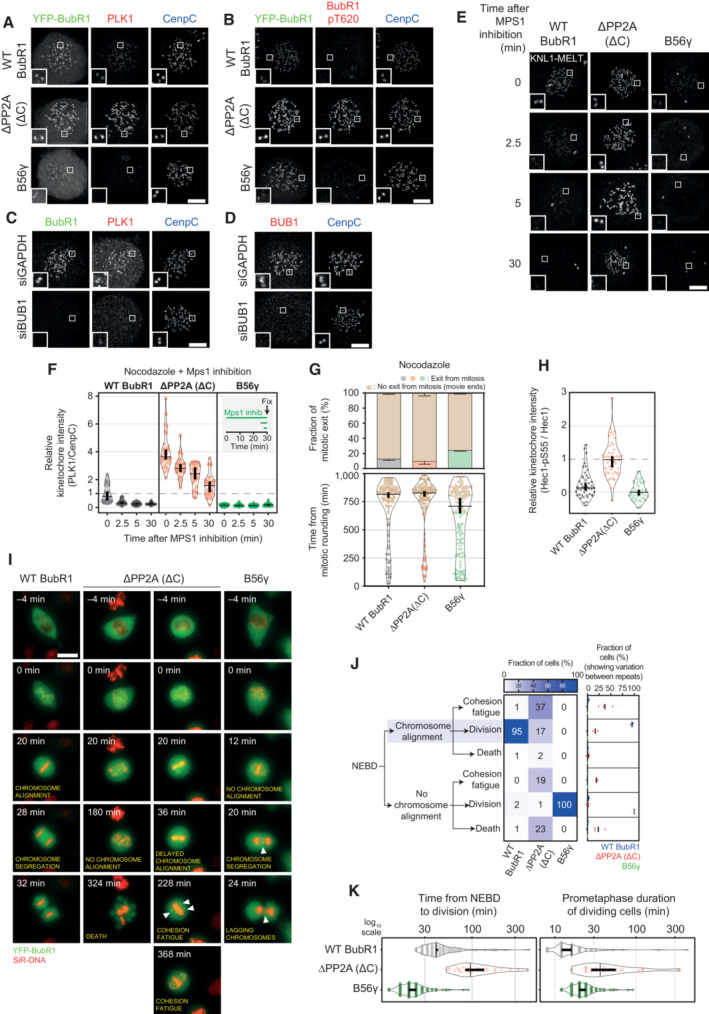
(Related to Fig 2). Molecular and phenotypic effects of locking PLK1 or PP2A on BUBR1 A–EExample immunofluorescence images of the kinetochore quantifications are shown in Fig 2B–D. Panel (D) shows the knockdown efficiency of BUB1, in relation to Fig 2B. The insets show magnifications of the outlined regions. Scale bars: 5 μm. Inset size: 1.5 μm.FEffects of locking PLK1 or PP2A on the levels of PLK1 at unattached kinetochores, in nocodazole‐arrested HeLa FRT cells expressing the indicated BUBR1 mutants and treated with the MPS1 inhibitor AZ‐3146 (2.5 μM). Treatment with MG132 (10 μM) was included to prevent mitotic exit after the addition of the MPS1 inhibitor. Kinetochore intensities from 30 cells, in three experiments.GEffects of locking PLK1 or PP2A on the duration of the mitotic arrest in nocodazole‐arrested HeLa FRT cells expressing the indicated BUBR1 mutants. Top panel: graph showing mean frequencies (± SEM) of cells that exit from mitosis. Bottom panel: distributions of the mitotic durations. Data from three experiments, 50 cells per condition per experiment.HEffects of locking PLK1 or PP2A on the levels of HEC1‐pS55 at unattached kinetochores, in nocodazole‐arrested HeLa FRT cells expressing the indicated BUBR1 mutants. Kinetochore intensities from 45 cells, in three experiments.I–KEffects of locking PLK1 or PP2A on the mitotic cell fate after nuclear envelope breakdown (NEBD). Panel (I) shows example images from live movies highlighting the most frequent mitotic cell fates. Cell fates are reported in yellow, and white arrows highlight defects during chromosome alignment or segregation. Scale bar: 20 μm. In (J), Left panel: heatmap showing the mean frequencies of cell fates after NEBD for each BUBR1 mutant—three experiments, 50 cells per condition per experiment. Right panel: percentages of the cell fates shown in the left panel from the three repeats of the experiment. For each distribution, the thick line corresponds to the mean values reported in the left panel. Panel (K) displays duration of mitosis (left panel) and prometaphase (right panel) of cells from (I) that divide after NEBD. Sample sizes: 146 cells in BUBR1 WT, 27 cells in ΔPP2A (ΔC) and 150 cells in B56γ. Data from three experiments. Data information: Kinetochore intensities are normalised to BUBR1 WT control at time point 0′ (F) or to BUBR1 ΔPP2A(ΔC) (H). Violin plots show the distributions of kinetochore intensities (F and H) or the distributions of the mitotic and prometaphase durations (G and K). For each violin plot, each dot represents an individual cell, the horizontal line represents the median and the vertical one the 95% CI of the median, which can be used for statistical comparison of different conditions (see Materials and Methods).

To test this, we first used BUBR1 mutants to lock either the PLK1‐ or PP2A‐bound states (Fig 2A) (Smith *et al*, 2019). To create the phosphatase‐bound situation, B56γ was tethered to the C‐terminus of BUBR1 in place of the KARD and pseudokinase domain (BUBR1^B56γ^). Note, that the pseudokinase can influence PP2A‐B56 recruitment but this is by regulating KARD phosphorylation (Gama Braga *et al*, 2020). To create the analogous kinase‐bound situation, the KARD was deleted together with the pseudokinase domain by removing the entire C‐terminus (BUBR1^ΔPP2A(ΔC)^). Figure 2B and C demonstrates that this system locks the separately bound states, as expected, because when the phosphatase is removed in BUBR1^ΔPP2A(ΔC)^ cells, BUBR1‐pT620 and PLK1 levels increase at kinetochores, as shown previously for removal of just the KARD domain (BUBR1^ΔPP2A(ΔK)^) (Cordeiro *et al*, 2020). Conversely, when the phosphatase is fused in BUBR1^B56γ^ cells, BUBR1‐pT620 and PLK1 levels are severely reduced at kinetochores. Note, this is equivalent to full removal of PLK1 from the BUB complex because kinetochores levels of PLK1 in this situation are not significantly different from the levels observed after BUB1 depletion (Figs 2B and EV2A–D). Note, that for this and all other violin plots, the thick vertical bars display 95% confidence intervals (CI) calculated around the median (thin horizontal lines). This allows easy statistical comparison between any treatment groups by eye because when the CI bars do not overlap, the difference between the medians is considered significant at *P* < 0.05.

Given the role of PLK1 and PP2A in regulating SAC and KT‐MT attachments, we hypothesised that the kinase‐dominant situation would lead to a strong SAC and hypostable KT‐MT attachments, whereas the phosphatase‐dominant situation would cause hyperstable KT‐MT attachments and a weak SAC (Fig 2A). To test the effects on SAC strength, we quantified KNL1‐MELT phosphorylation and mitotic exit in cells treated with nocodazole and partial MPS1 inhibition, which are sensitised SAC assays that can report increases or decreases in SAC strength (Santaguida *et al*, 2010; Saurin *et al*, 2011; Nijenhuis *et al*, 2014). BUBR1^ΔPP2A(ΔC)^ enhanced KNL1‐MELT phosphorylation, BUBR1 recruitment and SAC strength, as expected (Figs 2D and E, and EV2E). This was shown previously in BUBR1^ΔPP2A(ΔK)^ cells (Nijenhuis *et al*, 2014), and the enhanced SAC strength was due to elevated PLK1 activity because PLK1 inhibition could completely rescue these effects (Cordeiro *et al*, 2020). Conversely, the opposite effects were seen in BUBR1^B56γ^ cells, which showed reductions in MELT phosphorylation, BUBR1 recruitment and SAC strength (Fig 2D and E), consistent with the reduced PLK1 kinetochore levels in this situation (Figs 2B and EV2F). A reduction in SAC strength could also be observed in nocodazole alone (Fig EV2G).

To examine if KT‐MT attachments were similarly perturbed, we initially performed chromosome alignment assays when mitotic exit was blocked by MG132. This demonstrated severe misalignments in BUBR1^ΔPP2A(ΔC)^ cells (Fig 2F and G) due to unattached kinetochores, as expected and as shown previously in BUBR1^ΔPP2A(ΔK)^ cells (Suijkerbuijk *et al*, 2012; Kruse *et al*, 2013; Xu *et al*, 2013). This was associated with elevated N‐terminal phosphorylation of NDC80 (Fig EV2H), which is known to destabilise end‐on microtubule attachments (Wimbish & DeLuca, 2020), producing unattached kinetochores that stain positive for MAD1. Conversely, when B56γ is fused to BUBR1 in BUBR1^B56γ^ cells, although there was also a strong misalignment phenotype (Fig 2G), in this case the unaligned kinetochores were MAD1 negative (Fig 2F). This implies that they were stably attached to microtubules, which is consistent with the reduced NDC80 phosphorylation in this situation (Fig EV2H). We hypothesised that these reflect hyperstable KT‐MT attachments that were insensitive to the error‐correction machinery. In agreement, kinetochores of BUBR1^B56γ^ cells were resistant to detachment following cold‐shock treatment in comparison to BUBR1‐WT cells (Fig 2H, right panel). This effect was inverted in BUBR1^ΔPP2A(ΔC)^ cells, which rapidly dissociated from microtubules within the 10 min cold‐shock treatment, as expected (Suijkerbuijk *et al*, 2012; Kruse *et al*, 2013) (Fig 2H, left panel). The net result of mitotic cell fates is very different in both situations. In the kinase‐bound situation, the majority of the cells arrest in mitosis and undergo cell death or cohesion fatigue, whereas in the phosphatase‐bound situation, all cells undergo cell division without chromosome alignment, which in most cases leads to visible anaphase defects (Figs 2I and EV2I–K and Movies [Link], [Link]). Note that this is not simply a weak checkpoint because the prometaphase duration is actually extended in comparison to WT cells, indicating defective chromosome alignment as well as a weakened mitotic checkpoint (Fig EV2K).

In summary, PLK1 and PP2A are both needed on the BUB complex to ensure optimal SAC strength and KT‐MT stability and inducing situations where BUBR1 is only able to bind either the kinase or phosphatase produces distinct mitotic defects. We hypothesised that negative feedback between PLK1 and PP2A was crucial to balance their levels at BUBR1, and therefore to test this, we created mutants to specifically disrupt that feedback.

### The feedback loop between PLK1 and PP2A influences the levels and the activity of the kinase/phosphatase pair

**Figure 3:**
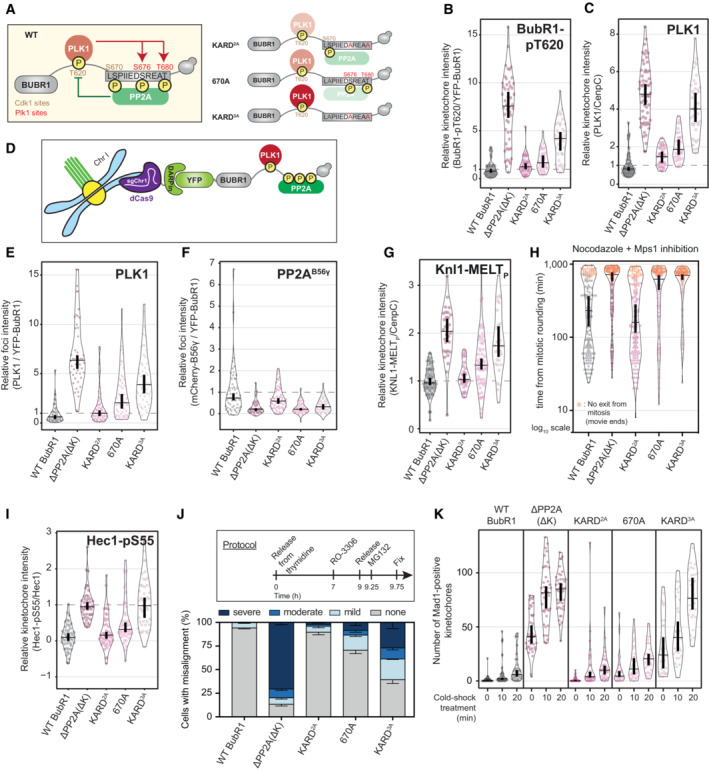
The cross‐talk between PLK1 and PP2A on BUBR1 ensures optimal strength of the SAC and KT‐MT ASchematic illustrating the PLK1/PP2A feedback loop on WT BUBR1 (left panel) and the BUBR1 alanine mutants on PLK1/CDK1 sites that were used to impair PP2A recruitment (right panel) and alter the PLK1/PP2A.B, CEffect of impaired PP2A recruitment to BUBR1 on levels of BUBR1‐pT620 (B) and PLK1 (C) at unattached kinetochores, in nocodazole‐arrested HeLa FRT cells expressing the indicated BUBR1 mutants. Kinetochore intensities from 30 to 60 cells, 3–6 experiments.D–FEffect of impaired PP2A recruitment to BUBR1 on levels of PLK1 (E) and mCherry‐B56γ (F) at ectopic foci on Chr I (D), in nocodazole‐arrested HeLa FRT cells expressing the indicated BUBR1 mutants. The schematics in (D) illustrate the experimental design to recruit YFP‐BUBR1 to the telomere of Chr I in HeLa FRT cells (see also Appendix Fig S1C and D and Materials and Methods for details). Panels (E) and (F) show PLK1 and mCherry‐B56γ levels at these chromatin foci. Foci intensities from 48 to 55 cells, three experiments.G, HEffect of impaired PP2A recruitment to BUBR1 on KNL1‐MELT phosphorylation (G) and duration of mitotic arrest (H) in nocodazole‐arrested HeLa FRT cells expressing the indicated BUBR1 mutants. Cells in (H) were treated with the MPS1 inhibitor AZ‐3146 (2.5 μM). Panel (G) displays kinetochore intensities of 30–70 cells per condition, six experiments. Panel (H) displays 50 cells per condition per experiment, three experiments.I–KEffect of impaired PP2A recruitment to BUBR1 on kinetochore levels of HEC1‐pS55 (I), chromosome alignment (J) and stability of kinetochore–microtubule attachments (K). Panel (I) shows levels of HEC1‐pS55 at unattached kinetochores, in nocodazole‐arrested HeLa FRT cells expressing the indicated BUBR1 mutants. Kinetochore intensities from 45 cells, three experiments. In (J), top panel is the protocol used to visualise chromosome misalignment in fixed samples (see Materials and Methods for details). Bottom panel includes graph showing mean frequencies of chromosome misalignments (± SEM) of three experiments, 100 cells quantified per condition per experiment. In (K), the number of kinetochores positive for MAD1 was measured as a readout of unattached kinetochores. The measurement was performed on 30–40 cells from 3 to 4 experiments, before and after a cold‐shock treatment to disrupt unstable kinetochore–microtubules attachments. In (J and K), treatment with MG132 (10 μM) was included to prevent cells from exiting mitosis. Data information: Kinetochore/foci intensities in (B–G) are normalised to the WT BUBR1 condition, while in (I) to the ΔPP2A(ΔK) condition. Violin plots show the distributions of kinetochore/foci intensities (B–G and I), the distributions of the mitotic duration (H) or the distributions of the number of MAD1‐positive kinetochores (K). For each violin plot, each dot represents an individual cell, the horizontal line represents the median and the vertical one the 95% CI of the median, which can be used for statistical comparison of different conditions (see Materials and Methods).

To impair the ability of PLK1 to recruit PP2A, we mutated the PLK1 sites BUBR1‐S676 and T680 to alanine (BUBR1 KARD^2A^). We also introduced an alanine mutation on the CDK1‐site BUBR1‐S670, either alone (BUBR1^670A^) or in combination with alanine mutants of the PLK1 sites (BUBR1 KARD^3A^). The prediction was that these mutants should progressively remove PP2A from BUBR1, thus skewing the balance towards the kinase‐dominant situation (Fig 3A).

Figure 3B and C and Appendix Fig S1A and B demonstrate that BUBR1‐pT620 and PLK1 are progressively increased at kinetochores as phosphorylation sites in the KARD domain are mutated. Mutation of just the CDK1 (670A) or PLK1 sites (KARD^2A^) partially elevates pT620 and PLK1, but combined mutation (KARD^3A^) causes p620/PLK1 levels to rise to a similar extent as the KARD deletion. PLK1 is recruited to various kinetochore locations (Chen *et al*, 2021; Nguyen *et al*, 2021; Singh *et al*, 2021), therefore to isolate BUBR1 away from the core kinetochore, we established a method to recruit YFP‐BUBR1 to a repetitive chromosomal locus on chromosome 1 using a dCas9‐DARPin that binds tightly to YFP (Fig 3D; and see Materials and Methods) (Brauchle *et al*, 2014). This in‐cell interaction assay reproduced the effect of KARD mutation on PLK1 localisation that was observed at kinetochores (Fig 3E and Appendix Fig S1C and D). Using the same assay, we demonstrate that KARD mutations cause reciprocal loss of PP2A‐B56 binding from BUBR1, as expected (Fig 3F and Appendix Fig S1C and D). These data are consistent with published *in vitro* data, which reported that phosphorylation of Ser670 increases BUBR1:B56‐binding affinity by ~9‐fold, phospho‐Ser676 increases it by ~5‐fold and that dual phospho‐Ser670/Ser676 enhances affinity ~38‐fold (Kruse *et al*, 2013). Together, this demonstrates that CDK1 and PLK1 are both important for BUBR1‐PP2A‐B56 interaction, and removing these kinase inputs into the KARD causes progressive decreases in PP2A‐B56 and increases in PLK1.

**Figure EV3:**
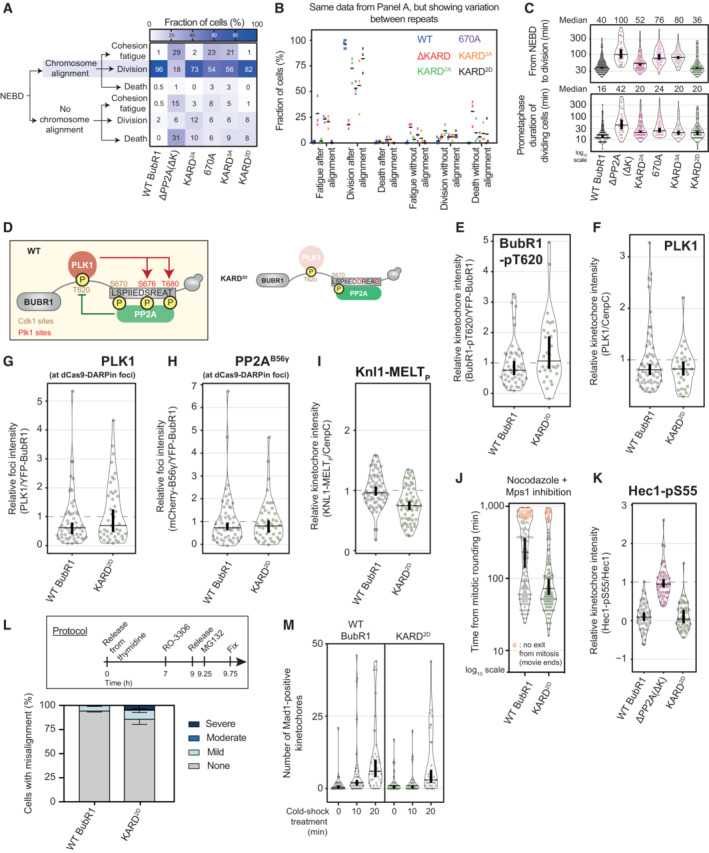
(Related to Fig 3). Molecular and phenotypic effects of BUBR1 mutants designed to increase or decrease PP2A levels A–CEvaluating the effect of indicated BUBR1 mutants on the mitotic cell fate after nuclear envelope breakdown (NEBD) (A and B) and on the duration of mitosis (C). The heatmap in panel (A) shows the mean frequencies of cell fates after NEBD in each condition—3–6 experiments, 50 cells per condition per experiment. Panel (B) shows the frequencies of the cell fates shown in (A) from the 3 to 6 repeats of the experiment. For each distribution, the thick line corresponds to the mean values reported in (A). Panel (C) shows the duration of mitosis (top panel) and prometaphase (bottom panel) of cells from (A) that divide after NEBD. Sample sizes: 295 cells in BUBR1 WT, 36 cells in ΔPP2A (ΔK), 127 cells in KARD^2A^, 89 cells in 670A, 93 cells in KARD^3A^ and 135 cells in KARD^2D^. Data from 3 to 6 experiments.DSchematic illustrating the PLK1/PP2A feedback loop on WT BUBR1 (left panel) and the BUBR1 aspartate mutant designed to enhance PP2A recruitment (right panel).E, FEffect of BUBR1 aspartate mutant on levels of BUBR1‐pT620 (E) and PLK1 (F) at unattached kinetochores in nocodazole‐arrested HeLa FRT cells expressing the indicated BUBR1 mutants. Kinetochore intensities from 30 to 60 cells, 3–6 experiments. Note that distributions of WT BUBR1 condition are the same shown in Fig 3B and C.G, HEffect of BUBR1 aspartate mutant on levels of PLK1 (G) and mCherry‐B56γ (H) at ectopic foci on Chr I, in nocodazole‐arrested HeLa FRT cells expressing the indicated BUBR1 mutants (see also Appendix Fig S1C and D and Materials and Methods for details). Foci intensities from 43 to 55 cells, three experiments. Note that distributions of WT BUBR1 condition are the same as shown in Fig 3E and F.I, JEffect of BUBR1 aspartate mutant on KNL1‐MELT phosphorylation (I) and duration of mitotic arrest (J) in nocodazole‐arrested HeLa FRT cells expressing the indicated BUBR1 mutants. Cells in panel (J) were treated with the MPS1 inhibitor AZ‐3146 (2.5 μM). Panel (I) displays kinetochore intensities of 60–70 cells per condition, six experiments. Panel (J) displays 50 cells per condition per experiment, three experiments. Note that distributions of WT BUBR1 condition are the same as shown in Fig 3G and H.K–MEffect of BUBR1 aspartate mutant on kinetochore levels of HEC1‐pS55 (K), chromosome alignment (L) and stability of kinetochore–microtubule attachments (M). Panel (K) shows levels of HEC1‐pS55 at unattached kinetochores, in nocodazole‐arrested HeLa FRT cells expressing the indicated BUBR1 mutants. Kinetochore intensities from 45 cells, three experiments. Note that distributions of WT BUBR1 and ΔPP2A(ΔK) conditions are the same as shown in Fig 3I. In (L), the top panel shows the protocol used to visualise chromosome misalignment in fixed samples (see Materials and Methods for details). Graph in bottom panel displays mean frequencies of chromosome misalignments (± SEM) of three experiments, 100 cells quantified per condition per experiment. Note that the mean frequencies of WT BUBR1 condition are the same as shown in Fig 3J. Panel (M) shows the number of kinetochores positive for MAD1 was measured as a readout of unattached kinetochores. The measurement was performed on 30–40 cells from 3 to 4 experiments, before and after a cold‐shock treatment to disrupt unstable kinetochore–microtubules attachments. Note that distributions of WT BUBR1 condition are the same as shown in Fig 3K. Treatment with MG132 (10 μM) was included in (L and M) to prevent cells from exiting mitosis. Data information: Kinetochore/foci intensities in (E–I) are normalised to the WT BUBR1 condition, while in (K) to the ΔPP2A(ΔK) condition. Violin plots show the distributions of kinetochore/foci intensities (E–I and K), the distributions of mitotic duration (C and J) or the distributions of the number of MAD1‐positive kinetochores (M) between cells. For each violin plot, each dot represents an individual cell, the horizontal line represents the median and the vertical one the 95% CI of the median, which can be used for statistical comparison of different conditions (see Materials and Methods).

This skewing of BUBR1 binding towards the kinase PLK1 is associated with increased KNL1‐MELT phosphorylation and SAC strength (Fig 3G and H and Appendix Fig S1E), as expected. Furthermore, it is also associated with increased Hec1 phosphorylation, chromosome misalignments, unstable KT‐MT attachments and defective chromosome segregation (Figs 3I–K and EV3A–C), consistent with a role of PP2A‐B56 in stabilising attachments (Suijkerbuijk *et al*, 2012; Kruse *et al*, 2013; Xu *et al*, 2013). Therefore, manipulating the kinase/phosphatase levels on BUBR1 to skew the balance towards PLK1 increases SAC strength and decreases KT‐MT attachment stability. We also mutated the PLK1 sites on the KARD to aspartate in an attempt to increase PP2A‐B56 and decrease PLK1, therefore skewing the balance towards a phosphatase‐dominant situation (Fig EV3D). Unfortunately, these mutants did not cause the predicted increases in B56γ or decreases in PLK1 (Fig EV3E–H and Appendix Fig S1A–D), either because aspartic acid does not mimic phosphorylated serine in recruiting B56 or the changes to enzyme levels are mild and not detectable by our assays. In support of the latter, we did note a slight, but significant, reduction in MELT phosphorylation and SAC strength (Fig EV3I and J and Appendix Fig S1E) and a slight increase in chromosome misalignments and stable KT‐MT attachments (Fig EV3A, B and K–M), consistent with a mild elevation of PP2A‐B56 and reduced PLK1 at kinetochores, which may not be detectable by our stead‐state recruitment assays.

In summary, a negative feedback loop between PLK1 and PP2A balances the levels of these enzymes on BUBR1, and probably also allows their dynamic association/disassociation over time to ensure proper SAC strength and KT‐MT attachment stability. CDK1 activity is crucial for establishing this feedback because it recruits PLK1 by phosphorylating BUBR1‐Thr620 and it helps to recruit PP2A by phosphorylating BUBR1‐Ser670. The resulting feedback between localised PLK1 and PP2A may be needed to allow dynamic or balanced PLK1/PP2A recruitment to each BUBR1 molecule, the entire KMN network or both. To address whether it helped set the correct levels of each enzyme on the KMN network, we created mutants that could modulate total BUB complex levels at their native positions within KNL1, but importantly, without altering the feedback between PLK1 and PP2A on each BUBR1 molecule. If BUBR1 was needed to set the right levels of PLK1/PP2A on the KMN network—the hub for SAC and KT‐MT regulation—then increasing or decreasing BUBR1 levels at this location should cause phenotypes associated with increased or decreased PLK1 and PP2A.

### The kinetochore levels of PLK1 and PP2A can be fined tuned by modulating the number of MELT motifs on KNL1

**Figure 4:**
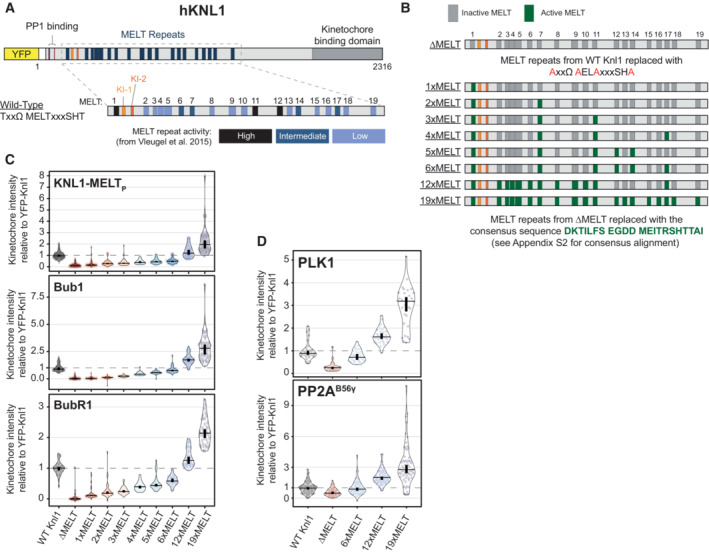
KNL1 levels of the BUB complex, PLK1 and PP2A scale with the number of active MELT motifs Scheme of human KNL1 with N‐terminal YFP‐tag. MELT motifs are represented with different shades of blue, according to the activity as evaluated in Vleugel *et al* (2015).Schematic illustrating the KNL1 mutants created with a different number of active MELTs (see Materials and Methods for details).Levels of KNL1‐pMELT, BUB1 and BUBR1 at unattached kinetochores, in nocodazole‐arrested HeLa FRT cells expressing the KNL1 mutants shown in panel (B). Kinetochore intensities from 40 to 60 cells, 3–5 experiments.Levels of PLK1 (top graph) and mCherry‐B56γ (bottom graph) at unattached kinetochores, in nocodazole‐arrested HeLa FRT cells expressing the indicated KNL1‐MELT mutants. Kinetochore intensities from 30 to 80 cells, 3–4 experiments. Kinetochore intensities are normalised to the WT KNL1 condition. Data information: Violin plots show the distributions of kinetochore intensities between cells. For each violin plot, each dot represents an individual cell, the horizontal line represents the median and the vertical one the 95% CI of the median, which can be used for statistical comparison of different conditions (see Materials and Methods).

BUBR1 is recruited to the KMN network by binding to phosphorylated MELT repeats on KNL1. Therefore, the total levels of PLK1 and PP2A at KNL1 are set by the number, sequence and phosphorylation status of these MELT repeats. Human KNL1 contains up to 19 MELT motifs, although many of these have degenerated and lost key amino acids needed for BUB complex binding (Tromer *et al*, 2015). Only eight MELTs have high or intermediate BUB affinity in wild‐type KNL1, the rest are low or undetectable and an average of 6–7 BUB1 molecules are bound to each KNL1 molecule on unattached kinetochores in human cells (Vleugel *et al*, 2015). The BUB1 binding strength and the specific MELT sequences, as determined in Vleugel *et al* (2015), are shown in Fig 4A and Appendix Fig S2A. We sought to modulate MELT numbers in a way that would allow BUB complex levels to be increased or decreased in a graded manner, thereby causing respective changes to PLK1 and PP2A levels. MELT numbers have been reduced before in human KNL1, but this was achieved using artificial KNL1 fragments that also modified the total length and position of these motifs within KNL1 (Vleugel *et al*, 2013; Zhang *et al*, 2014). This could affect the ability of PLK1 and PP2A to signal from these artificial fragments, therefore we sought to change MELT number within the context of full‐length KNL1.

To do this, we first mutated key residues that are crucial for BUB complex binding on all possible MELT motifs within full‐length KNL1: referred to as KNL1^ΔMELT^ (Fig 4B) (Vleugel *et al*, 2015). Then, we reintroduced an active MELT sequence, which our phospho‐MELT antibody reacts with, into specific numbers of these MELT motifs ranging from 1 to 19 (Fig 4B). This sequence is based on MELT13 and 17 and therefore predicted to have intermediate BUB affinity. Figure 4C and Appendix Fig S2B demonstrate that the kinetochore levels of phosphorylated MELT, BUB1 and BUBR1, are abolished in KNL1^ΔMELT^ cells, as expected. These levels are then increased in a graded manner as MELT number is increased, with 6xMELT recapitulating the closest to WT levels, and ≥ 12xMELTs producing artificially high BUB1/BUBR1 levels.

**Figure EV4:**
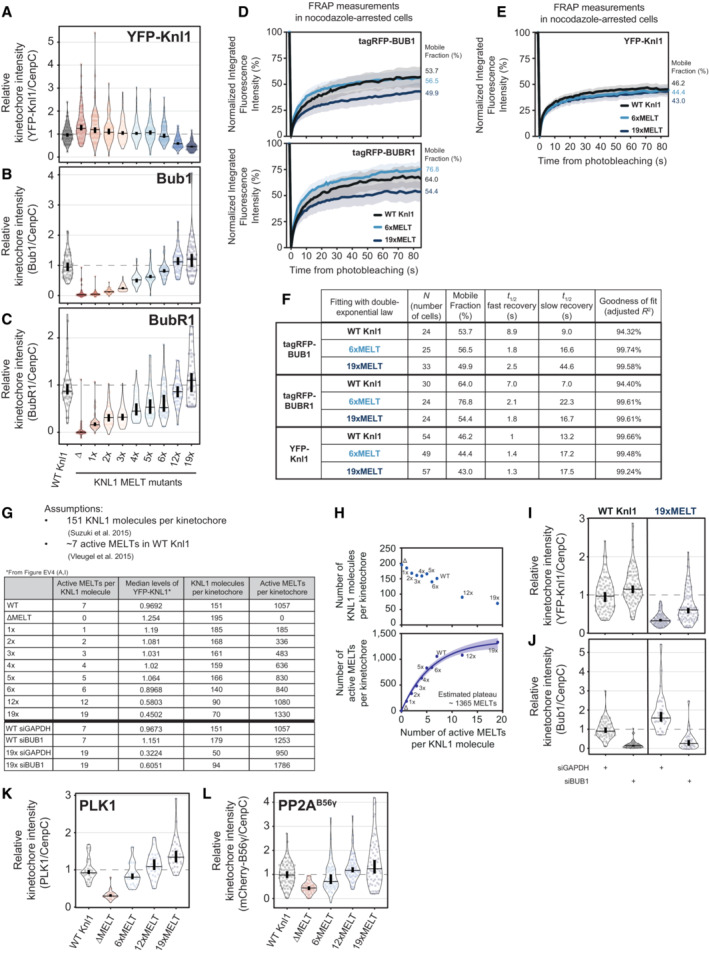
(Related to Fig 4). Recruitment of KNL1, BUB complex, PLK1 and PP2A to unattached kinetochores in KNL1 MELT mutants A–CLevels of YFP‐KNL1 (A), BUB1 (B) and BUBR1 (C) at unattached kinetochores relative to CenpC, in nocodazole‐arrested HeLa FRT cells expressing the indicated KNL1 MELT mutants. Measurements performed on the same cells are shown in Fig 4C. Kinetochore intensities from 40 to 120 cells, 3–10 experiments.D, EFluorescence recovery after photobleaching (FRAP) measurements of BUB1 (D, top graph), BUBR1 (D, bottom graph) and KNL1 (E) at unattached kinetochores, in nocodazole‐arrested HeLa FRT cells expressing WT, 6xMELT or 19xMELT KNL1 mutants. For each condition, the mean recovery is represented by a thick line, the 95% CI of the mean with a shaded area and the mobile fraction of the recovery as percentage at the end of each curve (see also (F), Appendix Fig S3 and Materials and Methods for details). Number of cells from three experiments for BUB1 FRAP: 24 for WT Knl1, 25 for 6xMELT and 33 for 19xMELT. Number of cells from three experiments for BUBR1 FRAP: 30 for WT Knl1, 24 for 6xMELT and 24 for 19xMELT. Number of cells from three experiments for KNL1 FRAP: 54 for WT Knl1, 49 for 6xMELT and 57 for 19xMELT.FParameters related to the FRAP curves reported in panels (D) and (E) after fitting with a double‐exponential law (see Materials and Methods for details).G, HEstimates of the number of KNL1 molecules and active MELT motifs per kinetochore, in nocodazole‐arrested HeLa FRT cells expressing the indicated KNL1 MELT mutants. The table in panel (G) reports the estimates per each KNL1 MELT mutants (see Materials and Methods for details). The graph in panel (H) shows the number of KNL1 molecules (top) or the number of active KNL1‐MELT motifs (bottom) plotted against the number of active MELTs per KNL1 molecule, using the data and the assumptions from (G). Data points in panel (H) (bottom) were fitted with an exponential plateau law (see Materials and Methods for details). The fitted curve is reported in the graph, together with the 95% CI of the fit and the estimated plateau. Goodness of fit: 98.69% (based on adjusted *R* ^2^).I, JThe levels of YFP‐KNL1 (I) and efficiency of BUB1 knockdown (J) at unattached kinetochores in nocodazole‐arrested HeLa FRT cells expressing WT or 19xMELT KNL1 mutants and knocked down for GAPDH or BUB1. Kinetochore intensities from 30 to 90 cells, 3–6 experiments.K, LLevels of PLK1 (K) and mCherry‐B56γ (L) at unattached kinetochores relative to CenpC, in nocodazole‐arrested HeLa FRT cells expressing the indicated KNL1 MELT mutants. The measurements were performed on the same cells shown in Fig 4D. Kinetochore intensities from 30 to 80 cells, 3–4 experiments. Data information: Kinetochore intensities are normalised to the WT KNL1 condition (A–C, K and L) or to the WT KNL1 siGAPDH condition (I and J). Violin plots show the distributions of kinetochore intensities. For each violin plot, each dot represents an individual cell, the horizontal line represents the median and the vertical one the 95% CI of the median, which can be used for statistical comparison of different conditions (see Materials and Methods).

Interestingly, total KNL1 kinetochore levels actually decreased when MELT numbers were increased beyond six MELTs (Fig EV4A). Therefore, artificially high MELT numbers increase BUB1/BUBR1 levels per KNL1 molecule (Fig 4C), but do not elevate total BUB1/BUBR1 levels per kinetochore (Fig EV4B and C). This is also associated with a reduced turnover of BUB1 and BUBR1 on and off kinetochores (Fig EV4D–F and Appendix Fig S3A and B). Based on previous predictions of the number of KNL1 molecules per kinetochore and the number of active MELTs per KNL1 (Suzuki *et al*, 2015; Vleugel *et al*, 2015), we find that cells can incorporate a maximum of ~1,000 active MELTs per kinetochore before KNL1 levels begin to be reduced (Fig EV4G and H). This reduction in KNL1 at high MELT numbers is alleviated by BUB1 depletion (Fig EV4G, I and J), which implies that KNL1 levels are restricted by limits on the number of MELT‐BUB interactions that can form at kinetochores, perhaps because of spatial constraints at the KMN network.

We hypothesised that this system would also cause a similar graded reduction/increase in PLK1 and PP2A levels at KNL1. In support, KNL1^ΔMELT^ reduced PLK1/PP2A, ≥ 12xMELTs increased PLK1/PP2A and 6xMELT recapitulating close to endogenous levels (Fig 4D). Again, the kinase/phosphatase levels are mainly increased on each KNL1 molecule, with only slight increases per kinetochore (Fig EV4K and L). In summary, this set of KNL1 mutants allows BUB complex levels to be precisely controlled at their native positions within full‐length KNL1. This can increase or decrease the levels of PLK1 and PP2A on each KNL1 molecule, crucially without modulating the intramolecular feedback between the two enzymes. We next examined whether the SAC strength and KT‐MT attachment stability were altered in these situations.

### Increasing the number of MELT motifs affects disassembly of the BUB complex from KNL1 following MPS1 inhibition

**Figure 5:**
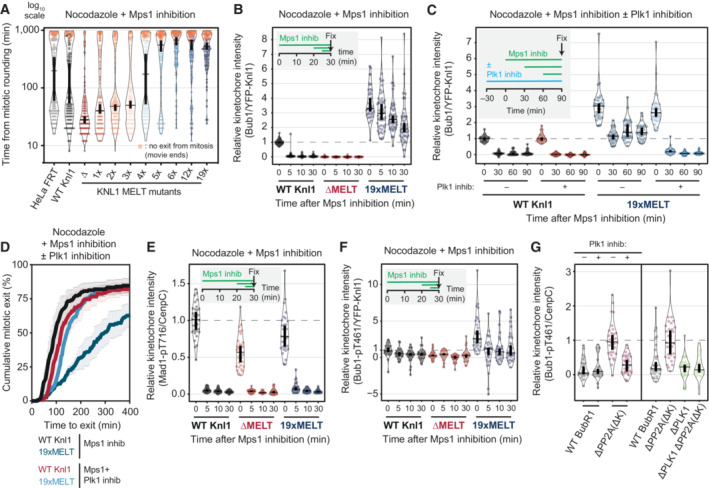
Increasing MELT number causes PLK1‐dependent SAC strengthening A, BEvaluation of the SAC signalling in KNL1‐MELT mutants, in terms of the duration of the mitotic arrest (A) and BUB1 levels at unattached kinetochores (B), in nocodazole‐arrested HeLa FRT cells expressing the indicated KNL1 mutants and treated with the MPS1 inhibitor AZ‐3146 (2.5 μM). Panel (A) displays 50 cells per condition per experiment, three experiments. Panel (B) shows kinetochore intensities from 30 cells, three experiments. Treatment with MG132 (10 μM) was included in (B) to prevent mitotic exit after the addition of the MPS1 inhibitor.C, DEvaluating the contribution of PLK1 kinase activity to sustained SAC signalling, in terms of BUB1 levels at unattached kinetochores (C), and the duration of the mitotic arrest (D), in nocodazole‐arrested cells expressing WT or 19xMELT KNL1 and treated with the MPS1 inhibitor AZ‐3146 (2.5 μM), with or without the PLK1 inhibitor BI‐2536 (100 nM). Panel (C) shows kinetochore intensities from 30 cells, three experiments. The graph in panel (D) shows mean (± SEM) of three experiments, 50 cells per condition per experiment. Treatment with MG132 (10 μM) was included in (C) to prevent mitotic exit after the addition of the MPS1 inhibitor.E, FLevels of MAD1‐pT716 (E) and BUB1‐pT461 (F) at unattached kinetochores in cells treated as in (B). Kinetochore intensities from 30 cells, three experiments. Treatment with MG132 (10 μM) was included to prevent mitotic exit after the addition of the MPS1 inhibitor.GEvaluation of the role of PLK1 on the levels of BUB1‐pT461 in nocodazole‐arrested HeLa FRT cells expressing the indicated BUBR1 mutants, treated or not with the PLK1 inhibitor BI‐2536 (100 nM) for 30′. Kinetochore intensities from 30 cells, six experiments. Data information: Kinetochore intensities are normalised to the WT KNL1 condition at time 0 (B, C and E, F) or to BUBR1 ΔPP2A(ΔK) condition (G). Violin plots show the distributions of mitotic duration (A) or the distributions of kinetochore intensities (B, C and E–G). For each violin plot, each dot represents an individual cell, the horizontal line represents the median and the vertical one the 95% CI of the median, which can be used for statistical comparison of different conditions (see Materials and Methods).

**Figure EV5:**
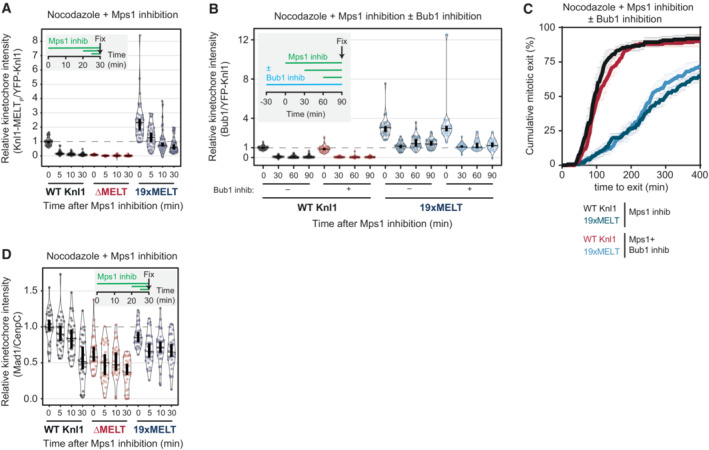
(Related to Fig 5). Molecular and phenotypic details of SAC signalling after modulation of KNL1 MELT numbers AEvaluation of KNL1‐pMELT levels at unattached kinetochores, in nocodazole‐arrested HeLa FRT cells expressing the indicated KNL1 mutants and treated with the MPS1 inhibitor AZ‐3416 (2.5 μM). Kinetochore intensities from 30 cells, three experiments. Treatment with MG132 (10 μM) was included to prevent mitotic exit after the addition of the MPS1 inhibitor.B, CEvaluation of the role of BUB1 kinase activity in sustaining the SAC signalling, in terms of BUB1 levels at unattached kinetochores (B) and the duration of the mitotic arrest (C), in nocodazole‐arrested HeLa FRT cells expressing the indicated KNL1 mutants and treated with the MPS1 inhibitor AZ‐3146 (2.5 μM), with or without the BUB1 inhibitor BAY‐1816032 (5 μM). Panel (B) shows kinetochore intensities from 30 cells, in three experiments. Note that distributions of WT KNL1 and 19xMELT without BUB1 inhibition are the same as shown in Fig 5C. The graph in panel (C) shows mean (± SEM) of three experiments, 50 cells per condition per experiment. Treatment with MG132 (10 μM) was included in (B) to prevent mitotic exit after the addition of the MPS1 inhibitor.DLevels of MAD1 at unattached kinetochores, in nocodazole‐arrested HeLa FRT cells expressing the indicated KNL1 mutants and treated with the MPS1 inhibitor AZ‐3416 (2.5 μM). Kinetochore intensities from 30 cells, three experiments. Treatment with MG132 (10 μM) was included to prevent mitotic exit after the addition of the MPS1 inhibitor. Data information: Kinetochore intensities are normalised to the WT KNL1 condition at time 0. Violin plots show the distributions of kinetochore intensities. For each violin plot, each dot represents an individual cell, the horizontal line represents the median and the vertical one the 95% CI of the median, which can be used for statistical comparison of different conditions (see Materials and Methods).

We first assessed SAC signalling on unattached kinetochores by examining the mitotic arrest in nocodazole‐arrested cells treated with 2.5 μM of the MPS1 inhibitor AZ‐3146. We chose this dose because it causes a partial override of the SAC, therefore mutants that either strengthened or weakened the SAC response could be identified. Figure 5A demonstrates that the SAC is weakened in KNL1 mutants that contains ≤ 3xMELT motifs, strengthened with ≥ 5xMELT motifs and indistinguishable from wild type in a mutant with 4xMELT motifs. The reduction in SAC strength with decreased MELT numbers is consistent with previous reports (Vleugel *et al*, 2013; Zhang *et al*, 2014), and predictable given the role of the MELTs in scaffolding SAC signalling. However, the effect of increasing MELT numbers has not previously been tested, and we hypothesised that enhanced PLK1 may help to strengthen the SAC under these conditions by enhancing MELT phosphorylation and BUB recruitment to KNL1 (Cordeiro *et al*, 2020). In agreement, MELT phosphorylation and BUB1 levels were elevated in KNL1‐19xMELT, and their dephosphorylation/removal upon MPS1 inhibition was significantly attenuated (Figs 5B and EV5A). Furthermore, PLK1 activity was amplifying MELT signalling in this situation because combined inhibition of MPS1 and PLK1 abolished BUB1 binding (Fig 5C) and allowed rapid mitotic exit (Fig 5D). Note that enhanced BUB1 catalytic activity in this situation does not contribute to these effects because the BUB1 inhibitor BAY‐1816032 (Siemeister *et al*, 2019) did not impact BUB1 recruitment or SAC strength (Fig EV5B and C). Therefore, increasing the number of MELT motifs causes the SAC platform on KNL1 to become less dependent on MPS1 activity, but more dependent on PLK1 activity.

The SAC signal relies on other MPS1 phosphorylation sites, in addition to the KNL1‐MELT motifs. MPS1 phosphorylates BUB1 on Thr461 to induce MAD1 interaction (Ji *et al*, 2017; Qian *et al*, 2017; Zhang *et al*, 2017; Fischer *et al*, 2022), and MAD1 on Thr716 to promote CDC20 binding (Faesen *et al*, 2017; Ji *et al*, 2017, 2018; Piano *et al*, 2021; Lara‐Gonzalez *et al*, 2021a), both of which help catalyse MCC assembly (Fischer, 2023). Therefore, we analysed if dephosphorylation of these sites following MPS1 inhibition was similarly perturbed in the KNL1‐19xMELT mutant. Figures 5E and EV5D demonstrate that kinetochore levels of MAD1 or MAD1‐pThr716 are not enhanced by increasing MELT number, implying that PLK1 is unable to sustain MAD1 phosphorylation or recruitment, as also shown by previous experiments in BUBR1^ΔPP2A(ΔK)^ cells (Cordeiro *et al*, 2020). BUB1‐pThr461 is maintained better on kinetochores following MPS1 inhibition in the KNL1‐19xMELT (Fig 5F), but this most likely reflects better preservation of BUB1 in this situation (Fig 5B), rather than delayed dephosphorylation of BUB1 specifically. It is important to note, however, that analysis of BUB1‐pThr461 in BUBR1^ΔPP2A(ΔK)^ cells demonstrated that BUBR1‐bound PLK1 contributes to enhanced Thr461 phosphorylation following PP2A removal (Fig 5G). The rise in pThr461 under these conditions has previously been attributed to reduced dephosphorylation from PP2A (Qian *et al*, 2017), however, these data imply that enhanced phosphorylation by PLK1 is at least partially responsible. We propose that PLK1 and PP2A work together to set BUB1‐pThr461 levels since this would explain why phosphorylation only changes in situations when that balance is skewed towards the kinase PLK1 (BUBR1^ΔPP2A^) or the phosphatase PP2A (Wang *et al*, 2023), but not when PLK1 and PP2A levels are both increased (KNL1‐19xMELT).

In summary, PLK1 collaborates with MPS1 to enhance KNL1‐MELT phosphorylation and BUB1‐Thr461 phosphorylation to support the SAC. Increasing the number of KNL1‐MELT motifs allows PLK1 to maintain MELT phosphorylation and BUB complex recruitment when MPS1 is inhibited. We speculate that enhancing substrate availability in this situation strengthens the autocatalytic feedback loop from BUB‐bound PLK1 to MELT phosphorylation and BUB recruitment.

### Increasing the number of MELT motifs inhibits disassembly of the BUB complex from KNL1 at metaphase and anaphase

**Figure 6:**
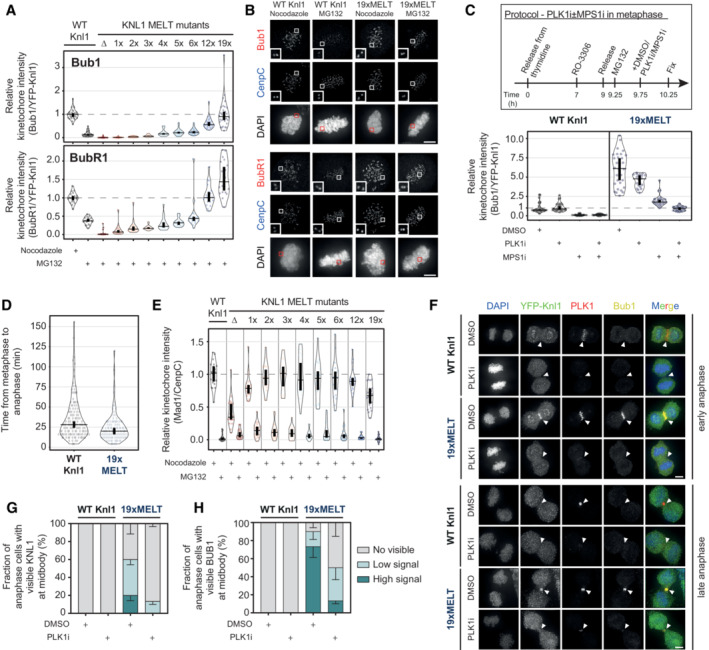
KNL1‐BUB dissociation is inhibited when MELT numbers are increased ALevels of BUB1 (top panel) and BUBR1 (bottom panel) at kinetochores, in nocodazole or MG132‐treated HeLa FRT cells expressing the indicated KNL1 mutants (see also Appendix Fig S4A for a detailed comparison between nocodazole and MG132 treatments in all the experimental conditions). Kinetochore intensities from 20 to 30 cells, in 2–3 experiments.BExample immunofluorescence images of some of the key kinetochore quantifications shown in (A) and Appendix Fig S4A. The insets show magnifications of the outlined regions. Scale bars: 5 μm. Inset size: 1.5 μm.CEvaluation of the contribution of PLK1 and MPS1 in sustaining BUB1 recruitment at kinetochores, in MG132‐treated HeLa FRT cells expressing the indicated KNL1 mutants. Top panel: protocol used to enrich cells in metaphase and to inhibit PLK1 ± MPS1 (using BI‐2536 100 nM and AZ‐3146 2.5 μM, respectively, see also Materials and Methods for details). Bottom panel: kinetochore levels of BUB1 per KNL1. Kinetochore intensities from 20 to 30 cells, in three experiments.DDuration of metaphase of HeLa FRT cells expressing the indicated KNL1 MELT mutants (see Fig 7A, Appendix Fig S5A and Materials and Methods for details). Number of cells from three experiments: 131 for WT KNL1 and 103 for 19xMELT.ELevels of MAD1 at kinetochores, in nocodazole or MG132‐treated HeLa FRT cells expressing the indicated KNL1 mutants. Kinetochore intensities from 20 cells, in two experiments.F–HEvaluation of the contribution of PLK1 in sustaining the recruitment of BUB1 and KNL1 to the midbody, in anaphase HeLa FRT cells expressing WT or 19xMELT KNL1 mutants and treated or not with PLK1 inhibitor (using BI‐2536 100 nM, see Materials and Methods for details). Panel (F) shows example immunofluorescence images of anaphase cells, in which the midbody is highlighted by white arrows. Scale bars: 5 μm. The graphs in (G) and (H) show mean frequencies (± SEM) of anaphase cells with visible KNL1 (G) or BUB1 (H) at the midbody. Data from 10 cells per experiment, in three experiments. Data information: Kinetochore intensities are normalised to the WT KNL1 nocodazole condition (A and E) or the WT KNL1 DMSO condition (C). Violin plots show the distributions of kinetochore intensities (A, C and E) or the distributions of the metaphase duration (D) between cells. For each violin plot, each dot represents an individual cell, the horizontal line represents the median and the vertical one the 95% CI of the median, which can be used for statistical comparison of different conditions (see Materials and Methods).

We next examined the effect of increasing MELT number on SAC silencing following KT‐MT attachment/tension, by analysing BUB1/BUBR1 on metaphase kinetochores. The BUB complex is normally reduced on kinetochores in metaphase as SAC signalling is silenced (Taylor *et al*, 1998; Howell *et al*, 2004; Shirnekhi *et al*, 2020). However, when MELT numbers are increased, the BUB complex fails to disassemble from KNL1, leaving similar BUB levels to those observed on unattached kinetochores of WT KNL1 cells (Fig 6A and B and Appendix Fig S4A). The enhanced BUB recruitment in this situation is also dependent on MPS1 and PLK1 activity (Fig 6C and Appendix Fig S4B), implying that microtubule attachment/tension does not lower kinase activities enough to disassemble the BUB complex from KNL1 when MELT numbers are increased. Mitotic duration is not extended under these conditions, however, demonstrating that the SAC is still silenced efficiently (Fig 6D). This can be explained by the fact that MAD1 is still removed by KT‐MT attachment despite the increased MELT number (Fig 6E), mostly likely because BUB1‐pT461 is not preserved and the kinetochore corona is still stripped by dynein. Interestingly, we noticed abnormal recruitment of KNL1 to the midbody during anaphase in the 19xMELT mutant, along with BUB1, BUBR1 and PLK1 (Fig 6F and Appendix Fig S4C–E), suggesting that the KNL1 signalling platform cannot even disassemble at anaphase when MELT numbers are high. In this situation, PLK1 inhibition can help to dissociate the complex from the midbody, consistent with the hypothesis that enhanced feedback from PLK1 prevents KNL1‐BUB disassembly in the 19xMELT mutant (Fig 6F–H).

In summary, PLK1 activity prevents the disassembly of the KNL1‐BUB complex when MELT numbers are increased, perhaps due to enhanced MELT phosphorylation by BUB‐bound PLK1. The SAC is still silenced efficiently under these conditions, most likely because MAD1 is still removed from attached kinetochores. We hypothesised that inefficient KNL1‐BUB disassembly might lead to defects in chromosome segregation because the BUB complex localises PP2A‐B56 to kinetochores, and elevated PP2A could stabilise microtubule attachments and impede the error‐correction process.

### The number of MELT motifs sets optimal PP2A‐B56 levels to ensure proper KT‐MT attachment regulation

**Figure 7:**
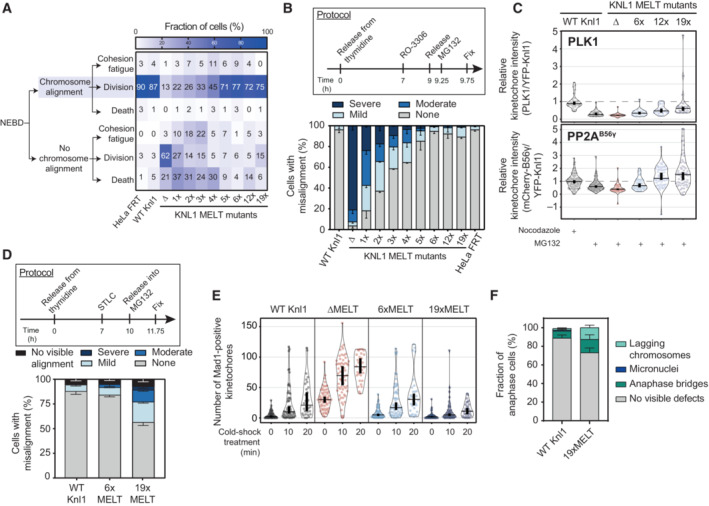
Increasing MELT number elevates PP2A at metaphase and stabilises KT‐MT attachments Mitotic cell fate after nuclear envelope breakdown (NEBD) in cells expressing the KNL1‐MELT mutants. The heatmap shows the mean frequencies of cell fates after NEBD in each condition: three experiments, 29–50 cells per condition per experiment (see also Appendix Fig S5A).Evaluating the effects on chromosome alignment in KNL1‐MELT mutants. Top panel: protocol used to visualise chromosome misalignment in fixed samples (see Materials and Methods for details). Bottom panel: mean frequencies (± SEM) of three experiments, 100 cells quantified per condition per experiment. Treatment with MG132 (10 μM) was included to prevent mitotic exit.Levels of PLK1 (top) and mCherry‐B56γ (bottom) at kinetochores, in nocodazole or MG132‐treated HeLa FRT cells expressing the indicated KNL1 mutants. Kinetochore intensities from 30 to 80 cells, in 3–4 experiments. Note that distributions of WT KNL1 in nocodazole are the same as shown in Fig 4D.Evaluating the effects on chromosome alignment in HeLa FRT cells, expressing the indicated KNL1‐MELT mutants and challenged to correct more KT‐MT attachment errors (using STLC 10 μM, see Materials and Methods for details). Top panel: protocol used to visualise chromosome alignment in fixed samples (see Materials and Methods for details). Bottom panel: mean frequencies of chromosome alignment errors (± SEM) from three experiments, 100 cells quantified per condition per experiment (see also Appendix Fig S5B for different time points after release from STLC). Treatment with MG132 (10 μM) was included to prevent mitotic exit.As a measure of unattached kinetochores, the number of kinetochores positive for MAD1 recruitment was measured. The measurement was performed in cells expressing the indicated KNL1‐MELT mutants, before and after a cold‐shock treatment to disrupt unstable kinetochore–microtubules attachments; 30–60 cells, 3–6 experiments. Treatment with MG132 (10 μM) was included to prevent mitotic exit.Evaluation of the defects in chromosome segregation in WT and 19xMELT KNL1 cells, enriched in anaphase after a release from a STLC block (see Materials and Methods for details). The graph shows mean (± SEM) of three experiments, 100 cells per condition per experiment. Data information: Kinetochore intensities are normalised to the WT KNL1 nocodazole condition. Violin plots show the distribution of kinetochore intensities (C) or the distributions of the number of MAD1‐positive kinetochores (E) between cells. For each violin plot, each dot represents an individual cell, the horizontal line represents the median and the vertical one the 95% CI of the median, which can be used for statistical comparison of different conditions (see Materials and Methods).

To examine if the KT‐MT attachment process was affected by altering KNL1‐MELT numbers, we initially performed live cell imaging to quantify chromosome segregation. Figure 7A and Appendix Fig S5A demonstrate that in situations with less than five MELT motifs, chromosome alignment is perturbed and cells either die in mitosis, undergo cohesion fatigue or divide with unaligned chromosomes. To analyse chromosome alignment more carefully under these conditions, we performed fixed assays in the presence of MG132. Figure 7B shows that KNL1^ΔMELT^ causes severe chromosome misalignments, which are indistinguishable from the BUBR1^ΔPP2A^ (Fig 2G), implying that lack of PP2A‐B56 recruitment to KNL1 is the primary cause of the phenotype in both situations. These misalignments are progressively rescued by increasing MELT number until an optimal number of 6xMELT motifs, which appeared indistinguishable from wild‐type KNL1 (KNL1^WT^). Note, this is also the situation that rescued PLK1 and PP2A‐B56 to close to wild‐type levels on either unattached or attached kinetochore (Figs 4D and 7C). When MELT number is increased beyond 6xMELT, PLK1 and PP2A‐B56 levels are elevated at KNL1 at unattached and attached kinetochores (Figs 4D and 7C), and there was a small but consistent increase in mild misalignments (Fig 7A and B). If this was due to hyperstable KT‐MT attachments, as predicted, then these defects should become more apparent when cells are challenged to correct more KT‐MT attachment errors. Therefore, we performed similar alignment assays after washout from an Eg5 inhibitor, STLC, to elevate KT‐MT attachment errors (Kapoor *et al*, 2000). Chromosome alignment assays at different time points following washout demonstrated that the speed and total levels of chromosome alignment were impaired under conditions with 19xMELT motifs (Fig 7D and Appendix Fig S5B). This is likely due to hyperstable KT‐MT attachments because cold‐shock treatment was less able to detach kinetochore fibres in KNL1‐19xMELT cells, in comparison to KNL1‐WT cells, as assayed by MAD1 accumulation at unattached kinetochores (Fig 7E). This was in contrast to KNL1^ΔMELT^ cells, which contained many unattached kinetochores already under basal conditions, and these numbers increased, as expected, following cold‐shock treatment. Again, the proportion of unattached kinetochores in this situation was indistinguishable from that observed in BUBR1^ΔPP2A^ cells (Fig 2H), further supporting the conclusion that PP2A‐B56 loss is the primary cause of the alignment phenotypes in KNL1^ΔMELT^ cells. Therefore, the number of MELT motifs is crucial for determining the stability of KT‐MT attachments, most probably by setting the correct levels of PP2A‐B56 on the KMN network. When MELT numbers are increased, the BUB complex and PP2A‐B56 are elevated at KNL1 on unattached kinetochores, and they also fail to disassemble from this complex following KT‐MT attachment/tension, both of which likely limit the ability of Aurora B to dissociate improperly attached kinetochores. This is important to prevent chromosome segregation errors in anaphase because the KNL1‐19xMELT mutant also increased the proportion of lagging chromosomes and anaphase bridges (Fig 7F), indicative of impaired error correction.

## Discussion

**Figure 8:**
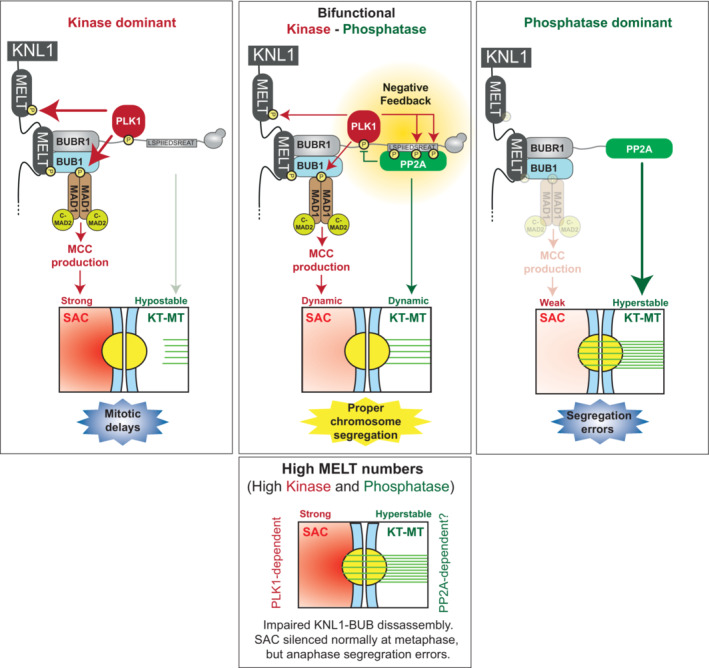
Schematic to illustrate how kinase–phosphatase coupling on the BUB complex integrates two mitotic processes to safeguard chromosome segregation PLK1 and PP2A are engaged in a negative feedback loop on BUBR1 which sets PLK1/PP2A levels on the BUB complex. This bifunctional kinase–phosphatase module is needed to ensure the correct strength and dynamics of the SAC and KT‐MT attachments, thus promoting proper chromosome segregation (middle panel). Disrupting the feedback to lock either the kinase (left panel) or the phosphatase (right panel) alters the bifunctionality of this module, affecting the molecular targets at kinetochore and causing chromosome segregation errors or mitotic delays.

Here, we have characterised a bifunctional kinase–phosphatase module on the BUB complex that functions to optimise the strength and dynamics of the SAC and KT‐MT attachments. The kinase PLK1 recruits and phosphorylates the BUB complex to support the SAC, whereas the phosphatase PP2A‐B56 antagonises these actions to help silence the SAC, while at the same time, antagonising Aurora B to stabilise KT‐MT attachments. An intramolecular negative feedback loop between PLK1 and PP2A‐B56 is crucial to balance the relative levels of these enzymes and promote accurate and timely chromosome segregation. This allows PLK1 to amplify SAC signalling without locking the SAC signal on, ensuring that the SAC signal remains strong, but crucially, still responsive to declining MPS1 activity. In turn, PP2A‐B56 can stabilise initial KT‐MT attachments without hyperstabilising them and impeding the KT‐MT error‐correction process. Therefore, the SAC and KT‐MT attachment processes can remain dynamic and responsive to tension. These dynamics are perturbed when MELT numbers are increased, perhaps due to enhanced feedback from PLK1 to BUB recruitment, which increases steady‐state KNL1‐BUB levels and inhibits the dissociation of this complex from aligned kinetochores. The SAC is still silenced under these conditions, but KT‐MT error correction is perturbed, most likely due to enhanced PP2A‐B56 activity at KNL1. Therefore, the MELTs have evolved together with the PLK1‐PP2A module to regulate BUB complex dynamics at KNL1, and thereby ensure accurate chromosome segregation. The final model is described in Fig 8.

The PLK1/PP2A module described here is recruited to kinetochores via the MELT motifs on KNL1, which is an important signalling hub for mitotic regulation (Ghongane *et al*, 2014). Previous studies on truncated versions of human KNL1 showed that the number of MELT repeats sets the kinetochore levels of the BUB complex to influence SAC signalling and chromosome alignment (Vleugel *et al*, 2013; Zhang *et al*, 2014). We expand on this here, by examining SAC signalling/strength, and chromosome alignment/KT‐MT stability in a range of MELT mutants that span less than, and crucially, more than the optimal number. Importantly, this analysis was also performed within the context of full‐length KNL1. In agreement with previous studies (Vleugel *et al*, 2013; Zhang *et al*, 2014), our data show that > 3 MELT motifs are required for normal mitotic progression and we demonstrate that this relates to both SAC strength (Fig 5) and KT‐MT stability (Fig 7). The ability of a KNL1‐NC fusion containing just one MELT motif to fully support the SAC in the Vleugel *et al* (2013) study may be related to the artificially shortened KNL1 because within the context of full‐length KNL1, the first MELT motif exhibits a weaker SAC response (Fig 5). By carefully analysing the effect of increasing and decreasing MELT numbers, we show that these are important to balance strength and dynamics of the SAC and KT‐MT attachments. When too many MELTs are present, this strengthens KNL1‐BUB interactions, enhances PLK1‐PP2A levels and increases SAC strength and KT‐MT stability. These are associated with reduced responsiveness of the BUB complex to microtubule attachment or MPS1 inhibition, which has consequences for the disassembly of the SAC platform and KT‐MT stability at metaphase. PLK1 inhibition can disassemble the KNL1‐BUB complex and weaken the SAC under these conditions, implying that elevated PLK1 activity at KNL1 underlies these defects. In support, similar phenotypes have also been observed when PLK1 is tethered directly to NDC80, another component of the KMN network, to prevent its decline on metaphase kinetochores (Liu *et al*, 2012). Therefore, KNL1‐BUB interaction via the MELT motifs is crucial for signalling to the SAC and stabilising initial KT‐MT attachments in prometaphase, but following bipolar KT‐MT attachment, this complex must disassemble to shut off the SAC and ensure that those KT‐MT attachments remain dynamic. Our work demonstrates that the number of MELT must be finely balanced to allow this rapid KNL1‐BUB disassembly at metaphase.

The number and sequence of the MELTs are highly variable in eukaryotic KNL1, which has evolved rapidly by iterative cycles of MELT expansion and diversification (Tromer *et al*, 2015). This has allowed the kinetochore levels and affinity of the BUB complex to rapidly evolve, and this has been shown to balance the strength and responsiveness of the SAC in *budding yeast* (Roy *et al*, 2020). We build on that here to show that the acquisition of a PLK1‐PP2A‐binding module on the BUB complex in metazoa adds a further level of complexity, but the overall importance of the MELTs in balancing strength and responsiveness of kinetochore signalling remains a common feature. We speculate that a crucial role for the MELTs across species is to balance the kinase and phosphatase activities needed to activate and extinguish kinetochore signalling events. In budding yeast, the key kinase/phosphatase balance in terms of MELT phosphorylation and SAC signalling is MPS1/PP1 (Roy *et al*, 2020). However, in human cells, PLK1 cooperates with MPS1, and PP2A‐B56 cooperates with PP1, which together form a network that regulates both SAC signalling and KT‐MT attachments (Saurin, 2018). Our work implies PLK1‐PP2A interplay on BUBR1 is a central component of this network, but in future, it will be important to understand how this impact MPS1 and PP1 activity, and crucially Aurora B activity, to control chromosome segregation. In this respect, it is important to point out that PLK1 and PP2A are likely to control more than just the phosphorylation sites and processes outlined here. For example, BUBR1‐bound PP2A‐B56 has recently been implicated in stabilising KT‐MT attachments by antagonising MPS1 localisation to end‐on attached kinetochores (Hayward *et al*, 2022). Furthermore, in *C. elegans*, BUB1‐bound PLK1 phosphorylates the BUB1 ABBA motif to both enhance CDC20 binding and promote CDC20 dephosphorylation/activation, which together helps to activate the APC/C and allow timely mitotic exit (Houston *et al*, 2023). *C. elegans* BUB1 contains similar PLK1‐ and PP2A‐binding motifs to BUBR1, and these are important for chromosome alignment and segregation during meiosis I (Bel Borja *et al*, 2020; Taylor *et al*, 2023). Cross‐talk between PLK1 and PP2A has not been evaluated in worms, but it is notable that a PLK1 phosphorylation site is preserved in the PP2A‐B56‐binding site. Therefore, it is tempting to speculate that feedback between PLK1/PP2A may regulate the recruitment and activation of CDC20 to control mitotic exit, in a manner that is independent of its effects on the SAC and KT‐MT attachments. Similarly, PLK1‐PP2A cross‐talk may be important for chromosome segregation during meiosis I. Testing these possibilities will be important future goals.

Finally, it should be pointed out that the “interplay” between kinase and phosphatase becomes even more complex when one considers how that may coordinate phosphorylation and dephosphorylation events over time on individual molecules. Rapid cycles of phosphorylation and dephosphorylation can impart unique properties to a signal response, such as responsiveness, as explained here (Gelens & Saurin, 2018). For example, apparently “futile” on/off cycles are used in EGFR signalling to provide a response that can rapidly change states (Kleiman *et al*, 2011). It is possible that similar futile cycles are crucial at kinetochores to allow signals to change states quickly upon microtubule attachment/tension. The picture may become even more complex when one considers whether kinase and phosphatase are separated in time and/or space on individual molecules or complexes. For example, BUB complexes may flip between the PLK1‐ or PP2A‐bound state, with phosphorylation/dephosphorylation reactions driving cycles of PLK1/PP2A binding and release, as discussed in Gelens and Saurin (2018), or perhaps BUB1‐MAD1 binding a release, by regulating BUB1‐pThr461. These cycles may even work on specific subsets of BUB complexes at certain times. For example, the BUB1/BUB3 complex may recruit PLK1 (via BUB1‐pT609; Qi *et al*, 2006; Cordeiro *et al*, 2020) to phosphorylate BUB1‐pT461 and the KNL1‐MELTs, before BUBR1 association allows PP2A‐B56 recruitment to remove PLK1 and reverse these events, perhaps at a specific stage of MCC assembly. This might explain why phosphatase activity must be spatially separated onto BUBR1, and not BUB1, for the checkpoint to function correctly in human cells (Wang *et al*, 2023). Understanding whether PLK1 and PP2A are recruited to specific BUB complexes at certain times will be an important future goal. It will also be important to understand whether the PLK1‐PP2A feedback loop operates only at specific subcellular localisation or at certain times. The key role played by CDK1 in recruiting both PLK1 and PP2A implies the feedback is likely only active from mitotic entry until anaphase. However, Cyclin B/CDK1 is recruited to unattached kinetochores where it binds to MAD1 (Alfonso‐Perez *et al*, 2019; Allan *et al*, 2020; Jackman *et al*, 2020) Therefore, it is possible that localised CDK1 activity is needed to initiate the feedback specifically at unattached kinetochores, and not, for example, on APC/C‐MCC complexes. In this case, the feedback could also be extinguished or reduced at metaphase when Cyclin B is removed from kinetochores.

In summary, the bifunctional kinase–phosphatase module we identify here is crucial for integrating two key mitotic processes at the kinetochore. It remains to be determined exactly how this module functions to integrate these two processes, but it is clear that feedback between the enzymes is crucial, as is their ability to signal concurrently during prometaphase. A similar PLK1/PP2A‐binding module has recently been identified on APC1, which allows these enzymes to engage in feedback and control activity of the anaphase‐promoting complex (Fujimitsu & Yamano, 2021). Comparing and contrasting these seemingly different situations may reveal common features that help to explain why these fascinating kinase–phosphatase co‐recruitment modules have evolved.

## Materials and Methods

### Cell culture and reagents

All cell lines used in this study were derived from HeLa Flp‐in cells (a gift from S Taylor, University of Manchester, UK) (Tighe *et al*, 2008), which were authenticated by STR profiling (Eurofins). Cells were cultured in full‐growth media—DMEM supplemented with 9% FBS and 50 μg/ml penicillin/streptomycin. While doing fluorescence time‐lapse analysis, cells were cultured in Leibovitz's L‐15 media (900 mg/L D+ galactose, 5 mM Sodium pyruvate and no phenol red) supplemented with 9% FBS and 50 μg/ml penicillin/streptomycin, or DMEM (no phenol red) supplemented with 9% FBS and 50 μg/ml penicillin/streptomycin. Every 4–8 weeks, cells were screened to ensure a mycoplasma‐free culture.

Doxycycline (1 μg/ml), STLC (S‐trityl‐L‐cysteine: 10 μM), thymidine (2 mM), nocodazole (3.3 μM), MG132 (10 μM) and the MPS1 inhibitor AZ‐3146 (2.5 μM) were purchased from Sigma Aldrich; puromycin (1 mg/ml) and hygromycin B (200 μg/ml) from Santa Cruz Biotechnology; RO‐3306 (10 μM) from Tocris; the PLK1 inhibitor BI‐2536 (100 nM) from SelleckBio; the BUB1 inhibitor BAY‐1816032 (5 μM) from MedChemExpress; the SiR‐DNA far‐red DNA probe (1:10,000) from Spirochrome.

### Plasmids and cloning

pcDNA5‐YFP‐BUB1^WT^ and pcDNA5‐YFP‐BUBR1^WT^ expressing an N‐terminally YFP‐tagged and siRNA‐resistant wild‐type BUB1 or BUBR1 were described previously (Nijenhuis *et al*, 2014). pcDNA5‐YFP‐BUBR1^ΔPP2A(ΔK)^ (also called BUBR1^ΔKARD^), lacking amino acids 664–681 of BUBR1, was described previously (Nijenhuis *et al*, 2014). pcDNA5‐YFP‐BUBR1^ΔPP2A(ΔC)^, pcDNA5‐YFP‐BUBR1^B56γ^ (also called BUBR1^ΔCT^‐B56γ_1_), pcDNA5‐YFP‐BUBR1^T620A^ (also called BUBR1^ΔPLK1^) and pcDNA5‐YFP‐BUBR1^ΔPP2A‐T620A^ (also called BUBR1^ΔPP2A + ΔPLK1^) were described previously (Smith *et al*, 2019; Cordeiro *et al*, 2020). Site‐directed mutagenesis with specific primers (Sigma‐Aldrich) was performed on pcDNA5‐YFP‐BUBR1^WT^ to generate: pcDNA5‐YFP‐BUBR1^KARD‐2A^ (forward: 5′‐GCTGAGCCCAATTATTGAAGACGCTCGTGAAGCCGCACACTCCTCTGGCTTCTCTGG‐3′; reverse: 5′‐CCAGAGAAGCCAGAGGAGTGTGCGGCTTCACGAGCGTCTTCAATAATTGGGCTCAGC‐3′), pcDNA5‐YFP‐BUBR1^KARD‐2D^ (forward: 5′‐GCTGAGCCCAATTATTGAAGACGATCGTGAAGCCGACCACTCCTCTGGCTTCTCTGG‐3′; reverse: 5′‐CCAGAGAAGCCAGAGGAGTGGTCGGCTTCACGATCGTCTTCAATAATTGGGCTCAGC‐3′) and pcDNA5‐YFP‐BUBR1^670A^ (forward: 5′‐GACTCTCAGCATCAAGAAGCTGGCACCAATTATTGAAGACAGTCG‐3′; reverse: 5′‐CGACTGTCTTCAATAATTGGTGCCAGCTTCTTGATGCTGAGAGTC‐3′). Cloning of pcDNA5‐YFP‐BubR1^KARD‐3A^ (carrying alanine mutations on S670, S676 and T680 sites) was described previously (Suijkerbuijk *et al*, 2012). pcDNA5‐FLAG‐tagRFP‐BUB1^WT^ and pcDNA5‐FLAG‐tagRFP‐BUBR1^WT^ were generated by restriction cloning using KpnI and AgeI to replace the YFP present in pcDNA5‐YFP‐BUB1^WT^ and pcDNA5‐YFP‐BUBR1^WT^ with FLAG‐tagRFP.

All KNL1 constructs used in this study were derived from the plasmid pcDNA5‐YFP‐KNL1^WT^, which expresses a siRNA‐resistant and N‐terminally YFP‐tagged wild‐type KNL1 previously described (Smith *et al*, 2019). To create a full‐length KNL1 with variable number of active MELT motifs, we used gene synthesis to replace the original motifs **T**xxΩ **M**EL**T**xxxSH**T**—which have variable lengths depending on their position in KNL1 (Vleugel *et al*, 2013)—with the amino acid sequence DK**T**ILFS EGDD **M**EI**T**RSH**T**TAI. This consensus sequence was designed based on conservation (Vleugel *et al*, 2013), medium affinity to recruit BUB complex (Vleugel *et al*, 2015) and optimal recognition using the pMELT antibody (pMELT‐13/17, Thr 943 and Thr 1155, raised using the peptide MEIpTRSHTTALEC—Nijenhuis *et al*, 2014). The nucleotide sequence of the active MELT repeats was varied as much as possible in the synthetised KNL1 constructs to avoid recombination of the plasmid during bacterial culture. To inactivate the MELT repeats and create KNL1^ΔMELT^, key threonines and methionines (or serines if present) in all 19 MELT motifs were mutated to alanine (**T**xxΩ **M**EL**T**xxxSH**T** was mutated to **A**xxΩ **A**EL**A**xxxSH**A**). All KNL1 constructs were subcloned by restriction cloning except when indicated. The pcDNA5‐YFP‐KNL1^ΔMELT^ and pcDNA5‐YFP‐KNL1^6xMELT^ (active MELT motifs at positions 1, 7, 11, 12, 14 and 17) were generated by inserting synthesised DNA fragments (Bio Basic Inc) in the backbone pcDNA5‐YFP‐KNL1^WT^ at restriction sites XmaI/Bsu36I and XhoI/BbvCI, respectively. To permit downstream cloning of different constructs, silent mutations were introduced during DNA synthesis to insert extra restriction sites in the KNL1 constructs, including XmaI before MELT‐1, BssHII before MELT‐6, HpaI before MELT‐8, ApaI before MELT‐12, XcmI before MELT‐14 and Bsu36I before MELT‐19. pcDNA5‐YFP‐KNL1^19xMELT^ was generated by Gibson assembly also using a synthesised DNA fragment (Bio Basic Inc) to insert extra MELT motifs in the pcDNA5‐YFP‐KNL1^6xMELT^ plasmid between PmlI and BbvCI sites. pcDNA5‐YFP‐KNL1^1xMELT‐1^ (active MELT motif at position 1), pcDNA5‐YFP‐KNL1^1xMELT‐17^ (active MELT motif at position 17), pcDNA5‐YFP‐KNL1^2xMELT^ (active MELT motifs at positions 1 and 7) and pcDNA5‐YFP‐KNL1^3xMELT^ (active MELT motifs at positions 1, 7 and 11) were created by inserting DNA fragments from pcDNA5‐YFP‐KNL1^6xMELT^ in the backbone pcDNA5‐YFP‐KNL1^ΔMELT^ using restriction sites XhoI/PmlI, AvrII/Bsu36I, XhoI/BlpI and XhoI/ApaI, respectively (KNL1^2xMELT^ generated using Gibson assembly with a fragment amplified by PCR). pcDNA5‐YFP‐KNL1^4xMELT^ (active MELT motifs at positions 1, 7, 11 and 17) and pcDNA5‐YFP‐KNL1^5xMELT^ (active MELT motifs at positions 1, 7, 11, 12 and 14) were created by inserting fragments from pcDNA5‐YFP‐KNL1^1xMELT‐17^ or pcDNA5‐YFP‐KNL1^ΔMELT^ in the backbone pcDNA5‐YFP‐KNL1^6xMELT^ using ApaI/Bsu36I or AvrII/BbvCI sites, respectively. pcDNA5‐YFP‐KNL1^12xMELT^ (active MELT motifs at positions 1–11 and 17) was subcloned by replacing part of pcDNA5‐YFP‐KNL1^19xMELT^ (between ApaI/BbVCI restriction sites) with a fragment from pcDNA5‐YFP‐KNL1^1xMELT‐17^. All plasmids were fully sequenced to verify the transgene was correct.

Cloning of pMESV_Ψ_‐mCherry‐B56γ_1_ and pHAGE‐TO‐dCas9‐DARPin‐FLAG plasmids has been described previously (Smith *et al*, 2019; Vallardi *et al*, 2019). The gRNAs targeting repetitive regions on Chr I (region p36, 2581275–2634211, ~256 repetitive regions), Chr III (region q29, 195199022–195233876, ~500 repetitive regions) or Chr XIII (region q34, 112930173–112968847, ~350 repetitive regions) (Ma *et al*, 2016; Wang *et al*, 2018) were generated by PCR mutagenesis to introduce the gRNA sequences (Chr I: GATGCTCACCT, Chr III: TGATATCACAG or Chr XIII: ACCATTCCTTC) (Ma *et al*, 2016) into a pU6 vector containing the guide RNA scaffold for the CRISPR/Cas9 system.

### Gene expression

HeLa Flp‐in cells were stably generated to allow doxycycline‐inducible expression of all constructs. Constructs were transfected with the relevant pcDNA5/FRT/TO plasmid and the Flp recombinase pOG44 (Thermo Fisher) using Fugene HD (Promega) according to the manufacturer's instructions. Subsequently, cells were selected for stable integrants at the FRT locus using hygromycin B for at least 2 weeks. Cells expressing mCherry‐B56γ_1_ were generated by viral integration of pMESV_Ψ_‐mCherry‐B56γ_1_ construct into the genome of HeLa Flp‐in cells, followed by puromycin selection. These cells were then used to stably express doxycycline‐inducible and YFP‐tagged KNL1^WT^, KNL1^ΔMELT^, KNL1^6xMELT^, KNL1^12xMELT^, KNL1^19xMELT^, BUBR1^WT^, BUBR1^ΔPLK1^, BUBR1^ΔPP2A(ΔK)^, BUBR1^KARD‐2A^, BUBR1^670A^, BUBR1^KARD‐3A^ or BUBR1^KARD‐2D^ by following the same procedure described above.

### Gene knockdown

For all experiments involving re‐expression in HeLa Flp‐in cells, the endogenous mRNA was knocked down and replaced with a siRNA‐resistant YFP‐tagged mutant. The siRNAs used in this study were as follows: siBUBR1 (5′‐AGAUCCUGGCUAACUGUUC‐3′), siBUB1 (5′‐GAAUGUAAGCGUUCACGAA‐3′) and siGAPDH (control siRNA: 5′‐GUCAACGGAUUUGGUCGUA‐3′). All siRNAs were synthesised with UU overhang (Sigma‐Aldrich) and used at 20 nM final concentration. Double‐stranded interference RNA was used to knockdown endogenous KNL1 (sense: 5′‐GCAUGUAUCUCUUAAGGAAGAUGAA‐3′; antisense: 5′‐UUCAUCUUCCUUAAGAGAUACAUGCAU‐3′) (Integrated DNA technologies) at a final concentration of 20 nM. All siRNAs/dsiRNAs were transfected using Lipofectamine® RNAiMAX Transfection Reagent (Thermo Fisher) according to the manufacturer's instructions. After 16 h of knockdown, cells were arrested with thymidine for 24 h. Doxycycline was used to induce expression of the BUBR1 and KNL1 constructs during and following the thymidine block. Cells were then released from thymidine block into full‐growth media supplemented with doxycycline and, when appropriate, nocodazole for 5–7 h for live imaging or 8.5 h before processing for fixed analysis.

### Immunofluorescence

Cells plated on High Precision 1.5H 12 mm coverslips (Marienfeld) were fixed with 4% paraformaldehyde (PFA) in PBS for 10 min or pre‐extracted with 0.1% Triton X‐100 in PEM (100 mM PIPES, pH 6.8, 1 mM MgCl_2_ and 5 mM EGTA) for 1 min before addition of 4% PFA for 10 min. After fixation, coverslips were washed with PBS and blocked with 3% BSA in PBS + 0.5% Triton X‐100 for 30 min, incubated with primary antibodies overnight at 4°C, washed with PBS and incubated with secondary antibodies plus DAPI (4,6‐ diamidino2‐phenylindole, Thermo Fisher) for an additional 2–4 h at room temperature in the dark. Coverslips were washed with PBS and mounted on glass slides using ProLong antifade reagent (Molecular Probes). All images were acquired on a DeltaVision Core or Elite system equipped with a heated 37°C chamber, with a 100×/1.40 NA U Plan S Apochromat objective using softWoRx software (Applied precision). Images were acquired at 1 × 1 binning using a CoolSNAP HQ or HQ2 camera (Photometrics) and processed using softWorx software and ImageJ (National Institutes of Health). Mitotic cells were selected for imaging based on good expression of YFP at the kinetochore (KNL1) or cytoplasm (BUBR1 cells). All immunofluorescence images displayed are maximum‐intensity projections of deconvolved stacks and were chosen to closely represent the median quantified data. Figure panels were created using Omero (http://openmicroscopy.org).

The following primary antibodies (all diluted in 3% BSA in PBS) were used at the final concentration as indicated: chicken anti‐GFP (ab13970 from Abcam, 1:5,000), rabbit anti‐mCherry (GTX128508 from Genetex, 1:1,000—pre‐extraction required to probe mCherry‐B56γ_1_), guinea pig anti‐CENP‐C (PD030 from Caltag + Medsystems, 1:5,000), rabbit anti‐BUB1 (A300‐373A from Bethyl, 1:1,000), mouse anti‐BUB1 (ab54839 from Abcam, 1:400), mouse anti‐BUBR1 (05‐898 from Millipore, 1:1,000), rabbit anti‐BUBR1 (A300‐386A from Bethyl, 1:1,000), rabbit anti‐PLK1 (IHC‐00071 from Bethyl, 1:1,000), mouse anti‐PLK1 (ab17057 from Abcam, 1:1,000), rabbit anti‐BUBR1‐pT680 (ab200061 from Abcam, 1:1,000), mouse anti‐MAD1 (MABE867 from Millipore, 1:1,000), mouse anti‐HEC1 (ab3613 from Abcam, 1:2,000) and mouse anti‐FLAG(M2) (F3165‐.2MG from Sigma, 1:2,000).

The rabbit anti‐pMELT‐KNL1 antibody is directed against Thr 943 and Thr 1155 of human KNL1 (Nijenhuis *et al*, 2014) (1:1,000—gift from G. Kops, Hubrecht, NL). The rabbit anti‐BUBR1‐pT620 antibody was raised against phosphor‐Thr 620 of human BUBR1 using the peptide C‐AARFVS[pT]PFHE (custom raised by Moravian, 1:1,000, pre‐extraction required) (Cordeiro *et al*, 2020). The rabbit anti‐BUBR1‐pS676 antibody was raised against phosphor‐S676 of human BUBR1 using the following peptide C‐PIIED[pS]REATH (custom made by Biomatik, 1:200). The rabbit anti‐BUBR1‐pS670 antibody was raised against phosphor‐Ser 670 of human BUBR1 (Nijenhuis *et al*, 2014) at 1:1,000. The rabbit anti‐BUB1‐pT461 is directed against phosphor‐Thr 461 of human BUB1 (1:500—gift from M. Bollen, Leuven, BE) (Qian *et al*, 2017). The rabbit anti‐HEC1‐pS55 is directed against phosphor‐Ser 55 of human HEC1 (1:500—gift from J.G. DeLuca, Fort Collins CO). The rabbit anti‐MAD1‐pT716 was raised against phosphor‐Thr 716 of human MAD1 (custom made by Biomatik, 1:1,000) (Allan *et al*, 2020).

Secondary antibodies used were highly cross‐absorbed goat anti‐chicken Alexa Fluor 488 (A‐11039), goat anti‐rabbit Alexa Fluor 568 (A‐11036), goat anti‐mouse Alexa Fluor 488 (A‐11029), goat anti‐mouse Alexa Fluor 568 (A‐11031), goat anti‐guinea pig Alexa Fluor 647 (A‐21450), donkey anti‐rabbit Alexa Fluor 647 (A‐31573) or donkey anti‐mouse Alexa Fluor 647 (A‐31571) all used at 1:1,000 (Thermo Fisher).

### Western blotting

In the western blots shown in Fig 1F and G, cells were arrested with thymidine for 24 h, then released in nocodazole for 16 h to enrich mitotic cells. Doxycycline was used to induce the expression of the BUBR1 constructs during and following the thymidine block. Protein lysates for immunoblot were prepared by harvesting mitotic cells by a mitotic shake‐off, pelleting and washing with cold PBS. After centrifuging samples at 200 g for 3 min, pellets were lysed in ice‐cold RIPA buffer (50 mM Tris pH 8.0, 150 mM NaCL, 1% NP40, 0.5% sodium deoxycholate, 2 mM EDTA pH 8.0, 0.1% SDS, 50 mM NaF and protease inhibitor cocktail) on ice for 20 min. Lysates were centrifuged at 13,000 *g* at 4°C for 10 min, followed by DC Protein Assay (Biorad) to estimate the concentration of each sample. Samples were then mixed with loading buffer to final concentrations of 62.5 mM Tris pH 6.8, 2.5% SDS, 10% glycerol, 5% β‐mercaptoethanol and bromophenol blue. Samples were boiled, then run on 8% SDS–PAGE gels and transferred onto PVDF. Blots were then blocked and incubated overnight at 4°C in primary antibody. Then membranes were washed in TBS with 0.1% Tween 20 (TBS‐T), incubated in HRP‐conjugated secondary antibody (BioRad) for 1 h at RT, washed in TBS‐T and imaged with ECL.

The following primary antibodies (all diluted in 3% BSA in PBS) were used at the final concentration indicated: rabbit anti‐BUBR1 (A300‐386A from Bethyl, 1:1,000 in 5% milk in TBS‐T after blocking in 5% milk in TBS‐T), rabbit anti‐BUBR1‐pT680 (ab200061 from Abcam, 1:1,000 in 5% milk in TBS‐T after blocking in 5% milk in TBS‐T), rabbit anti‐BUBR1‐pT620 (custom raised by Moravian, 1:1,000 in 5% BSA in TBS‐T after blocking in 5% milk in TBS‐T), rabbit anti‐BUBR1‐pS670 (Nijenhuis *et al*, 2014) (1:1,000 in 5% milk in TBS‐T after blocking in 5% milk in TBS‐T) (Suijkerbuijk *et al*, 2012), rabbit anti‐BUBR1‐pS676 (custom raised by Biomatik, and used at 1:750 in ReliaBLOT® Block—Bethyl labs—after blocking in ReliaBLOT® Block, as per manufacturer's instructions) and mouse anti‐α‐tubulin (T5168‐.2ML from Sigma‐Aldrich, 1:5,000).

### dCas9‐based in‐cell protein–protein interaction assay: in‐cell interaction assay to study PLK1 and mCherry‐B56γ recruitment to BUBR1 on a chromatin locus

A dCas9‐DARPin‐based system was established to examine the interaction of YFP‐BUBR1 mutants with PLK1 or PP2A on a repetitive chromosomal locus that is distant from kinetochores (Figs 3D–F and EV3G and H and Appendix Fig S1C and D). We tested recruitment of YFP‐BUBR1 to repeats on chromosomes I, III or XIII using gRNAs (as specified in the cloning section) in HeLa FRT cells. The ChrI gRNA gave the largest proportion of cells with visible foci so that was used in all experiments. Experiments were performed by transfecting YFP‐BUBR1 mCherry‐B56γ_1_ cells with dCas9‐DARPin‐FLAG and pU6‐sgChrI (at 1:3 ratio of dCas9:sgRNA) using Fugene HD (Promega) and according to the manufacturer's instructions. The knockdown of the endogenous BUBR1 was then performed as described above. Doxycycline was added to induce the expression of YFP‐BUBR1 mutants and dCas9‐DARPin‐FLAG during and after a thymidine block, as described above. Cells were then released from thymidine block into full‐growth media supplemented with doxycycline and nocodazole for 8.5 h before processing for fixed analysis. Given the ability of the DARPin sequence to bind GFP derivatives (Brauchle *et al*, 2014), dCas9‐DARPin‐FLAG recruits YFP‐BUBR1 to the chromatin region specified by sgRNAs, allowing the quantification of PLK1 or PP2A co‐recruitment to this ectopic locus. Cells were, therefore, stained for YFP‐BubR1, mCherry‐B56γ and dCas9‐DARPin‐FLAG or PLK1 as described above. Only mitotic cells showing bright YFP‐BUBR1 foci were imaged and quantified.

### SAC strength assays

To measure SAC strength in live cells, cells were incubated in a 24‐well plate in full‐growth media in a heated chamber (37°C and 5% CO_2_) and imaged with brightfield microscopy using a 10×/0.5 NA objective and a Hamamatsu ORCA‐ER camera at 2 × 2 binning on a Zeiss Axiovert 200M, controlled by Micro‐manager software (open source: https://micro‐manager.org/) or a 20×/0.4 NA air objective and a CMOS Orca flash 4.0 camera at 4 × 4 binning on a Zeiss Axio Observer 7. Mitotic exit was defined by cells flattening down in the presence of nocodazole and MPS1 ± PLK1 or BUB1 inhibitors. MPS1 was inhibited with AZ‐3146 shortly prior to imaging, with or without PLK1 inhibition with BI‐2536 or BUB1 inhibition with BAY‐1816032. In Fig EV2G, cells entering mitosis in the presence of nocodazole were analysed. In Figs 2E, 3H, EV3J and 5A, cells entering in mitosis in the presence of AZ‐3146 were analysed. In Figs 5D and EV5C, cells arrested in mitosis at the time of AZ‐3146 ± BI‐2536 or BAY‐1816032 treatment were analysed.

To measure SAC strength in fixed cells, nocodazole and MG132 ± BI‐2536 or BAY‐1816032 were added first for 30 min to ensure complete inhibition, followed by a time course of AZ‐3146 ± BI‐2536 or BAY‐1816032 in media containing nocodazole and MG132. Cells were fixed and analysed by immunofluorescence, probing for KNL1‐pMELT or BUB1.

### Chromosome alignment assays

To observe live chromosome alignment and determine mitotic cell fates and timing in an unperturbed cell cycle, cells were plated in 8‐well or 18‐well chamber slides (ibidi), released from thymidine block for 5–6 h and incubated with SiR‐DNA far‐red DNA probe (1:10,000, Spirochrome; to prevent toxicity; Sen *et al*, 2018) in L‐15 or DMEM (no phenol red) media for 15 min prior to imaging. Images were captured after rinsing off the DNA dye, every 4 min for 16 h with a 40×/1.3 oil immersion objective or 40×/0.95 air objective using a Zeiss Axio Observer 7 with a CMOS Orca flash 4.0 camera at 4 × 4 binning and 10 z‐stacks with a step size of 1.50 μm. Cells were selected for quantification based on good expression of YFP‐tagged protein. Selected cells were scored based on the following mitotic events: cohesion fatigue, cell division or cell death following chromosome alignment or not. Dividing cells were also scored based on the type of chromosome segregation defect (no visible defects, anaphase bridges or lagging chromosomes).

To observe chromosome alignment in fixed‐cell experiments—with the advantage of taking high‐resolution images and easily selecting many cells for further analysis—cells were released from thymidine block for 7 h before being treated for 2 h with RO‐3306—to synchronise cells at the G2/M boundary—or with a 3‐h treatment with STLC—to arrest cells in mitosis with monopolar spindles. Cells treated with RO‐3306 were then washed three times and incubated for 15 min with full‐growth media before addition of MG132 to prevent mitotic exit, fixing cells 30′ after the addition of MG132. Cells treated with STLC were then washed three times and incubated in full‐growth media supplemented with MG132, fixing cells every 15′ from 45′ to 105′ after the addition of MG132. Fixed cells were stained as described above and imaged on a Zeiss Axio Observer with a CMOS Orca flash 4.0 camera at 4 × 4 binning, using a Plan‐apochromat 20×/0.4 air objective. Cells with a good expression of YFP‐tagged protein were scored based on the number of misaligned chromosomes as aligned (0 misaligned chromosomes, with a visible metaphase plate), mild (1–2), moderate (3–5), severe (> 6) or no visible alignment (for cells released from STLC in which a clear metaphase plate was not visible—either because of a monopolar spindle induced by the STLC treatment or because the metaphase plate was rotated). This protocol is important because mutants that cause a prolonged arrest can otherwise cause cohesion fatigue, which skews the alignment data.

To observe chromosome segregation defects in fixed‐cell experiments, cells were treated with STLC as described above but then released in MG132‐free media to allow cells to proceed into anaphase. Cells were fixed 90′ after the STLC washout and imaged as described above. Anaphase cells with a good expression of YFP‐tagged protein were scored based on the type of chromosome segregation defect (no visible defects, anaphase bridges, lagging chromosomes or micronuclei).

To measure the proportion of unstable KT‐MT attachments, cells were treated with RO‐3306 as described above in chromosome alignment experiments. After washout from RO‐3306 and a 30‐min treatment with MG132, the media were replaced with ice‐cold L‐15 supplemented with MG132 and cells were incubated in ice to induce cold shock. Cells were then fixed 0, 10 and 20 min after the temperature shift, and stained for MAD1 (i.e. marker of unattached kinetochores). The number of kinetochores unattached to the mitotic spindle was estimated by counting the number of kinetochores positive for MAD1 recruitment.

### Comparison of kinetochore protein levels in prometaphase versus metaphase

To compare kinetochore protein levels in cells enriched in prometaphase versus metaphase (Figs 6A and E, and 7C and Appendix Fig S4A), cells were treated with RO‐3306 as described above in chromosome alignment experiments. After the RO‐3306 washout, cells were treated with nocodazole or MG132 for 30′, to enrich cells in prometaphase and metaphase, respectively. Cells were then fixed and stained as described above.

### Comparison of kinetochore protein levels in metaphase cells treated with PLK1/MPS1 inhibitors

To compare kinetochore protein levels in metaphase cells in the presence of PLK1/MPS1 inhibition (Fig 6C), cells were treated with RO‐3306 and released into MG132 for 30′ as described above. Cells were then treated with DMSO, BI‐2536 (PLK1i) and/or AZ‐3146 (MPS1i) for 30′. Cells were then fixed and stained as described above.

### Comparison of BUB1 and KNL1 recruitment to the midbody in anaphase cells treated with PLK1 inhibitor

To compare the recruitment of proteins to the midbody in anaphase cells treated or not with PLK1 inhibitor (Fig 6F–H), cells were treated with STLC for 3 h and released into full‐growth media as described above. After 75′ from the release, cells were treated with DMSO or BI‐2536 for 15′. Cells were then fixed and stained as described above. Cells were scored based on the presence of a high/low/no visible signal of KNL1 and BUB1 in the midbody.

### FRAP measurements

YFP‐KNL1^WT^, YFP‐KNL1^6xMELT^ and YFP‐KNL1^19xMELT^ cells were transiently transfected with pcDNA5‐FLAG‐tagRFP‐BUB1^WT^ or pcDNA5‐FLAG‐tagRFP‐BUBR1^WT^ plasmids, using Fugene HD (Promega) and according to the manufacturer's instructions. Cells were then treated as described previously, to allow the expression of the YFP‐tagged KNL1 and of the tagRFP‐tagged BUB1/BUBR1, at the same time as knocking down the endogenous KNL1 and BUB1/BUBR1. Cells were released from the thymidine block in nocodazole for 16 h to enrich mitotic cells. Cells were then imaged in L‐15 media, using a DeltaVision Elite system equipped with a heated 37°C chamber, with a 60×/1.42 NA U Plan S Apochromat objective using softWoRx software (Applied precision). For each cell, an individual kinetochore was positioned close to the centre of the field of view and then bleached with an A488 laser (power 100%, 0.05 s). Images were acquired at 2 × 2 binning using a CoolSNAP HQ camera (Photometrics), taking images (i) every 1 s from −2 s to 30 s with respect to the bleaching event and (ii) every 2 s from 30 s to 90 s after the bleaching. To monitor YFP‐KNL1 and tagRFP‐BUB1/BUBR1 levels in each individual bleached cell, a high‐speed filter set was used to acquire images on YFP and mCherry channels during the experiment. Movies of individual cells were then processed for FRAP measurements with a custom pipeline based on MATLAB® scripts. The pipeline is based on the following steps:

- Circular regions around the bleached kinetochore (KT), in the unbleached portion of the cytoplasm (CYTO) and in the extracellular environment (BKG) were defined by the user. The script makes sure these regions have the same area. The integrated fluorescence intensities of these areas (F_KT_, F_CYTO_ and F_BKG_ respectively) were then measured for the entire movie, allowing the user to modify the position of the KT area in case of any movement of the kinetochore during the recovery after photobleaching.
- The background intensity was removed from KT and CYTO values, resulting in the normalised $F_{\mathrm{KT}}^{\prime }$ = F_KT_ − F_BKG_ and $F_{\text{CYTO}}^{\prime }$ = F_CYTO_ − F_BKG_ respectively.
- $F_{\mathrm{KT}}^{\prime }$ and $F_{\text{CYTO}}^{\prime }$ were then normalised to the pre‐bleach fluorescence, which was evaluated as the average of the fluorescence intensities among the frames before the bleaching event. This step produced the normalised values I_KT_ and I_CYTO_.
- I_KT_ was then normalised for I_CYTO_, to correct for photofading coming from the continuous imaging after photobleaching (Koulouras *et al*, 2018). This gave
$$I_{\mathrm{KT}}^{\text{NORM}}=\frac{I_{\mathrm{KT}}}{I_{\text{CYTO}}}.$$
- The normalised integrated fluorescence intensity of the bleached kinetochore was then evaluated with the following formula:
$$\mathrm{FRA}P_{\mathrm{KT}}=\frac{I_{\mathrm{KT}}^{\text{NORM}}-I_{\mathrm{KT}}^{\text{NORM}}\left(t_{\text{BLEACH}}\right)}{1-I_{\mathrm{KT}}^{\text{NORM}}\left(t_{\text{BLEACH}}\right)},$$

where $I_{\mathrm{KT}}^{\text{NORM}}\left(t_{\text{BLEACH}}\right)$ represents the value of $I_{\mathrm{KT}}^{\text{NORM}}$ at the time of the bleaching event. The formula above makes sure the bleaching depth is normalised on the interval [0, 1] (Koulouras *et al*, 2018).

Trajectories from individual cells were then checked and discarded if they were too noisy or showed a decay in intensity after photobleaching. The average of the remaining trajectories was then evaluated for each experimental condition. Each average trajectory was fitted with a double‐exponential law $F\left(t\right)=A\left(1-e^{‐\mathrm{ht}}\right)+B\left(1-e^{-\mathrm{kt}}\right),$ in which (i) the mobile fraction is represented by A+B, (ii) the half‐life of the fast‐recovery phase by $\min\left\{\frac{\ln2}{h},\frac{\ln2}{k}\right\}$ and (iii) the half‐life of the slow‐recovery phase by $\max\left\{\frac{\ln2}{h},\frac{\ln2}{k}\right\}$. The adjusted *R* ^2^ index was used as a measure of goodness of fit.

### Image quantification and statistical analysis

For quantification of kinetochore protein levels, images of similarly stained experiments were acquired with identical illumination settings and analysed using an ImageJ macro, as described previously (Saurin *et al*, 2011). Fluorescence intensities at kinetochores were normalised to CenpC (i.e. kinetochore marker), with the exception of HEC1‐pS55 intensities—which were normalised to Hec1. Measurements of phosphorylated BUBR1 were normalised to those of YFP‐BUBR1 to avoid artificial fluctuation of signal resulting from variability in re‐expression levels of YFP‐BUBR1. The same principle was adopted in YFP‐KNL1 cells to measure levels of BUB1, BUB1‐pT461, BUBR1, KNL1‐pMELT, PLK1 and mCherry‐B56γ.

For quantification of protein levels on Chr I foci with the dCas9‐DARPin strategy (Figs 3E and F, and EV3G and H and Appendix Fig S1D), images of similarly stained experiments were acquired with identical illumination settings and analysed using ImageJ. An ImageJ macro was designed to threshold and select the Chr I foci automatically (either by performing a “Default” threshold on dCas9‐DARPin‐FLAG signal or a “Intermodes” threshold on YFP‐BUBR1 signal) inside the chromatin area (using DAPI channel). The threshold selection was increased by 1 pixel (to ensure complete Chr I foci selection). These selections were then used to calculate the mean foci intensity of PLK1 or mCherry‐B56γ relative to YFP‐BUBR1. A background outside the DAPI area was used to subtract from the foci intensity.

Violin plots were produced using PlotsOfData—https://huygens.science.uva.nl/PlotsOfData/ (Postma & Goedhart, 2019). This allows the spread of data to be accurately visualised along with the 95% confidence intervals (thick vertical bars) calculated around the median (thin horizontal lines). This representation allows the statistical comparison between all treatments and time points because when the vertical bar of one condition does not overlap with one in another condition, the difference between the medians is statistically significant (*P* < 0.05). All the other plots were generated with MATLAB® or GraphPad Prism 7.

### Analysis of PLK1‐ and PP2A‐binding motifs in BUBR1 throughout metazoa

The dataset from Tromer *et al* (2016a) (Data ref: Tromer *et al*, 2016b) was used and annotated for the presence of PLK1‐ and PP2A‐binding sites in metazoa, as described previously (Cordeiro *et al*, 2020). Sequence alignments were generated using Jalview (Waterhouse *et al*, 2009). The consensus sequence for MELT motifs was created using WebLogo (Crooks *et al*, 2004; https://weblogo.berkeley.edu/logo.cgi).

### Estimation of the number of KNL1 molecules and the active MELT motifs per kinetochore

To estimate the number of KNL1 molecules per kinetochore in our set of KNL1 MELT mutants (Fig EV4G and H), we assumed the presence of 151 KNL1 molecules per kinetochore in HeLa cells (Suzuki *et al*, 2015). This number was then scaled by using the median kinetochore levels of YFP‐KNL1 in our set of KNL1 MELT mutants (see Fig EV4A and I). In other words, for a generic KNL1 MELT mutant *Nx* (with *N* active MELTs), the number of the KNL1 molecules per kinetochore is given by

$${\mathrm{KNL}1}_{\text{molecues}}^{\mathrm{Nx}}=151\;\text{molecules}\times \frac{{\text{levels}}^{\mathrm{Nx}}}{{\text{levels}}^{\mathrm{WT}\;\mathrm{Knl}1}}=\frac{151\;\text{molecules}\times {\text{levels}}^{\mathrm{Nx}}}{0.9692}$$

To get an estimate of the number of active MELTs per kinetochore, we then multiplied the number of KNL1 molecules per kinetochore by the number of active MELTs per KNL1 molecule. In other words, for a generic KNL1 MELT mutant *Nx* (with *N* active MELTs), the number of the active MELTs per kinetochore is given by:

$${\text{MELTs}}_{\text{molecules}}^{\mathrm{Nx}}=N\times {\mathrm{KNL}1}_{\text{molecues}}^{\mathrm{Nx}}$$

In the case of YFP‐KNL1 WT cells, we used the assumption that WT KNL1 has seven active MELTs (Vleugel *et al*, 2015). The estimated numbers of the active MELTs per kinetochore (${\text{MELTs}}_{\text{molecules}}^{\mathrm{Nx}}$) were plotted against the numbers of active MELTs per KNL1 molecule (*N*) and fitted with an exponential plateau law $Y=Y_{M}‐\left(Y_{M}‐Y_{0}\right)e^{‐kx}$ with GraphPad Prism 7. The fit was performed by constraining Y_0_ = 0 and excluding the data of WT KNL1 cells from the fit. Y_M_ was considered as an estimation of the plateau, and adjusted *R* ^2^ as a measure of goodness of fit.

## Author contributions

**Andrea Corno:** Conceptualization; data curation; software; formal analysis; validation; investigation; visualization; methodology; writing – original draft. **Marilia H Cordeiro:** Conceptualization; data curation; formal analysis; validation; investigation; visualization; methodology; writing – review and editing. **Lindsey A Allan:** Validation; investigation; visualization. **Qian Wei:** Investigation. **Elena Harrington:** Investigation. **Richard J Smith:** Investigation. **Adrian T Saurin:** Conceptualization; data curation; supervision; funding acquisition; visualization; methodology; writing – original draft; project administration.

## Disclosure and competing interests statement

The authors declare that they have no conflict of interest.

## Supporting information



Appendix

Expanded View Figures PDF

Movie EV1

Movie EV2

Movie EV3

PDF+

## Data Availability

This study includes no data deposited in external repositories.
