# Supplementary material for: A bifunctional kinase–phosphatase module balances mitotic checkpoint strength and kinetochore–microtubule attachment stability
Source: EMBO J. 2023 Sep 15;42(20):e112630. doi: 10.15252/embj.2022112630 (PMC10577578; doi:10.15252/embj.2022112630)
Supplement: Supplementary file 1 — Appendix [file EMBJ-42-e112630-s007.pdf]

APPENDIX

A bifunctional kinase-phosphatase module balances mitotic checkpoint strength and kinetochore-microtubule attachment stability

Andrea Corno, Marilia H Cordeiro, Lindsey A Allan, Qian Wei, Elena Harrington, Richard J Smith, and Adrian T. Saurin.
Division of Cellular and Systems Medicine, School of Medicine, University of Dundee, UK. DD1 9SY;
Correspondence: a.saurin@dundee.ac.uk

Table of Content

Appendix Figure S1 (related to Figure 3) - Details of BUBR1 alanine and aspartate mutants 2

Appendix Figure S2 (related to Figure 4) - Details on KNL1 MELT motifs and the KNL1 MELT mutants 3

Appendix Figure S3 (related to Figure 4) - Details of the FRAP measurements of BUB1, BUBR1 and KNL1 in KNL1 MELT mutants 4

Appendix Figure S4 (related to Figure 6) - Details on BUB1 and BUBR1 localisation in metaphase and anaphase cells expressing the KNL1-MELT mutants 5

Appendix Figure S5 (related to Figure 7) - Details on the mitotic cell fate after nuclear envelope breakdown (NEBD) and chromosome alignment in cells expressing the KNL1-MELT mutants 6

Appendix Figure S1 (related to Figure 3)

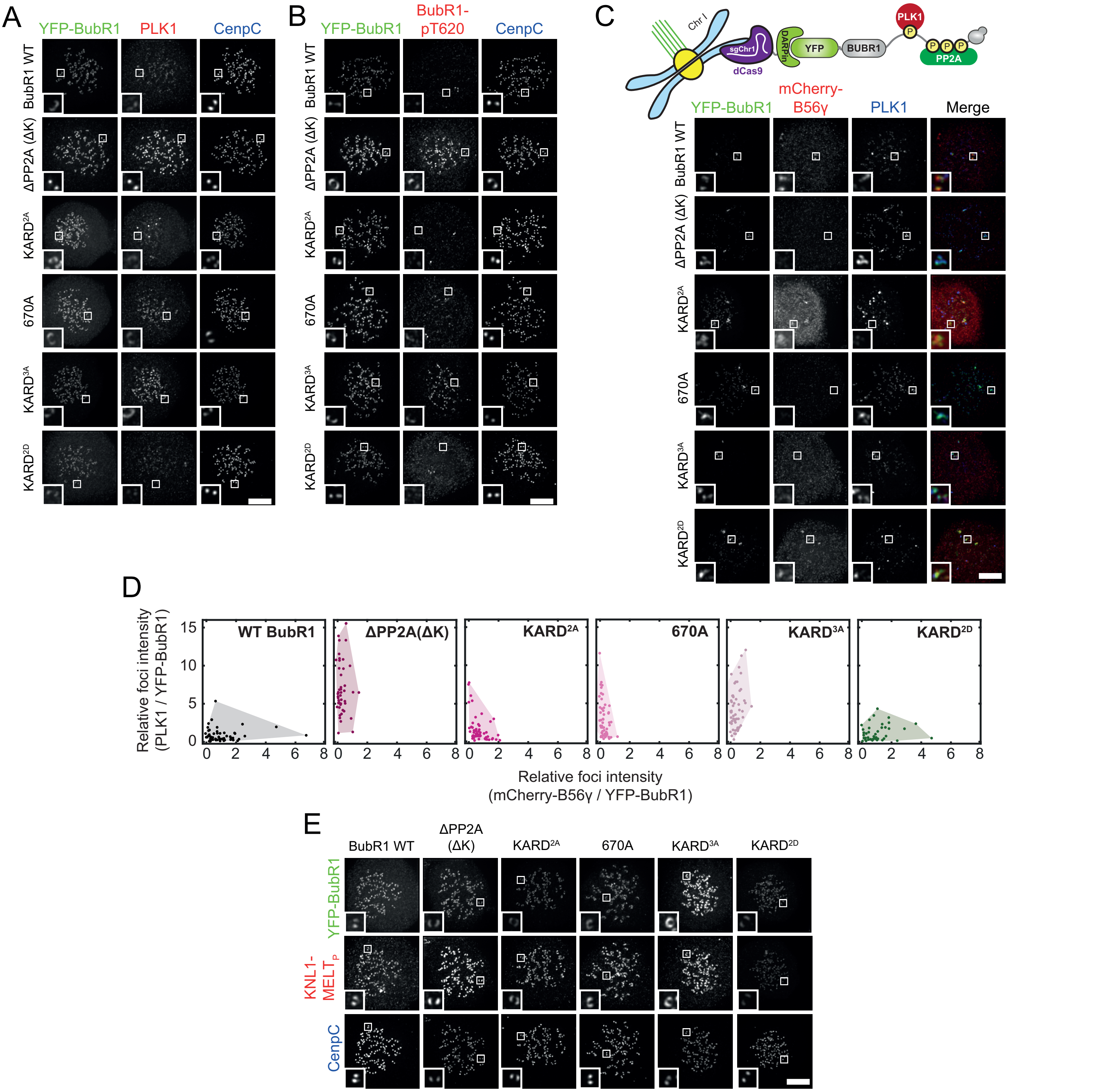

Appendix Figure S1 (related to Figure 3) – Details of BUBR1 alanine and aspartate mutants.

(A-C) Example immunofluorescence images from the quantifications shown in Figures 3B-F and EV3E-H. The insets show magnifications of the outlined regions. Scale bars: 5 μm. Inset size: 1.5 μm.

(D) Correlation between PLK1 and mCherry-B56γ levels per YFP-BUBR1 on Chr I foci shown in Figures 3E-F and EV3G-H. Individual points correspond to measures from individual cells. A convex hull including all the data points is represented per each BUBR1 mutant. Foci intensities from 43-55 cells, 3 experiments.

(E) Example immunofluorescence images from the quantifications shown in Figures 3G and EV3I. The insets show magnifications of the outlined regions. Scale bars: 5 μm. Inset size: 1.5 μm.

Appendix Figure S2 (related to Figure 4)

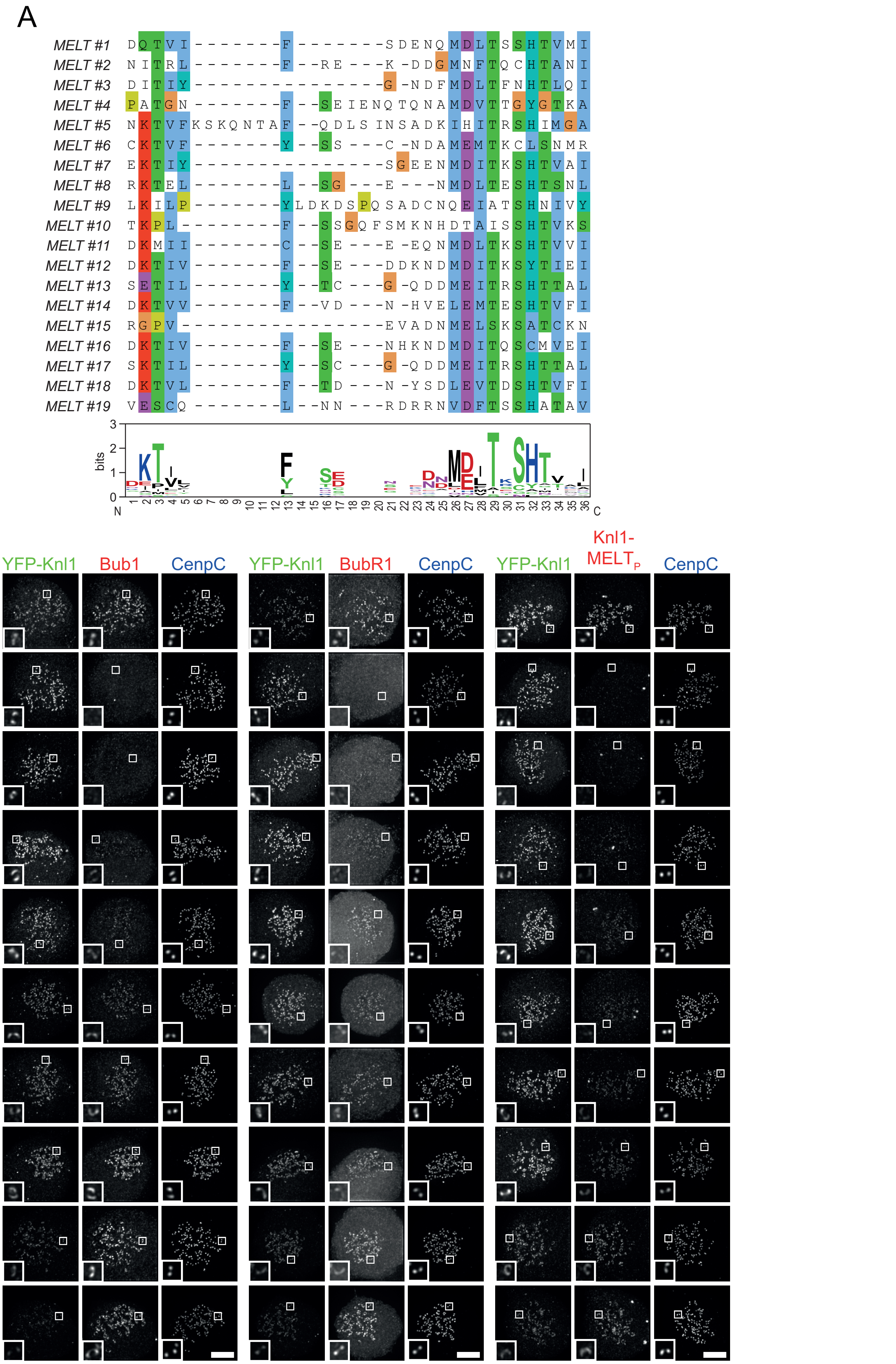

Appendix Figure S2 (related to Figure 4) – Details on KNL1 MELT motifs and the KNL1 MELT mutants.

(A) Alignment of the 19 MELT motifs present in human KNL1. The weblogo represents the consensus sequence shared among all the motifs.

(B) Example immunofluorescence images from the quantifications shown in Figure 4C. The insets show magnifications of the outlined regions. Scale bars: 5 µm. Inset size: 1.5 µm.

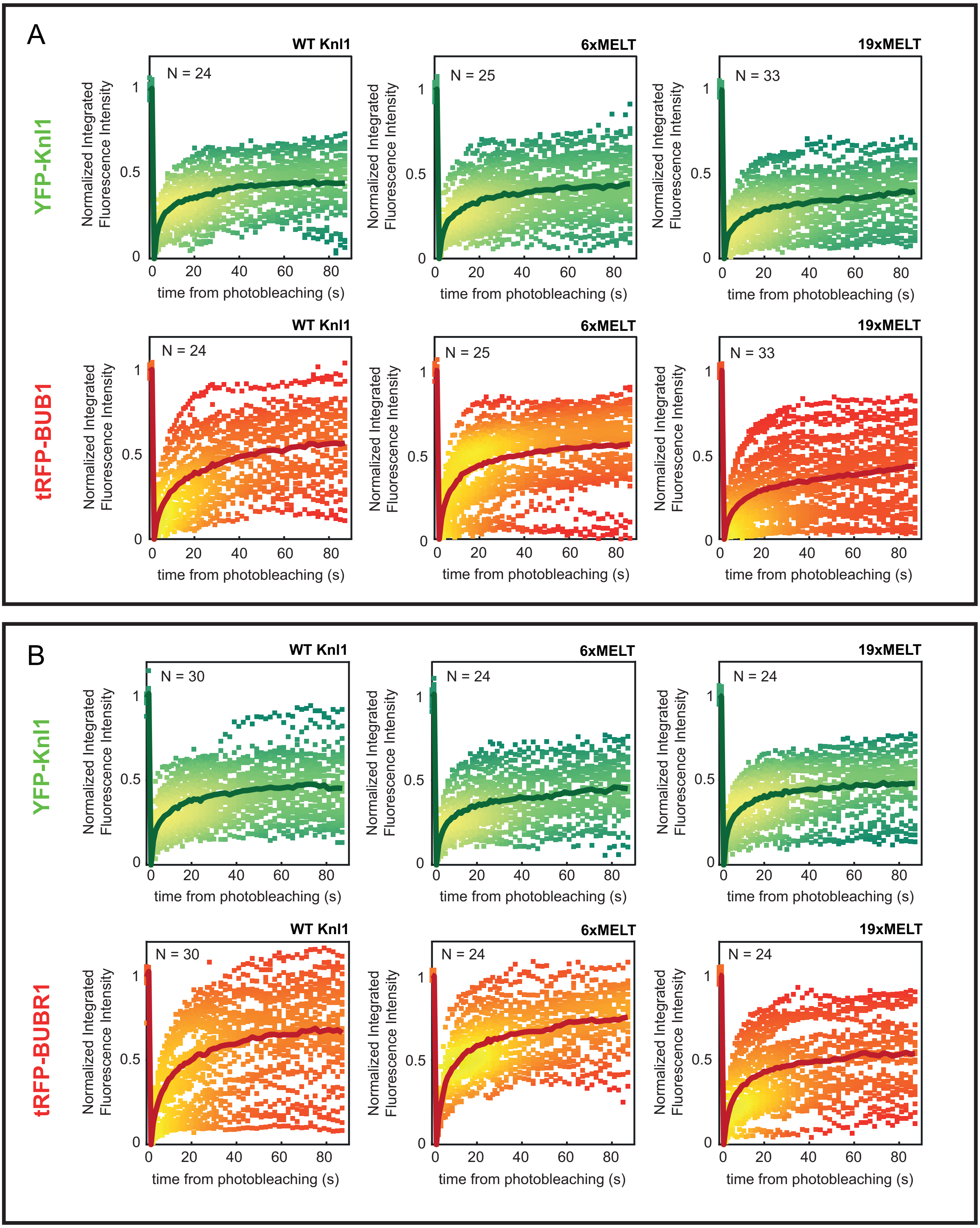

Appendix Figure S3 (related to Figure 4) – Details of the FRAP measurements of BUB1, BUBR1 and KNL1 in KNL1 MELT mutants.

Individual FRAP measurements related to the data shown in Figure EV4D-F (tagRFP-BUB1, tagRFP-BUBR1 and YFP-KNL1). For each condition, each individual point corresponds to a measurement of a single cell at a specific timepoint, while the thick line represents the mean trajectory among cells over time – which is the same shown in Figures EV4D-E. The colour of the data points reflects the density of the data cloud – yellow where the data are more dense, dark red/green where the data are sparser. N represents the number of cells included in the analysis, from 3 experiments.

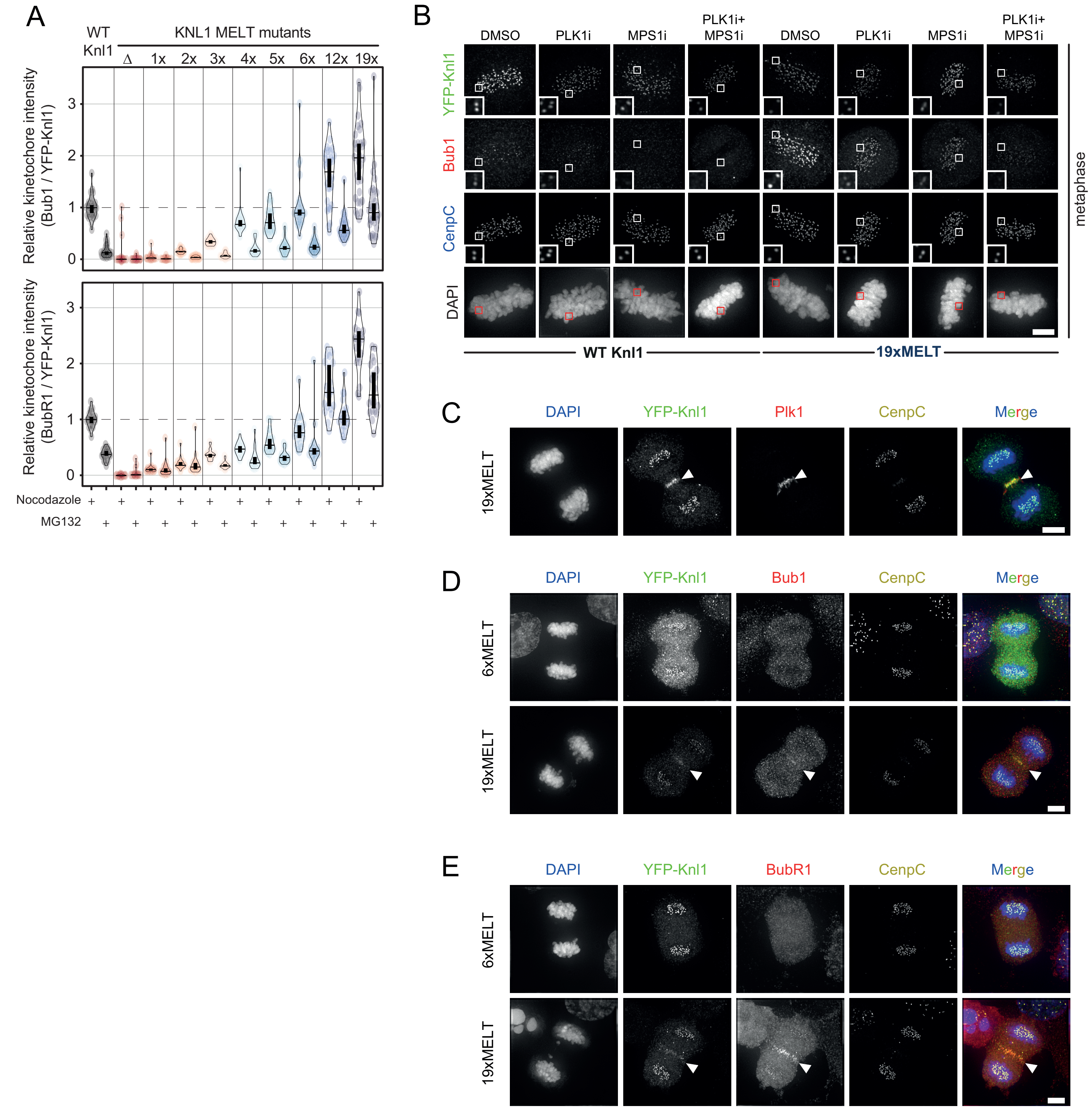

Appendix Figure S4 (related to Figure 6) – Details on BUB1 and BUBR1 localisation in metaphase and anaphase cells expressing the KNL1-MELT mutants.

(A) Levels of BUB1 (top panel) and BUBR1 (bottom panel) at kinetochores, in nocodazole or MG132-treated HeLa FRT cells expressing the indicated KNL1 MELT mutants. Kinetochore intensities from 10-30 cells, 2-3 experiments. Violin plots show the distributions of kinetochore intensities between cells. For each violin plot, each dot represents an individual cell, the horizontal line represents the median, while the vertical one the 95% CI of the median, which can be used for statistical comparison of different conditions (see Materials and Methods). The distributions of MG132-treated cells and of WT Knl1 nocodazole-treated cells are the same shown in Figure 6A.

(B) Example immunofluorescence images from the quantifications shown in Figure 6C. The insets show magnifications of the outlined regions. Scale bars: 5 µm. Inset size: 1.5 µm.

(C-E) Example immunofluorescence images of 19xMELT KNL1 cells recruiting KNL1, PLK1 (C), BUB1 (D) and BUBR1 (E) at the midbody in anaphase cells. White arrows highlight the midbody. Scale bars: 5 µm

A

Same data as Figure 7A but showing the individual repeats

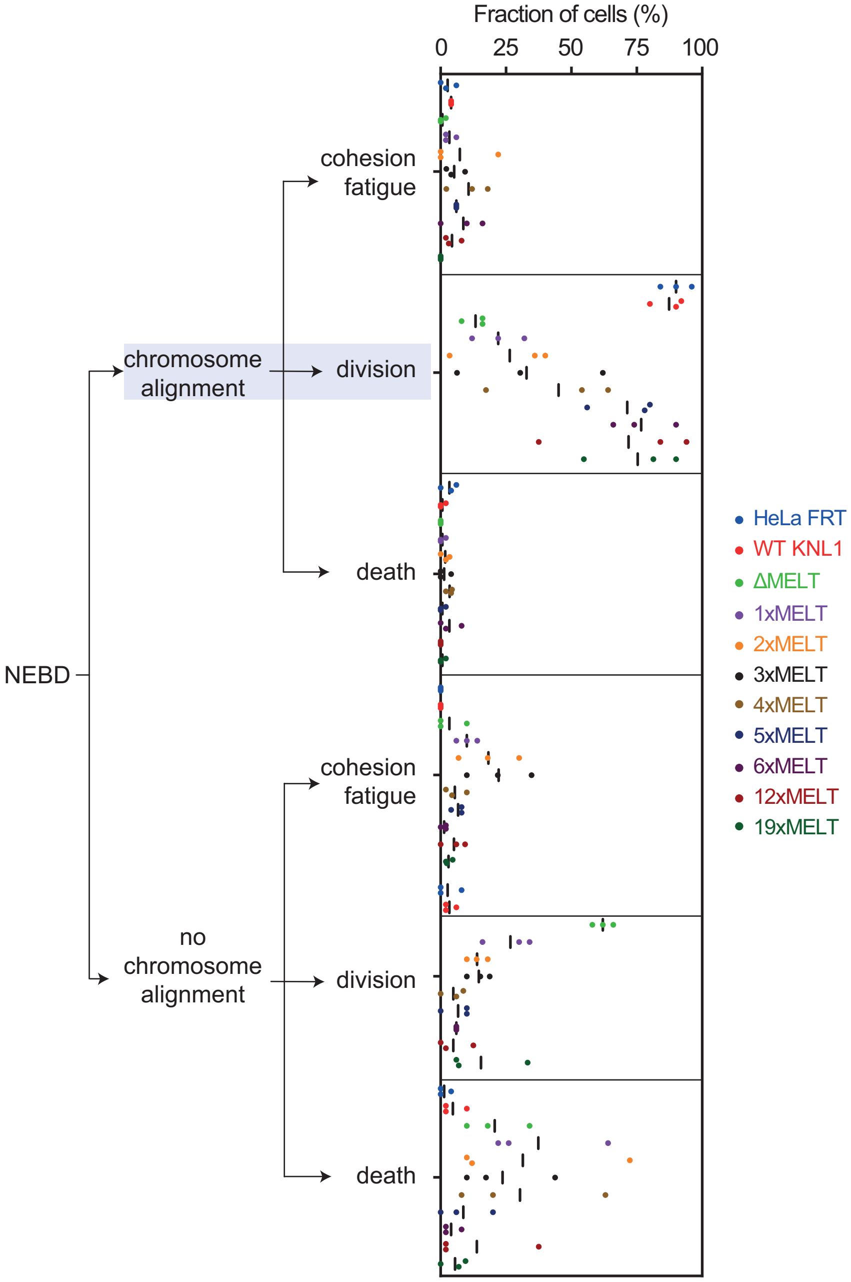

B

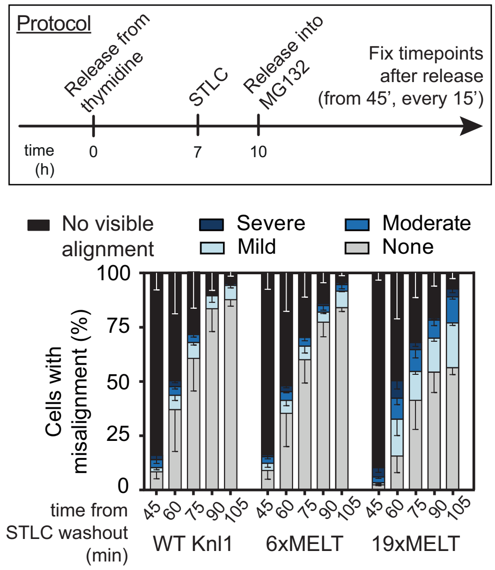

Appendix Figure S5 (related to Figure 7) – Details on the mitotic cell fate after nuclear envelope breakdown (NEBD) and chromosome alignment in cells expressing the KNL1-MELT mutants.

(A) Frequencies of the cell fates shown in Figure 7A from the 3 repeats of the experiment. For each distribution, the thick line corresponds to the mean values reported in Figure 7A.

(B) Evaluating the effects on chromosome alignment in HeLa FRT cells, expressing the indicated KNL1-MELT mutants and challenged to correct more KT-MT attachment errors (using STLC 10µM, see Material and Methods for details). Top panel: protocol used to visualise chromosome alignment in fixed samples (see Material and Methods for details). Bottom panel: mean frequencies of chromosome alignment errors (+/- SEM) from 3 experiments, 100 cells quantified per condition per experiment. Treatment with MG132 (10 µM) was included to prevent mitotic exit. The frequencies at 105’ timepoint are the same shown in Figure 7D.
