# Supplementary material for: A bifunctional kinase–phosphatase module balances mitotic checkpoint strength and kinetochore–microtubule attachment stability
Source: EMBO J. 2023 Sep 15;42(20):e112630. doi: 10.15252/embj.2022112630 (PMC10577578; doi:10.15252/embj.2022112630)
Supplement: Supplementary file 2 — Expanded View Figures PDF [file EMBJ-42-e112630-s003.pdf]

Expanded View Figures

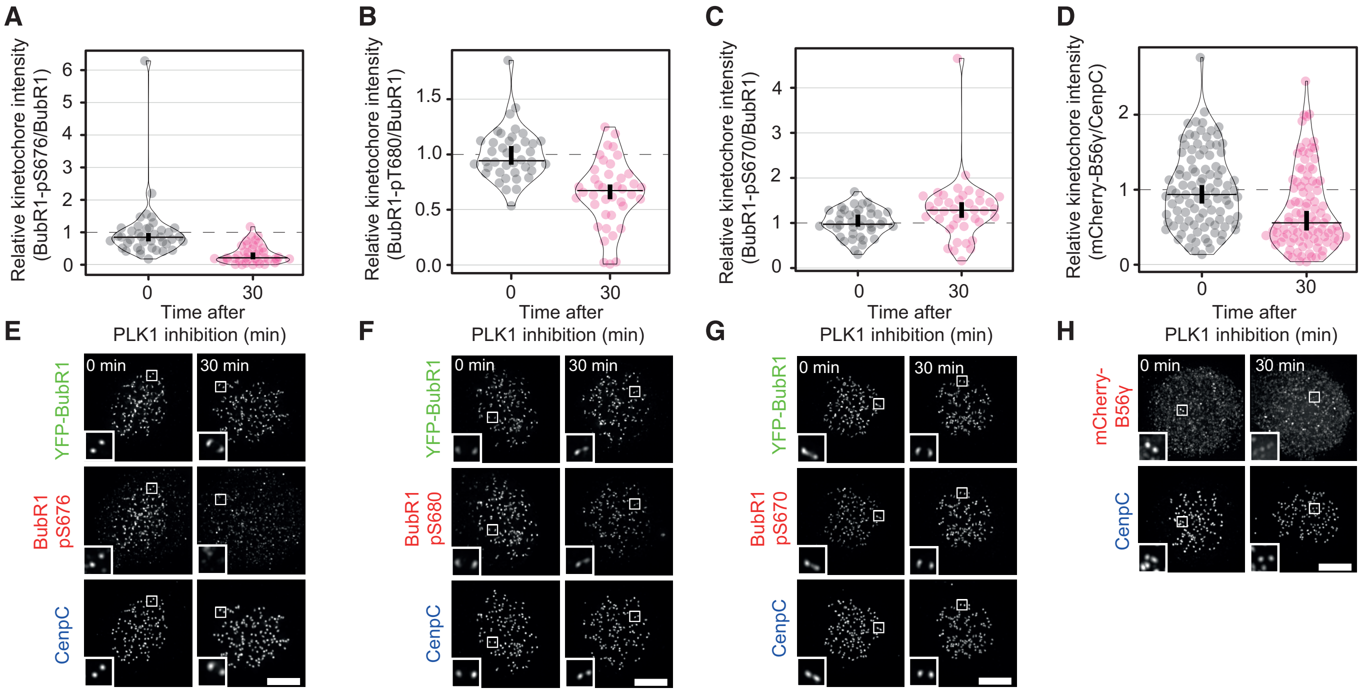

Figure EV1. (Related to Fig 1). Effect of PLK1 inhibition on PP2A-B56 recruitment sites and kinetochore localisation.

A–D Effects of PLK1 inhibition on levels of BUBR1-pS676 (A), BUBR1-pT680 (B), BUBR1-pS670 (C) and mCherry-B56γ (D) at unattached kinetochores, in nocodazole-arrested HeLa FRT cells untreated or treated with the PLK1 inhibitor BI-2536 (100 nM). Kinetochore intensities from 40 to 100 cells, 4–5 experiments. Kinetochore intensities are normalised to the time point 0′. Violin plots show the distributions of kinetochore intensities. For each violin plot, each dot represents an individual cell, the horizontal line represents the median and the vertical one the 95% CI of the median, which can be used for statistical comparison of different conditions (see Materials and Methods).

E–H Example immunofluorescence images of the kinetochore quantifications shown in (A–D). The insets show magnifications of the outlined regions. Scale bars: 5 μm. Inset size: 1.5 μm.

Figure EV2. (Related to Fig 2). Molecular and phenotypic effects of locking PLK1 or PP2A on BUBR1.

A–E Example immunofluorescence images of the kinetochore quantifications are shown in Fig 2B–D. Panel (D) shows the knockdown efficiency of BUB1, in relation to Fig 2B. The insets show magnifications of the outlined regions. Scale bars: 5 μm. Inset size: 1.5 μm.

F Effects of locking PLK1 or PP2A on the levels of PLK1 at unattached kinetochores, in nocodazole-arrested HeLa FRT cells expressing the indicated BUBR1 mutants and treated with the MPS1 inhibitor AZ-3146 (2.5 μM). Treatment with MG132 (10 μM) was included to prevent mitotic exit after the addition of the MPS1 inhibitor. Kinetochore intensities from 30 cells, in three experiments.

G Effects of locking PLK1 or PP2A on the duration of the mitotic arrest in nocodazole-arrested HeLa FRT cells expressing the indicated BUBR1 mutants. Top panel: graph showing mean frequencies (± SEM) of cells that exit from mitosis. Bottom panel: distributions of the mitotic durations. Data from three experiments, 50 cells per condition per experiment.

H Effects of locking PLK1 or PP2A on the levels of HEC1-pS55 at unattached kinetochores, in nocodazole-arrested HeLa FRT cells expressing the indicated BUBR1 mutants. Kinetochore intensities from 45 cells, in three experiments.

I–K Effects of locking PLK1 or PP2A on the mitotic cell fate after nuclear envelope breakdown (NEBD). Panel (I) shows example images from live movies highlighting the most frequent mitotic cell fates. Cell fates are reported in yellow, and white arrows highlight defects during chromosome alignment or segregation. Scale bar: 20 μm. In (J), Left panel: heatmap showing the mean frequencies of cell fates after NEBD for each BUBR1 mutant—three experiments, 50 cells per condition per experiment. Right panel: percentages of the cell fates shown in the left panel from the three repeats of the experiment. For each distribution, the thick line corresponds to the mean values reported in the left panel. Panel (K) displays duration of mitosis (left panel) and prometaphase (right panel) of cells from (I) that divide after NEBD. Sample sizes: 146 cells in BUBR1 WT, 27 cells in ΔPP2A (ΔC) and 150 cells in B56γ. Data from three experiments.

Data information: Kinetochore intensities are normalised to BUBR1 WT control at time point 0′ (F) or to BUBR1 ΔPP2A(ΔC) (H). Violin plots show the distributions of kinetochore intensities (F and H) or the distributions of the mitotic and prometaphase durations (G and K). For each violin plot, each dot represents an individual cell, the horizontal line represents the median and the vertical one the 95% CI of the median, which can be used for statistical comparison of different conditions (see Materials and Methods).

© 2023 The Authors

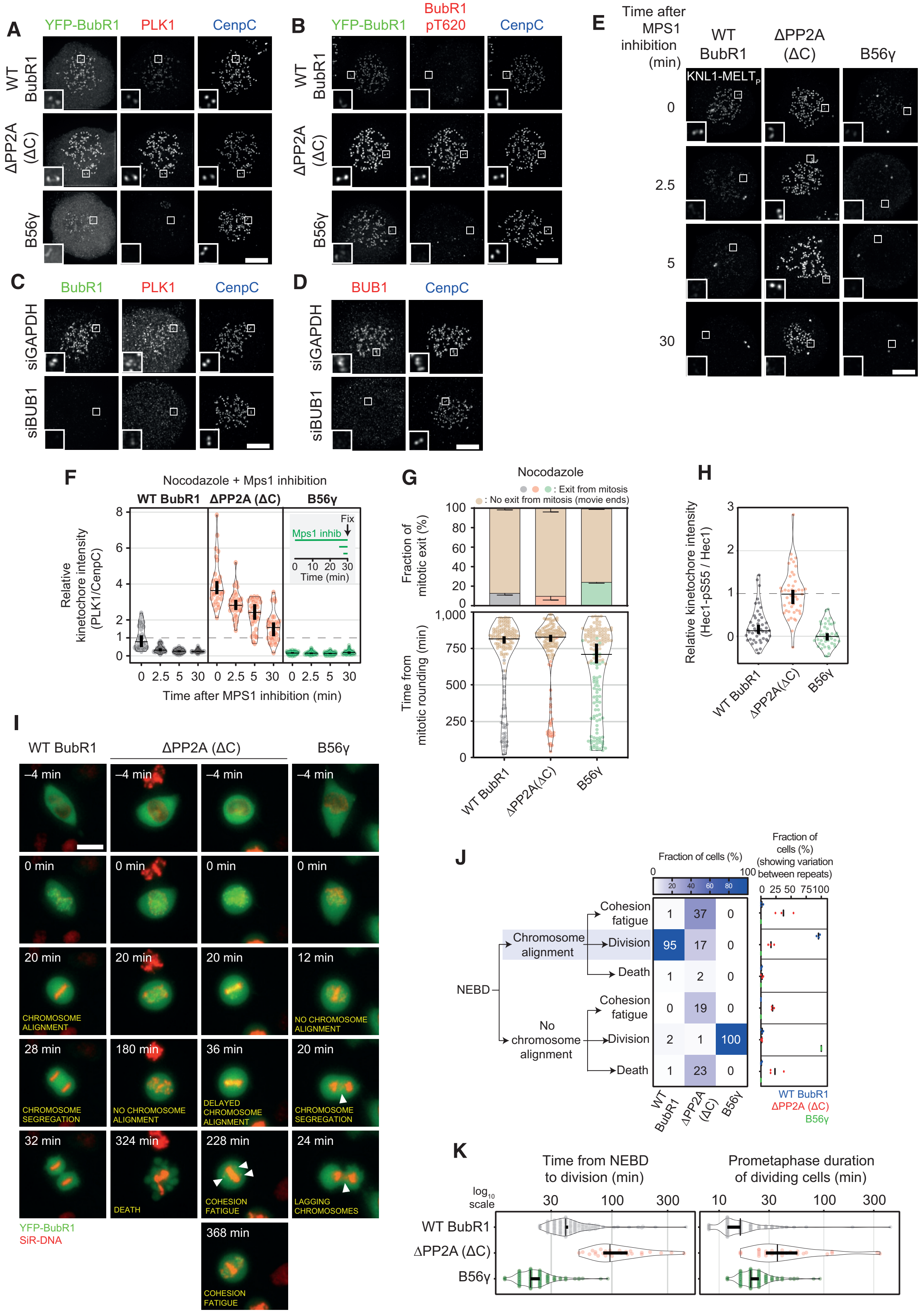

Figure EV2.

 © 2023 The Authors

Figure EV3. (Related to Fig 3). Molecular and phenotypic effects of BUBR1 mutants designed to increase or decrease PP2A levels.

A–C Evaluating the effect of indicated BUBR1 mutants on the mitotic cell fate after nuclear envelope breakdown (NEBD) (A and B) and on the duration of mitosis (C). The heatmap in panel (A) shows the mean frequencies of cell fates after NEBD in each condition—3–6 experiments, 50 cells per condition per experiment. Panel (B) shows the frequencies of the cell fates shown in (A) from the 3 to 6 repeats of the experiment. For each distribution, the thick line corresponds to the mean values reported in (A). Panel (C) shows the duration of mitosis (top panel) and prometaphase (bottom panel) of cells from (A) that divide after NEBD. Sample sizes: 295 cells in BUBR1 WT, 36 cells in ΔPP2A (ΔK), 127 cells in KARD^{2A}, 89 cells in 670A, 93 cells in KARD^{3A} and 135 cells in KARD^{2D}. Data from 3 to 6 experiments.

D Schematic illustrating the PLK1/PP2A feedback loop on WT BUBR1 (left panel) and the BUBR1 aspartate mutant designed to enhance PP2A recruitment (right panel).

E, F Effect of BUBR1 aspartate mutant on levels of BUBR1-pT620 (E) and PLK1 (F) at unattached kinetochores in nocodazole-arrested HeLa FRT cells expressing the indicated BUBR1 mutants. Kinetochore intensities from 30 to 60 cells, 3–6 experiments. Note that distributions of WT BUBR1 condition are the same shown in Fig 3B and C.

G, H Effect of BUBR1 aspartate mutant on levels of PLK1 (G) and mCherry-B56γ (H) at ectopic foci on Chr I, in nocodazole-arrested HeLa FRT cells expressing the indicated BUBR1 mutants (see also Appendix Fig S1C and D and Materials and Methods for details). Foci intensities from 43 to 55 cells, three experiments. Note that distributions of WT BUBR1 condition are the same as shown in Fig 3E and F.

I, J Effect of BUBR1 aspartate mutant on KNL1-MELT phosphorylation (I) and duration of mitotic arrest (J) in nocodazole-arrested HeLa FRT cells expressing the indicated BUBR1 mutants. Cells in panel (J) were treated with the MPS1 inhibitor AZ-3146 (2.5 μM). Panel (I) displays kinetochore intensities of 60–70 cells per condition, six experiments. Panel (J) displays 50 cells per condition per experiment, three experiments. Note that distributions of WT BUBR1 condition are the same as shown in Fig 3G and H.

K–M Effect of BUBR1 aspartate mutant on kinetochore levels of HEC1-pS55 (K), chromosome alignment (L) and stability of kinetochore–microtubule attachments (M). Panel (K) shows levels of HEC1-pS55 at unattached kinetochores, in nocodazole-arrested HeLa FRT cells expressing the indicated BUBR1 mutants. Kinetochore intensities from 45 cells, three experiments. Note that distributions of WT BUBR1 and ΔPP2A(ΔK) conditions are the same as shown in Fig 3I. In (L), the top panel shows the protocol used to visualise chromosome misalignment in fixed samples (see Materials and Methods for details). Graph in bottom panel displays mean frequencies of chromosome misalignments (± SEM) of three experiments, 100 cells quantified per condition per experiment. Note that the mean frequencies of WT BUBR1 condition are the same as shown in Fig 3J. Panel (M) shows the number of kinetochores positive for MAD1 was measured as a readout of unattached kinetochores. The measurement was performed on 30–40 cells from 3 to 4 experiments, before and after a cold-shock treatment to disrupt unstable kinetochore–microtubules attachments. Note that distributions of WT BUBR1 condition are the same as shown in Fig 3K. Treatment with MG132 (10 μM) was included in (L and M) to prevent cells from exiting mitosis.

Data information: Kinetochore/foci intensities in (E–I) are normalised to the WT BUBR1 condition, while in (K) to the ΔPP2A(ΔK) condition. Violin plots show the distributions of kinetochore/foci intensities (E–I and K), the distributions of mitotic duration (C and J) or the distributions of the number of MAD1-positive kinetochores (M) between cells. For each violin plot, each dot represents an individual cell, the horizontal line represents the median and the vertical one the 95% CI of the median, which can be used for statistical comparison of different conditions (see Materials and Methods).

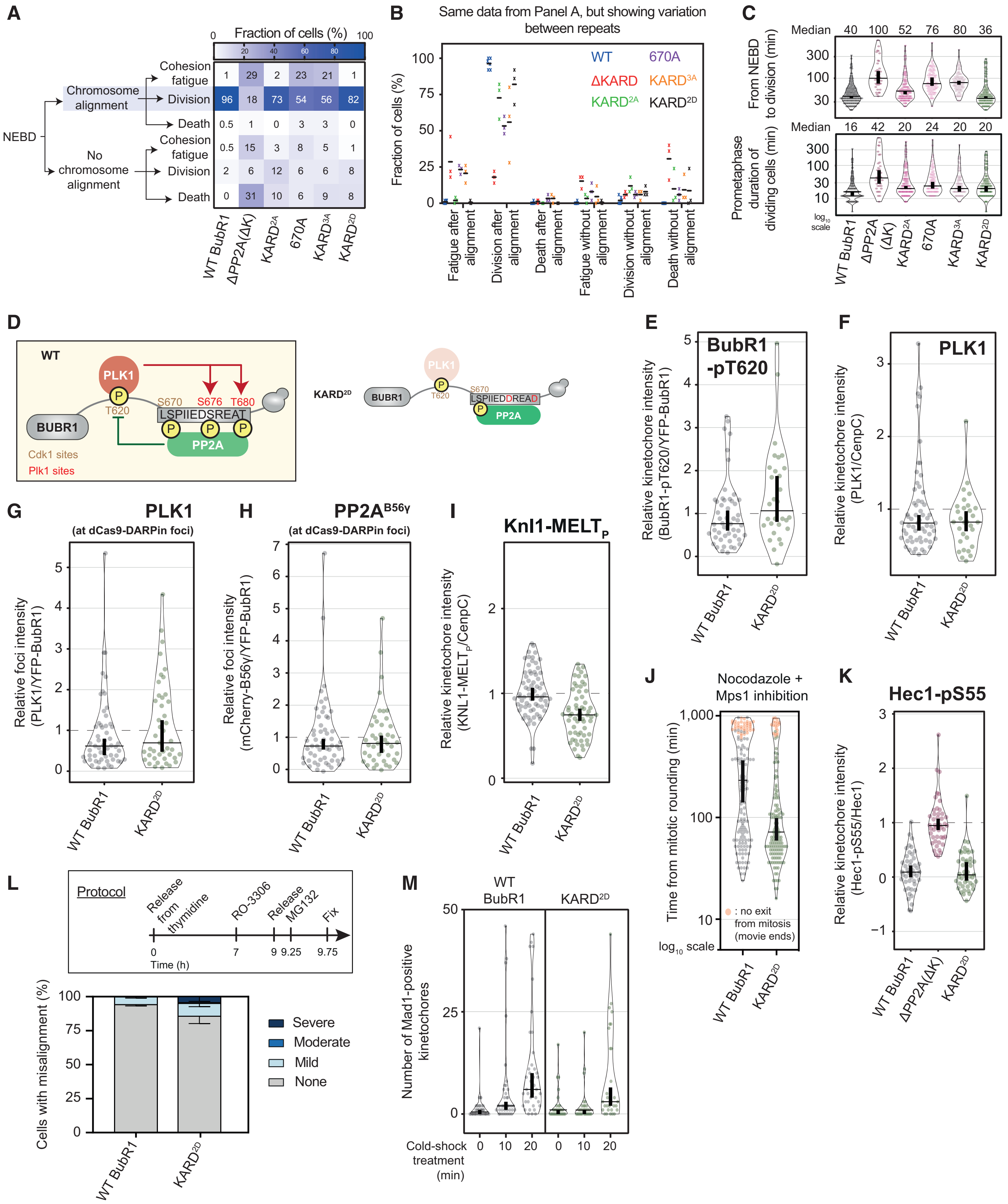
A
Fraction of cells (%)
NEBD
Chromosome alignment
No chromosome alignment
Cohesion fatigue
Division
Death
WT BubR1
ΔPP2A(ΔK)
KARD2A
670A
KARD3A
KARD2D
B
Same data from Panel A, but showing variation between repeats
Fraction of cells (%)
WT
670A
ΔKARD
KARD3A
KARD2A
KARD2D
Fatigue after alignment
Division after alignment
Death after alignment
Fatigue without alignment
Division without alignment
Death without alignment
C
Median 40 100 52 76 80 36
From NEBD to division (min)
Median 16 42 20 24 20 20
Prometaphase duration of dividing cells (min)
log10 scale
D
WT
PLK1
BUBR1
S670 S676 T680
LSPIIEDSREAT
T620
PP2A
Cdk1 sites
Plk1 sites
KARD2D
LSPIIEDDREAD
E
BubR1 -pT620
Relative kinetochore intensity (BubR1-pT620/YFP-BubR1)
F
PLK1
Relative kinetochore intensity (PLK1/CenpC)
G
PLK1 (at dCas9-DARPin foci)
Relative foci intensity (PLK1/YFP-BubR1)
H
PP2AB56γ (at dCas9-DARPin foci)
Relative foci intensity (mCherry-B56γ/YFP-BubR1)
I
Knl1-MELTP
Relative kinetochore intensity (KNL1-MELTP/CenpC)
J
Nocodazole + Mps1 inhibition
Time from mitotic rounding (min)
: no exit from mitosis (movie ends)
log10 scale
K
Hec1-pS55
Relative kinetochore intensity (Hec1-pS55/Hec1)
L
Protocol
Release from thymidine
RO-3306
Release MG132
Fix
Time (h)
Cells with misalignment (%)
Severe
Moderate
Mild
None
M
WT BubR1
KARD2D
Number of Mad1-positive kinetochores
Cold-shock treatment (min)

Figure EV3.

Figure EV4. (Related to Fig 4). Recruitment of KNL1, BUB complex, PLK1 and PP2A to unattached kinetochores in KNL1 MELT mutants.

A–C Levels of YFP-KNL1 (A), BUB1 (B) and BUBR1 (C) at unattached kinetochores relative to CenpC, in nocodazole-arrested HeLa FRT cells expressing the indicated KNL1 MELT mutants. Measurements performed on the same cells are shown in Fig 4C. Kinetochore intensities from 40 to 120 cells, 3–10 experiments.

D, E Fluorescence recovery after photobleaching (FRAP) measurements of BUB1 (D, top graph), BUBR1 (D, bottom graph) and KNL1 (E) at unattached kinetochores, in nocodazole-arrested HeLa FRT cells expressing WT, 6xMELT or 19xMELT KNL1 mutants. For each condition, the mean recovery is represented by a thick line, the 95% CI of the mean with a shaded area and the mobile fraction of the recovery as percentage at the end of each curve (see also (F), Appendix Fig S3 and Materials and Methods for details). Number of cells from three experiments for BUB1 FRAP: 24 for WT Knl1, 25 for 6xMELT and 33 for 19xMELT. Number of cells from three experiments for BUBR1 FRAP: 30 for WT Knl1, 24 for 6xMELT and 24 for 19xMELT. Number of cells from three experiments for KNL1 FRAP: 54 for WT Knl1, 49 for 6xMELT and 57 for 19xMELT.

F Parameters related to the FRAP curves reported in panels (D) and (E) after fitting with a double-exponential law (see Materials and Methods for details).

G, H Estimates of the number of KNL1 molecules and active MELT motifs per kinetochore, in nocodazole-arrested HeLa FRT cells expressing the indicated KNL1 MELT mutants. The table in panel (G) reports the estimates per each KNL1 MELT mutants (see Materials and Methods for details). The graph in panel (H) shows the number of KNL1 molecules (top) or the number of active KNL1-MELT motifs (bottom) plotted against the number of active MELTs per KNL1 molecule, using the data and the assumptions from (G). Data points in panel (H) (bottom) were fitted with an exponential plateau law (see Materials and Methods for details). The fitted curve is reported in the graph, together with the 95% CI of the fit and the estimated plateau. Goodness of fit: 98.69% (based on adjusted R^2).

I, J The levels of YFP-KNL1 (I) and efficiency of BUB1 knockdown (J) at unattached kinetochores in nocodazole-arrested HeLa FRT cells expressing WT or 19xMELT KNL1 mutants and knocked down for GAPDH or BUB1. Kinetochore intensities from 30 to 90 cells, 3–6 experiments.

K, L Levels of PLK1 (K) and mCherry-B56γ (L) at unattached kinetochores relative to CenpC, in nocodazole-arrested HeLa FRT cells expressing the indicated KNL1 MELT mutants. The measurements were performed on the same cells shown in Fig 4D. Kinetochore intensities from 30 to 80 cells, 3–4 experiments.

Data information: Kinetochore intensities are normalised to the WT KNL1 condition (A–C, K and L) or to the WT KNL1 siGAPDH condition (I and J). Violin plots show the distributions of kinetochore intensities. For each violin plot, each dot represents an individual cell, the horizontal line represents the median and the vertical one the 95% CI of the median, which can be used for statistical comparison of different conditions (see Materials and Methods).

© 2023 The Authors

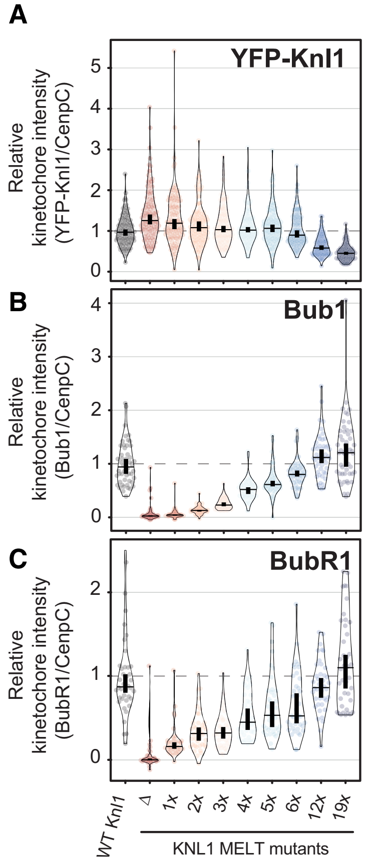

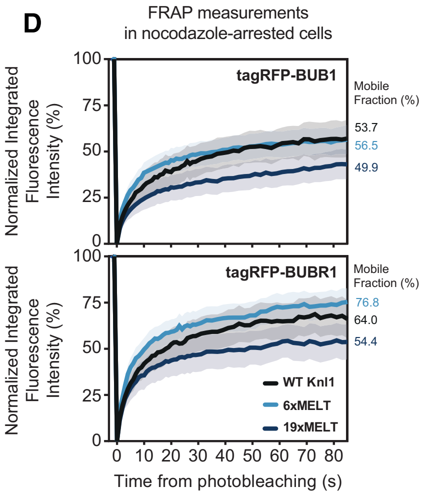

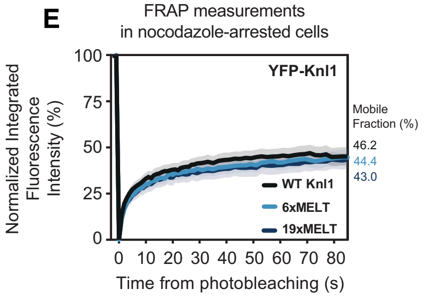

F

	Fitting with double-exponential law	*N* (number of cells)	Mobile Fraction (%)	$t_{1/2}$ fast recovery (s)	$t_{1/2}$ slow recovery (s)	Goodness of fit (adjusted R^2)
tagRFP-BUB1	WT Knl1	24	53.7	8.9	9.0	94.32%
	6xMELT	25	56.5	1.8	16.6	99.74%
	19xMELT	33	49.9	2.5	44.6	99.58%
tagRFP-BUBR1	WT Knl1	30	64.0	7.0	7.0	94.40%
	6xMELT	24	76.8	2.1	22.3	99.61%
	19xMELT	24	54.4	1.8	16.7	99.61%
YFP-Knl1	WT Knl1	54	46.2	1	13.2	99.66%
	6xMELT	49	44.4	1.4	17.2	99.48%
	19xMELT	57	43.0	1.3	17.5	99.24%

G

Assumptions:

- 151 KNL1 molecules per kinetochore (Suzuki et al. 2015)
- ~7 active MELTs in WT Knl1 (Vleugel et al. 2015)

*From Figure EV4 (A,I)

	Active MELTs per KNL1 molecule	Median levels of YFP-KNL1*	KNL1 molecules per kinetochore	Active MELTs per kinetochore
WT	7	0.9692	151	1057
ΔMELT	0	1.254	195	0
1x	1	1.19	185	185
2x	2	1.081	168	336
3x	3	1.031	161	483
4x	4	1.02	159	636
5x	5	1.064	166	830
6x	6	0.8968	140	840
12x	12	0.5803	90	1080
19x	19	0.4502	70	1330
WT siGAPDH	7	0.9673	151	1057
WT siBUB1	7	1.151	179	1253
19x siGAPDH	19	0.3224	50	950
19x siBUB1	19	0.6051	94	1786

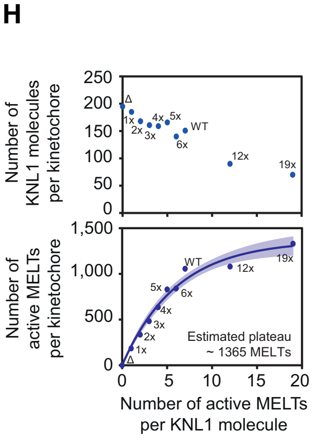

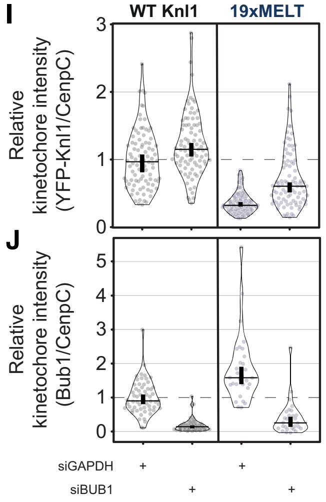

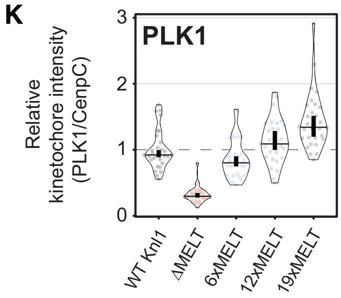

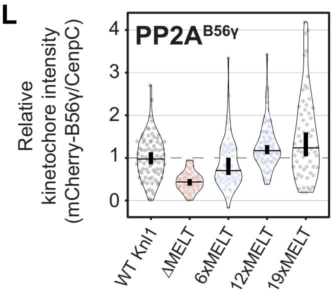

Figure EV4.

© 2023 The Authors

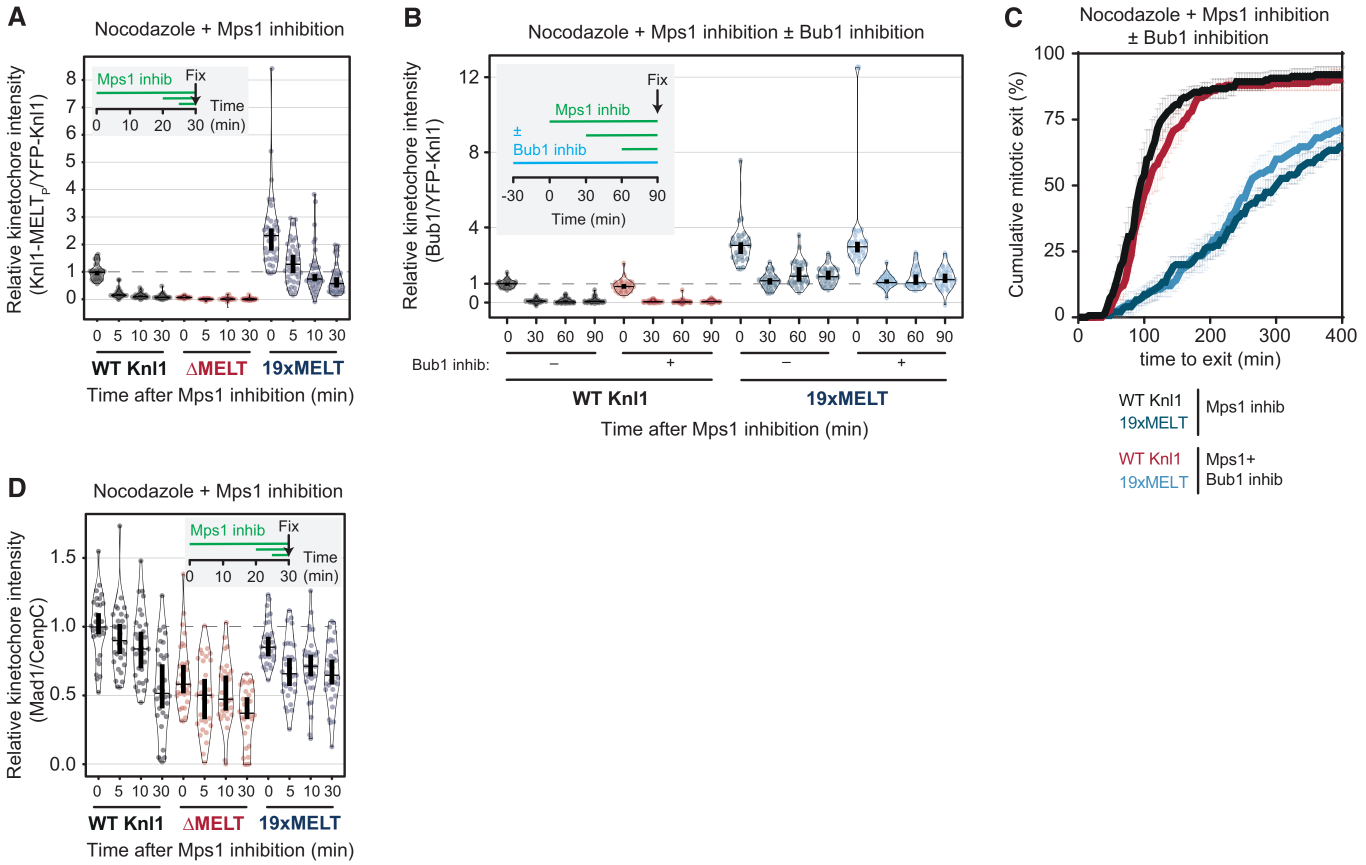

Figure EV5. (Related to Fig 5). Molecular and phenotypic details of SAC signalling after modulation of KNL1 MELT numbers.

A Evaluation of KNL1-pMELT levels at unattached kinetochores, in nocodazole-arrested HeLa FRT cells expressing the indicated KNL1 mutants and treated with the MPS1 inhibitor AZ-3416 (2.5 μM). Kinetochore intensities from 30 cells, three experiments. Treatment with MG132 (10 μM) was included to prevent mitotic exit after the addition of the MPS1 inhibitor.

B, C Evaluation of the role of BUB1 kinase activity in sustaining the SAC signalling, in terms of BUB1 levels at unattached kinetochores (B) and the duration of the mitotic arrest (C), in nocodazole-arrested HeLa FRT cells expressing the indicated KNL1 mutants and treated with the MPS1 inhibitor AZ-3146 (2.5 μM), with or without the BUB1 inhibitor BAY-1816032 (5 μM). Panel (B) shows kinetochore intensities from 30 cells, in three experiments. Note that distributions of WT KNL1 and 19xMELT without BUB1 inhibition are the same as shown in Fig 5C. The graph in panel (C) shows mean (± SEM) of three experiments, 50 cells per condition per experiment. Treatment with MG132 (10 μM) was included in (B) to prevent mitotic exit after the addition of the MPS1 inhibitor.

D Levels of MAD1 at unattached kinetochores, in nocodazole-arrested HeLa FRT cells expressing the indicated KNL1 mutants and treated with the MPS1 inhibitor AZ-3416 (2.5 μM). Kinetochore intensities from 30 cells, three experiments. Treatment with MG132 (10 μM) was included to prevent mitotic exit after the addition of the MPS1 inhibitor.

Data information: Kinetochore intensities are normalised to the WT KNL1 condition at time 0. Violin plots show the distributions of kinetochore intensities. For each violin plot, each dot represents an individual cell, the horizontal line represents the median and the vertical one the 95% CI of the median, which can be used for statistical comparison of different conditions (see Materials and Methods).

© 2023 The Authors
