# Supplementary material for: A bifunctional kinase–phosphatase module balances mitotic checkpoint strength and kinetochore–microtubule attachment stability
Source: EMBO J. 2023 Sep 15;42(20):e112630. doi: 10.15252/embj.2022112630 (PMC10577578; doi:10.15252/embj.2022112630)
Supplement: Supplementary file 7 [file EMBJ-42-e112630-s001.pdf]

A bifunctional kinase-phosphatase module balances mitotic checkpoint strength and kinetochore-microtubule attachment stability

Andrea Corno, Marilia Cordeiro, Lindsey Allan, Qian-Wei Lim, Elena Harrington, Richard Smith, and Adrian Saurin
DOI: 10.15252/embj.2022112630

Corresponding author(s): Adrian Saurin (a.saurin@dundee.ac.uk)

Review Timeline:

Submission Date:	16th Sep 22
Editorial Decision:	4th Oct 22
Revision Received:	2nd Jun 23
Editorial Decision:	7th Jul 23
Revision Received:	23rd Aug 23
Accepted:	28th Aug 23

Editor: Hartmut Vodermaier

Transaction Report: This manuscript was transferred to The EMBO JOURNAL following peer review at Review Commons.

Review #1

1. Evidence, reproducibility and clarity:

Evidence, reproducibility and clarity (Required)

The proper segregation of chromosomes during mitosis depends on correct kinetochore-microtubule (KT-MT) attachments which is monitored by the spindle assembly checkpoint (SAC). The Bub complex composed of the Bub1-Bub3 and BubR1-Bub3 complexes plays an important role in regulating KT-MT by recruiting Plk1 and PP2A-B56 as well as the SAC components Mad1/Mad2 and Cdc20. The Bub complex is recruited to kinetochores by Bub3 binding directly to phosphorylated MELT repeats on KNL1. The MELT repeats are predominantly phosphorylated by Mps1 but can also be phosphorylated by Plk1.

Here the authors investigate the Plk1 and PP2A-B56 module on BubR1 further. It is already known that Plk1 is recruited to BubR1 T620 (a Cdk1 site) and that this is important for proper chromosome segregation. Furthermore, BubR1 binds to PP2A-B56 through and LxxIxE motif that is phosphorylated by Plk1 to stimulate binding. The main message of the paper is that the Plk1-PP2A-B56 module on BubR1 is crucial for integrating SAC and KT-MT attachments and that a homeostatic negative feedback loop between Plk1 and PP2A-B56 exists to limit the levels of these enzymes. As outlined below I do not think that their experimental evidence/setup can be used to draw these conclusions. Overall, I have limited comments on the technical execution of experiments as this is overall done well but more on interpretations of results and what conclusions can be drawn. Also, most of the phenotypes/observations are consistent with the literature and not suprising.

Major comments:

The authors generate a scenario where high levels of Plk1 are recruited to BubR1 by removing the entire C-terminus to remove the PP2A-B56 binding site and a situation of high PP2A-B56 by replacing the BubR1 C-term with B56gamma. Firstly, these are crude mutants generating extreme situations - specifically the fusion of B56gamma which likely creates artificially high levels of BubR1-B56gamma making it difficult to make conclusions on the physiological levels. These mutants are then analysed in a number of assays and phenotypic consequences analysed in the presence of nocodazole + Mps1 to evaluate SAC strength. It would be interesting to see what the phenotypes are without Mps1 inhibition. I cannot see in any way how the authors from two end points (high kinase or high phosphatase) can reach a conclusion that a

homeostatic negative feedback loop exists between these enzymes that is critical for integrating SAC and KT-MT interactions. They can only conclude on what happens if you have too much kinase or phosphatase but no data are addressing what happens if you manipulate the cross-talk on BubR1. The experiments to do must be to carefully tune the proposed cross talk and then monitor what happens. This can be done by bypassing Plk1 regulation of the PP2A-B56 binding site or increasing the strength of the Plk1 binding site. Furthermore, there is no data to show that cross-talk is changing in response to changes in KT-MT attachment status - is the levels and activities of Plk1/PP2A-B56 on BubR1 changing. Yes Plk1 and PP2A-B56 can regulate each other on BubR1 but is this regulated as proposed.

My second comment relates to the fact that the two parts of the paper are not directly linked although the authors try to do this. They nicely manipulate the MELT repeats on KNL1 to change the number of Bub complexes. However, they cannot directly link the data to changes in Plk1 and PP2A-B56 levels only as many other things are changing. By increasing MELT numbers Bub complex and Mad1/Mad2 levels increase as well as an example and this makes interpretations complicated. To me these experiments are not addressing the main conclusions of the paper.

Specific comments:

1. I would caution the interpretation of phenotypes being suppressed by Plk1 inhibitors. This does not address whether it is BubR1 bound Plk1 that is specifically affected - several Plk1 substrates could be contributing. Similar for Mad1 phosphorylation they cannot conclude that it is Plk1 bound to BubR1 phosphorylating Mad1.
2. On page 4 the authors write: "We sought to modulate MELT numbers in a way that would allow BUB complex levels to be increased or decreased in a graded manner, thereby causing reciprocal changes to PLK1 and PP2A levels." I do not see how this will result in reciprocal levels as total Bub complex levels are increased.
3. Page 7 first paragraph authors write: "remained high despite inhibition of Mps1". This is not completely correct as levels are dropping dramatically after 5/10 min of Mps1 inhibition. Total drop seems more than WT situation.
4. Page 7 second paragraph authors write: "implying the elevated PLK1 in this situation (Figure 3E) is also able to better amplify MELT signalling..." This cannot be concluded as the starting levels are so much higher in 19XMELT than WT and there is no data to show this is due to more Plk1 bound to BuBR1.
5. Page 7 second section authors write: "Figure 5B shows that KNL1deltaMELT causes severe chromosome misalignments, as expected, given the PP2A-B56 binding to KNL1 is inhibited in this situation". Multiple things are changing so this cannot be interpreted only as a readout of PP2A-B56. With no MELTs there is no recruitment of

SAC proteins.
6. Page 7 second section authors write: "Therefore, the number of MELT motifs is crucial for determining the stability of KT-MT attachments, most probably by setting the levels of PP2A-B56..." Again many things are changing so impossible to interpret data in light of PP2A-B56.
7. One possibility not mentioned by authors is that PP1 bound to KNL1 cannot act as efficiently on some MELT repeats and that the dephosphorylation by PP1 of the 19xMELT is different from KNL1 WT which would impact on their results.

2. Significance:

Significance (Required)

See above.

3. How much time do you estimate the authors will need to complete the suggested revisions:

Estimated time to Complete Revisions (Required)

(Decision Recommendation)

Cannot tell / Not applicable

4. Review Commons values the work of reviewers and encourages them to get credit for their work. Select 'Yes' below to register your reviewing activity at Publons; note that the content of your review will not be visible on Publons.

Reviewer Publons

Yes

Review #2

1. Evidence, reproducibility and clarity:

Evidence, reproducibility and clarity (Required)

In this manuscript by Corno et al the authors investigate how the kinase/phosphatase balance is regulated at kinetochores during mitosis. When kinetochores are unattached, mitotic kinases signal through the spindle assembly checkpoint (SAC) to prevent progression through mitosis. Once attachments have occurred, the activities of kinetochore phosphatases silence the SAC and stabilize kinetochore-microtubule attachments to promote exit from mitosis. Key regulators of this signaling are the BUB proteins Bub1 and BubR1, which recruit the kinase Plk1. BubR1 also recruits the phosphatase PP2A-B56 in a Plk1-dependent manner. By analyzing the recruitment of Plk1 and PP2A-B56 at unattached kinetochores, the authors uncovered a mechanism by which Plk1 and PP2A-B56 negatively regulate each other's recruitment, thereby maintaining an optimal kinase/phosphatase balance. Disrupting this balance can lead to either a hyperactive SAC or premature stabilization of erroneous attachments.

Overall, this is a very careful study and the data is clear and consistent. My only comments are in regards to the interpretation of the Knl1 data on the manuscript.

Comments

- In figures 3 and 4, the authors engineer a system to manipulate the levels of BUBs at the kinetochore by modulating the number of MELT repeats on Knl1. For this, they mutate all 19 MELT repeats on Knl1 and then add back discreet number of MELT motifs that follow a consensus sequence. They find that a 6x mutant, which contains 6 repeats of this consensus MELT motif, is sufficient to rescue the functions of Knl1 on the SAC and on chromosome alignment. However, 12x and 19x MELT repeat versions show an increased SAC response and increased stability of kinetochore-microtubule attachments. The authors interpret this as a result of increased kinetochore levels of BUBs, and therefore, increased levels of both Plk1 and PP2A-B56 (Fig. 3). However, from their representative images in Fig. S3A, it does not appear as if BUB levels are significantly increased in cells expressing the 12x and 19x Knl1 mutants, compared to wild-type controls. Considering that in the 12x and 19x mutants Knl1 recruitment is reduced by more than half of controls (Fig. S3B) and because the authors normalized their BUB kinetochore intensity levels by the Knl1 values, it makes it seem as if BUB kinetochore levels are increased in the cell under these conditions. While in the 12x and 19x mutant conditions there are more molecules of BUBs per Knl1, the overall BUB levels are the same as in wild-type controls. Since the MELT repeat used throughout the paper is a consensus sequence

that is likely optimal for BUB binding, it is possible that the phenotypes of the 12x and 19x mutants are explained because of an increase in the affinity of BUBs for Knl1 rather than overall levels. This would also help explain why Knl1 and BUBs are observed at the spindle midzone in the 19x mutant (Fig. S4). To distinguish between these possibilities, the authors might consider doing FRAP experiments using fluorescently labelled Bub1 or BubR1 and measure BUB protein dynamics at kinetochores.
- Also in regards to the 12x and 19x mutants, because these reduce Knl1 kinetochore levels, this fact alone might explain some of the observed phenotypes, such as the mild defects in chromosome alignment (Fig. S5).

2. Significance:

Significance (Required)

This is a significant advancement in our understanding of how the spindle assembly checkpoint is regulated by kinases and phosphatases at the kinetochore. The authors used very precise manipulations to dissect how these components are balanced and they uncovered a very interesting negative feedback mechanism. They also provided significant evidence for the importance of this balance in normal mitotic progression. This work will be of broad interest to cell biologists, as well as cancer biologists.

My expertise is on the fields of cell biology, mitosis and cell division mechanisms.

3. How much time do you estimate the authors will need to complete the suggested revisions:

Estimated time to Complete Revisions (Required)

(Decision Recommendation)

Between 1 and 3 months

4. Review Commons values the work of reviewers and encourages them to get credit for their work. Select 'Yes' below to register your reviewing activity at Publons; note that the content of your review will not be visible on Publons.

Reviewer Publons

Yes

Review #3

1. Evidence, reproducibility and clarity:

Evidence, reproducibility and clarity (Required)

A dividing cell relies on both error correction and spindle assembly checkpoints (SACs) to ensure accurate chromosome segregation. The PLK1-PP2A pair resides at the interface of the two pathways and makes these pathways robust and responsive. By generating fusion proteins and mutagenesis to perturb PLK1 and PP2A activities in HeLa cells, the authors found that both enzymes constitute a negative feedback loop on the scaffold BubR1. The docked PLK1 phosphorylates the PP2A binding site to recruit PP2A, while the PLK1 docking site is dephosphorylated after PP2A recruitment. They also manipulate kinetochore recruitment of PP2A to show effects on the SAC and on kinetochore-microtubule attachments. Independently, by varying active MELT repeats on KNL1, the authors found that decreasing MELT repeats weakens the SAC with unstable attachments, while increasing MELT repeats strengthens the SACs with hyperstable attachments. Thus, the recruited level of BubR1 determines SAC strength and attachment stability. The data included in this paper are solid, and the concepts are interesting, but these potential strengths of the manuscript are obscured because the logical progression and presentation are difficult to follow.

Major comments:

1. The first three paragraphs of the introduction lead up to the question: "Why the same phosphatase complex is used for both process is still not clearly understood". One conceptually simple answer is that the SAC should be silenced as attachments are stabilized, so it makes sense to use the same enzyme to accomplish both tasks. If the authors have something else in mind, they should clarify.
2. The argument linking the negative feedback loop to biological functions is not straightforward. The authors provide evidence in Figure 1 for regulatory pathways between docked PLK1 and bound PP2A. However, their assays in Figure 2 bypass the feedback loop by directly modulating PP2A activity. These experiment supports an

argument that the kinase/phosphatase activity balance is important, but do not address the feedback loop specifically (which could potentially be done using mutations that disrupt the feedback regulation). The claim that "a homeostatic feedback loop maintains an optimal balance of PLK1 and PP2A on the BUB complex" is too strong because there is no direct evidence connecting the feedback loop to optimal function.
3. The paragraph starting with "Given the roles of PLK1 and PP2A ...": How does the kinase-dominant situation destabilize KT-MT attachments? Is it by inhibiting PP2A or by phosphorylating some kinetochore components? The former pathway is unclear because the authors show that PLK1 promotes PP2A recruitment in Figure 1.
4. The paragraph starting with "In summary, a balanced recruitment of PLK1 and PP2A ...": What is meant by "phosphorylation sites that block KT-MT attachment" (used twice in the paragraph)? Block means prevent binding, as opposed to activities that destabilize attachment by promoting unbinding. Which do the authors mean? The following is also unclear: "kinetochores are no longer responsive to MT attachment". If attachment is blocked, as stated in the previous sentence, then what does it mean to say that kinetochores are not responsive to attachment (which never occurred if it was blocked)?
5. Figures 1-2 and Figures 3-5 are separate concepts, but this is never explained clearly in the manuscript. Specifically, Figures 1-2 focus on the antagonism between PLK1 and PP2A activities, whereas Figures 3-5 focus on changing both activities in the same direction (either increase or decrease). There is no transition from one part to the other. Both concepts should be explained and the hypotheses stated clearly.
6. Several words are used ambiguously. "Homeostasis" is vague, and it is unclear what exactly the authors mean. As discussed above, the meaning seems different for figures 1-2 vs figures 3-5. "Reciprocal changes" is also unclear (and seems misleading) because the perturbations of PLK1 and PP2A levels are in the same direction (more MELT motifs means more binding sites for both). For "preserved" (description of figure 4F), it's unclear if the authors refer to the Bub1 and BubR1 levels at metaphase or the change between prometaphase and metaphase. The authors should clarify what they mean and how it is measured.
7. "too much kinase-phosphatase module would cause a strong SAC and hyperstable KT-MT attachment, and too little would cause a weak SAC and hypostable KT-MT attachments": The reasoning behind these predictions is not clear. For example, why does high phosphatase not silence the SAC as explained earlier in the manuscript?
8. The mitotic exit assay in Figure 4G is hard to interpret. Mitotic duration depends on establishing correct attachments and then silencing the SAC. 19xMELT could affect both. A better measurement of SAC silencing would be time from metaphase alignment to anaphase.
9. The paragraph starting "In summary, PLK1 is able to phosphorylate MELTs to recruit Bub1": The authors should clarify what was already known and what advance they are making. Similarly, the sentence starting with "Therefore, the number of

MELT motifs ..." should also clarify the advance relative to previous findings.

Minor comments:

1. Please include pages numbers (and line numbers are also helpful).
2. Previous literature (PMID 17785528) suggests that phosphorylation of pT620 is important for KT-MT regulation but not SAC signaling. Can the authors comment on this?
3. SAC silencing seems more appropriate than "mitotic exit" in the last sentence of the second paragraph.
4. Figure 1 would be clearer with images and the relevant quantifications together in the same panel.
5. In the first paragraph of Results, the authors primarily explain the impacts of PP2A on SAC silencing but not on KT-MT attachment, even though the topic sentence seems equally weighted to both.
6. Some terms are not defined when first introduced, such as the KARD domain and B56gamma.
7. Figure 1JK: how were mitotic cells enriched and harvested?
8. Figure 2C: a log scale may help show changes in both directions.
9. Chromosome alignment assays in Figure 2 are not so informative because perturbation either way can generate misaligned chromosomes. The primary figures are dense with data, so these results can be made supplemental.
10. Figure 3F: can the authors comment on the B56gamma decrease between nocodazole and MG132 conditions?
11. Figure 4A: the data are difficult to interpret as presented. It is not clear whether SAC signaling changes monotonically with number of MELT repeats. It would be better to plot MELT number vs a summary statistic (such as time to 50% mitotic exit or something else that the authors find informative).
12. Figure 6. "uncouplr" should be "uncouple".
13. Missing references in bibliography: Ghongane et al 2014, Roy et al. 2020.

2. Significance:

Significance (Required)

The proposed model is conceptually significant, and this mechanistic work bridges several previous findings. Biochemical studies suggest that KNL1 harbors PLK1-PP2A and Bub complex, and functional studies suggest that truncation on MELT motifs generates mitotic errors (PMID 24363448, 24344183). Furthermore, biochemical and functional assays suggest that PLK1 is a key regulator of the SAC (PMID 33125045). In this work, the authors link functional consequences to

biochemical interactions among KNL1, BubR1, PLK1, and PP2A, which is an advance.

This work should appeal to the kinetochore and cell cycle communities, but the logical flow needs to be improved.

My most relevant expertise is in mitotic kinases and regulation of kinetochore microtubules.

3. How much time do you estimate the authors will need to complete the suggested revisions:

Estimated time to Complete Revisions (Required)

(Decision Recommendation)

Between 1 and 3 months

4. Review Commons values the work of reviewers and encourages them to get credit for their work. Select 'Yes' below to register your reviewing activity at Publons; note that the content of your review will not be visible on Publons.

Reviewer Publons

Yes

Revision Plan

Manuscript number: RC-2022-01537
Corresponding author(s): Adrian T, Saurin

1. **General Statements** [optional]

We thank all reviewers for providing very detailed, knowledgeable, and informative reviews.

All reviewers were complementary about the data and the rigor of the study. Reviewers 2 and 3 commented on the significance of the work, and their assessments were complementary, specifically about the fact that it bridges several previous studies and links these to kinase-phosphatase regulation on the BUB complex. We agree that this a major strength of the work. That is why we also believe the comment by reviewer 1 that "most of the phenotypes/observations are consistent with the literature and not surprising" is actually a strength and not a weakness. Sometimes manuscripts that bring together various different findings into one conceptual model can be very powerful, even if each finding in isolation is not so surprising. In this case, the concept that a dual kinase-phosphatase module integrates two major mitotic processes will, we believe, prove to be a significant breakthrough that helps to explain how these processes are properly integrated at kinetochores.

The main criticism of all reviewers related to the interpretations and writing style, which in general, we felt were valid. We will take on board all these comments, reword the manuscript during revision, and provide a detailed response to each of these points at resubmission.

In terms of points requiring new experiments, there were 3 in total:

1) Reviewers 1 and 3 raised an important issue about the feedback loop which will be addressed with new experiments to uncouple the feedback.

2) Reviewer 2 made an important point about KNL1 levels, including a good suggestion to perform FRAP analysis to examine BUB complex dynamics when MELT numbers are increased. We will carry out this experiment prior to revision.

3) Reviewer 1 had a second major comment regarding the modulation of MELT number and how this cannot be directly linked to PLK1/PP2A levels. We have 3 new experiments to add regarding this, performed already, which are discussed in the section below.

All other comments were textual points that in most case we felt were valid. They showed that all reviewers had a very good grasp of the paper, the concepts, and the field in general. So, we finish by thanking all reviewers again for their thorough and detailed assessments of

our manuscript. The comments they raised will help us to improve the manuscript after revision.

2. Description of the planned revisions

Three main points:

1) The role of the feedback loop [reviewers 1 and 3]:
The general issue is explained succinctly by reviewer 3's comment: *"The argument linking the negative feedback loop to biological functions is not straightforward. The authors provide evidence in Figure 1 for regulatory pathways between docked PLK1 and bound PP2A. However, their assays in Figure 2 bypass the feedback loop by directly modulating PP2A activity. These experiment supports an argument that the kinase/phosphatase activity balance is important, but do not address the feedback loop specifically (which could potentially be done using mutations that disrupt the feedback regulation). The claim that "a homeostatic feedback loop maintains an optimal balance of PLK1 and PP2A on the BUB complex" is too strong because there is no direct evidence connecting the feedback loop to optimal function."*

This is a good point that we will address at revision. We demonstrate that the enzymes regulate each other on the BUB complex in figure 1 (PLK1 recruits PP2A, and PP2A removes PLK1), which balances their levels on the BUB complex. To determine consequences of upsetting this balance we locked either the kinase-bound or phosphatase-bound states (Figure 2). Importantly, this is required to assess direct phenotypes associated with each, but it does not directly demonstrate the role of the feedback loop. To do this we will generate mutants, as suggested, and analyse their phenotypes.

We will mutate the PLK1 binding site (T620A) and recover the PLK1-regulated sites in the KARD motif to phospho-mimicking aspartates (S676D/T680D), analyzing effects on PLK1/PP2A recruitment, chromosome alignment and SAC strength. We predict that this will remove PLK1 and recover some PP2A, but to lower levels overall than the BUBR1-B56 fusion. In that case the phenotypes will probably be milder, but that would not change the overall conclusions.

We maintain that locking PLK1 on its phospho-binding site (in BUBR1-ΔPP2A) is the ideal scenario to test direct PLK1 roles, but we will also now create alanine mutants of the PLK1 site (S676A/T680A) and the CDK1 site (S670A) to address the feedback loops controlled by CDK1 and PLK1. Our prediction is that these will skew the balance towards PLK1, without fully removing PP2A, again likely to produce milder intermediate phenotypes.

It is definitely worth testing these predictions, because it would directly address the role of the feedback loop and it would avoid relying solely on “artificially high levels” as mentioned by reviewer 1. One final point on this however, the PLK1 recruitment in ∆PP2A cells is not artificial – it is PLK1 bound to its native phospho-motif when PP2A is unbound (without any feedback from PP2A this phospho-site and PLK1 binding increase to the observed maximal levels). The fusion of B56 is admittedly less optimal, but this does still lock the phosphatase-bound state in a set stoichiometry, crucially in the absence of kinase. This is required to assess direct phosphatase effects. These PLK1/PP2A levels may well be higher than observed physiologically on the BUB complex when considering the behavior of all BUBR1 molecules, since we doubt they ever reach 1:1 stoichiometry with either PLK1 or PP2A. However, the feedback loop is operating within individual molecules (figure 1), which may well individually flip between PLK1 or PP2A bound states. This may occur on certain molecules at specific times. Therefore, locking the PLK1.PP2A-bound state on all molecules is, in our opinion, still a valid and useful perturbation to assess function of these two states.

2) The increase recruitment of BUB1-PLK1/PP2A when MELT numbers are increased [reviewer 2]

“While in the 12x and 19x mutant conditions there are more molecules of BUBs per Knl1, the overall BUB levels are the same as in wild-type controls. Since the MELT repeat used throughout the paper is a consensus sequence that is likely optimal for BUB binding, it is possible that the phenotypes of the 12x and 19x mutants are explained because of an increase in the affinity of BUBs for Knl1 rather than overall levels. This would also help explain why Knl1 and BUBs are observed at the spindle midzone in the 19x mutant (Fig. S4).”

The reviewer raises an important issue here, when stating that increasing MELT numbers decreases KNL1 kinetochore recruitment. This has the net effect of normalizing overall BUB1-PLK1/PP2A kinetochore levels, even though BUB1-PLK1/PP2A recruitment per KNL1 molecule is increased. That is why we were careful to state BUB1-PLK1/PP2A were increased “on each KNL1 molecule” and not “on kinetochores” when referring to the effect in the 12x/19x MELT mutant. However, this could easily be misinterpreted so this point will be clarified at revision.

The issue of why the phenotype is so dependent on kinase/phosphatase level per KNL1 molecules is an important one however, which has puzzled us until now. We think the suggestion to look at turnover by FRAP is a good one, because enhanced binding strength could underlie the phenotype here, and potentially explain the lack of disassembly at anaphase. We will perform these experiments at revision to see if they can clarify the issue.

3) The link between MELT number and PLK1/PP2A levels [Reviewer 1]

“My second comment relates to the fact that the two parts of the paper are not directly linked although the authors try to do this. They nicely manipulate the MELT repeats on KNL1 to change the number of Bub complexes. However, they cannot directly link the data to

changes in Plk1 and PP2A-B56 levels only as many other things are changing. By increasing MELT numbers Bub complex and Mad1/Mad2 levels increase as well as an example and this makes interpretations complicated. To me these experiments are not addressing the main conclusions of the paper."

We disagree with this overall assessment, but there are two elements to this comment: the effect of modulating MELT number on SAC strength (and PLK1) or on KT-MT stability (and PP2A). We will therefore discuss each separately:

For SAC regulation, we feel that the data is clear and the interpretations are justified, although we will add new data to support this point after revision. Increasing MELT number causes defects in MELT-BUB dissociation and SAC silencing (4a-c). Importantly, these phenotypes can be completely rescued by inhibiting PLK1 (4d-e). So, we do link the effects of high MELT number to PLK1 activity. Our interpretation is that when MELT numbers are increased the ability of PLK1 to phosphorylate these motifs and maintain the SAC platform is enhanced (when MPS1 is inhibited pharmacologically or upon KT-MT attachment). So, whilst it is true that many factors, such as the kinetochore levels of BUB/MAD1/MAD2, are crucial for the SAC, the ability of PLK1 to maintain these levels (via pMELT-BUB1) is crucial and that changes as MELT number increases. This contributes directly to the observe SAC silencing phenotype, as confirmed by the complete rescue of this phenotype after PLK1 inhibition.

We did also explore the possibility that increased BUB1 activity could also contribute to SAC strengthening, for example, by enhancing Aurora B recruitment to centromeres. However, BUB1 inhibition did not alter SAC strength or MELT dephosphorylation kinetics (see Figure R1). We will add this data after revision.

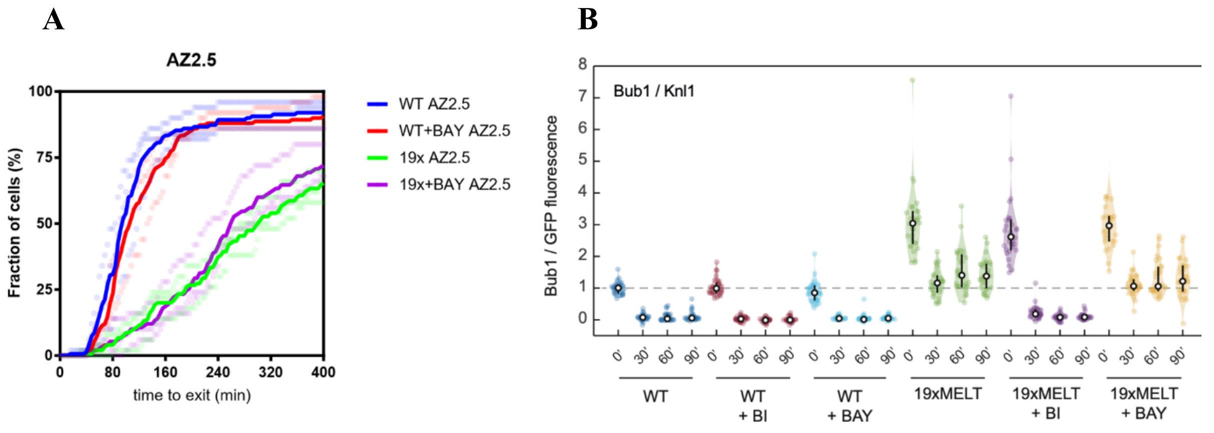

Figure R1) Inhibition of BUB1 kinase activity does not alter SAC strength (A) or BUB1 removal (B) in KNL1-19xMELT mutants after MPS1 inhibition with AZ-3146 (2.5µM). Nocodazole-treated cells, treated with the PLK1 inhibition BI-2536 (100nM) or the BUB1 inhibitor BAY1816032 (5µM). (A) displays the mean (thick line) of 3 experiments (individual dots), 50 cells per condition per experiment. (B) displays kinetochore intensities of 30 cells per condition, 3 experiments. The timepoints on the bottom part of the graph reports the incubation time with the MPS1 inhibitor. Treatment with MG132 was included to prevent mitotic exit after the addition of the MPS1 inhibitor. Abbreviations: AZ2.5 = AZ-3146; BI = BI-2536; BAY = BAY1816032.

We also evaluated the levels of phosphorylated MAD1-pT716, which is important for MCC assembly (Ji et al. 2017, Ji et al. 2018, Faesen, 2017). Our data show that WT and 19xMELT exhibit similar MAD1-pT716 levels during a nocodazole arrest and following MPS1 inhibition (Figure R2). In summary, the main changes we observe are elevated BUB1 levels due to MELT phosphorylation, and increased BUB1 phosphorylation on pT461 (as shown in Figure 4h). All this points towards a localized effect of PLK1 on/around the BUB complex. We will add this data and make this point clearly at revision.

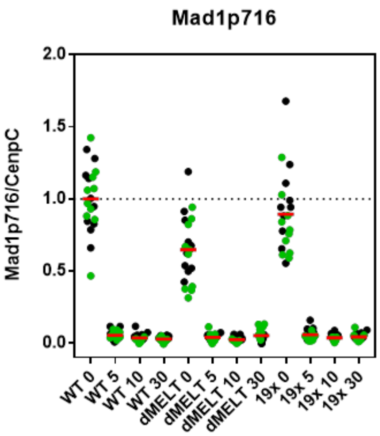

Figure R2) WT KNL1 and 19xMELT cells exhibit similar levels of MAD1-pT716 at kinetochores before/after MPS1 inhibition. MPS1 inhibitor AZ-3146 (2.5 µM). Kinetochore intensities from 20 cells, 2 experiments. The timepoints on the bottom part of the graph reports the incubation time (in minutes) with the MPS1 inhibitor. Treatment with MG132 was included to prevent mitotic exit after the addition of the MPS1 inhibitor. For each distribution, the mean value is represented with a red line.

For KT-MT attachment regulation, we agree that we do not have a similar way to inhibit PP2A-B56 activity to rescue hyperstable microtubule attachment when MELT numbers are high. For this, we require a way to rapidly inhibit PP2A-B56 activity after attachments have formed, something that is not technical feasible at the present. We can also not say for certain that reduced MELT numbers destabilize microtubule due to lack of PP2A, however we feel this is the most like interpretation for the following reasons. The phenotype of removing PP2A from BUBR1 or removing the MELT from KNL1 (along with all associated factors), is identical: mutant cells have comparable chromosome misalignment due to unattached kinetochores (compare 2F-I with 5A-D). Therefore, the additional factors lost by removing the MELTs cannot be having such a strong impact in KT-MT attachment. The obvious factor that could affect attachment strength is again BUB1, via Aurora B recruitment to centromeres. However, loss of BUB1 (after MELT removal) is predicted to enhance attachment stability (reduced Aurora B) and not decrease it, as we observe. So, whilst we cannot definitely conclude that modulating MELT number affect attachment stability via

PP2A, we feel that this is certainly the most likely explanation. We will state this clearly in the revised text.

3. Description of the revisions that have already been incorporated in the transferred manuscript

n/a

4. Description of analyses that authors prefer not to carry out

1 Minor point:

"SAC strength of BubR1 WT, ΔC and B56γ was analysed in the presence of nocodazole + MPS1i. It would be interesting to see what the phenotypes are without MPS1i [Reviewer 1]"

In the absence of MPS1i basal MELT phosphorylation increases (ΔC) or decreases (B56γ) as predicted (Figure 2d; compare timepoint 0 all conditions). This does not cause any change to SAC strength when all kinetochores are unattached in nocodazole (not shown). The sensitize SAC assay (nocodazole + MPSi) has been used by many groups (originally Santaguida et al, 2011; Saurin et al, 2011), because it reduces SAC signals from all unattached kinetochores which would otherwise produce a saturated response. In this case, we specifically chose a dose of MPS1 inhibitor that gave a partial SAC response from which we could observe either strengthening or weakening – a key point of the assay. Indeed, this showed that the SAC was strengthened (ΔC) or weakened (B56γ), as predicted (Figure 2E). The only other way to do this, which has been used by some in the literature, is to use a low dose of nocodazole which prevents all kinetochore from signaling to the SAC. We specifically wanted to avoid this situation because then you cannot untangle the effects on SAC and KT-MT attachment stability – this was crucial in our case.

Dear Adrian,

Thank you for submitting your manuscript on a bifunctional kinase-phoshpatase module at kinetochores, together with reports from Review Commons, for our editorial consideration. I apologize for the delay in getting back to you with an intial response - after having read and discussed the submission with my colleagues, I wanted to additionally run your responses and revision plan by some of the original referees before taking an initial decision. The reason was that although I appreciated your thorough and well-thought-out approach to the question of why/how the same kinase/phosphatase module can be employed in two distinct functions, I noticed that especially referees 1 and 3 appeared not fully convinced that the results provided a sufficiently clear and discrete advance of our current understanding; and I was unsure whether the proposed revisions would suffice to alleviate these concerns.

Original referee 1 has now gotten back to me with the comment below, in light of which I shall be happy to invite you to prepare a formal revision for The EMBO Journal, along the lines outlined in your revision plan. As usual, the study will be "scooping-protected" while under our consideration, and we would also be open to offer an extended revision deadline if needed to decisively address the reviewers' criticisms. Please note that the final decision on publication in our journal will obviously depend to some extent on the outcome of the key revision experiments in this case.

At the stage of resubmission, please also take care to adhere to the editorial points listed below, and to follow our Guide to Authors regarding formatting and contents of a revision for our journal - happy to answer any questions you may have in this regard!

With best regards,

Hartmut

Hartmut Vodermaier, PhD
Senior Editor, The EMBO Journal
h.vodermaier@embojournal.org

REFEREE 1

I went through the comments and revision plan. I think they have outlined some clear plans for addressing the concerns of all reviewers and responded constructively to my concerns. Depending on the outcome of these new experiments it could substantially strengthen the paper and make it of interest to the broader audience of EMBO J.

*** PLEASE NOTE: All revised manuscript are subject to initial checks for completeness and adherence to our formatting guidelines. Revisions may be returned to the authors and delayed in their editorial re-evaluation if they fail to comply to the following requirements (see also our Guide to Authors for further information):

1) Every manuscript requires a Data Availability section (even if only stating that no deposited datasets are included). Primary datasets or computer code produced in the current study have to be deposited in appropriate public repositories prior to resubmission, and reviewer access details provided in case that public access is not yet allowed. Further information: embopress.org/page/journal/14602075/authorguide#dataavailability

2) Each figure legend must specify
- size of the scale bars that are mandatory for all micrograph panels
- the statistical test used to generate error bars and P-values
- the type error bars (e.g., S.E.M., S.D.)
- the number (n) and nature (biological or technical replicate) of independent experiments underlying each data point
- Figures may not include error bars for experiments with n<3; scatter plots showing individual data points should be used instead.

3) Revised manuscript text (including main tables, and figure legends for main and EV figures) has to be submitted as editable text file (e.g., .docx format). We encourage highlighting of changes (e.g., via text color) for the referees' reference.

4) Each main and each Expanded View (EV) figure should be uploaded as individual production-quality files (preferably in .eps,

.tif, .jpg formats). For suggestions on figure preparation/layout, please refer to our Figure Preparation Guidelines: http://bit.ly/EMBOPressFigurePreparationGuideline

5) Point-by-point response letters should include the original referee comments in full together with your detailed responses to them (and to specific editor requests if applicable), and also be uploaded as editable (e.g., .docx) text files.

6) Please complete our Author Checklist, and make sure that information entered into the checklist is also reflected in the manuscript; the checklist will be available to readers as part of the Review Process File. A download link is found at the top of our Guide to Authors: embopress.org/page/journal/14602075/authorguide

7) All authors listed as (co-)corresponding need to deposit, in their respective author profiles in our submission system, a unique ORCiD identifier linked to their name. Please see our Guide to Authors for detailed instructions.

8) Please note that supplementary information at EMBO Press has been superseded by the 'Expanded View' for inclusion of additional figures, tables, movies or datasets; with up to five EV Figures being typeset and directly accessible in the HTML version of the article. For details and guidance, please refer to: embopress.org/page/journal/14602075/authorguide#expandedview

9) Digital image enhancement is acceptable practice, as long as it accurately represents the original data and conforms to community standards. If a figure has been subjected to significant electronic manipulation, this must be clearly noted in the figure legend and/or the 'Materials and Methods' section. The editors reserve the right to request original versions of figures and the original images that were used to assemble the figure. Finally, we generally encourage uploading of numerical as well as gel/blot image source data; for details see: embopress.org/page/journal/14602075/authorguide#sourcedata

We realize that it is difficult to revise to a specific deadline. In the interest of protecting the conceptual advance provided by the work, we recommend a revision within 3 months (2nd Jan 2023). Please discuss the revision progress ahead of this time with the editor if you require more time to complete the revisions. Use the link below to submit your revision:

Link Not Available

***

Rev_Com_number: RC-2022-01537
New_manu_number: EMBOJ-2022-112630
Corr_author: Saurin
Title: A bifunctional kinase-phosphatase module integrates mitotic checkpoint and error-correction signalling to ensure mitotic fidelity

GENERAL STATEMENT (for all reviewers)

We thank all reviewers for providing very detailed and informative assessments of our manuscript. The comments they raised have helped us to significantly improve the manuscript after revision.

All reviewers were complementary about the data and the rigor of the study. Reviewers 2 and 3 commented on the significance of the work, with their assessment complementary, specifically about the fact that it bridges several previous studies and links these to kinase-phosphatase regulation on the BUB complex. We agree that this a major strength of the work. That is why we also believe the comment by reviewer 1 that "most of the phenotypes/observations are consistent with the literature and not surprising" is, in our opinion, a strength and not a weakness because it brings together various findings into one conceptual model. In this case, the concept that a dual kinase-phosphatase module integrates two major mitotic processes will, we believe, prove to be a significant breakthrough that helps to explain how these processes are properly integrated at kinetochores.

The main criticism of all reviewers was related to the interpretations, logical flow and writing style, which in general, we felt were valid comments. To improve these points, we have overhauled the writing and presentation of the data. In particular, we have been more careful with our interpretations, we have limited the amount of speculation, and importantly, we have improved the logical flow to better link the two halves of the paper together.

In terms of points requiring new experiments, there were 3 main points in total:

1) The role of the feedback loop - Reviewers 1 and 3 raised an important issue about the PLK1/PP2A feedback loop, which we addressed with new experiments to uncouple the feedback. These new experiments are presented in two new figures (Figure 3 and EV3).

2) The increase recruitment of BUB1-PLK1/PP2A when MELT numbers are increased - Reviewer 2 made an important point about KNL1/BUBs/PLK1/PP2A levels at kinetochores, including a good suggestion to perform FRAP analysis to examine BUB complex dynamics when MELT numbers are increased. We performed these experiments, and the data demonstrates reduced turnover over of the BUB complex when MELT numbers are increased, which may help to explain some of the observed phenotypes. This new data is now presented in Figure EV4

3) The link between MELT number and PLK1/PP2A levels - Reviewer 1 had a second major comment regarding the modulation of MELT number and how this cannot be directly linked to PLK1/PP2A levels and their effects on mitotic fidelity. We added new experiments to reply to this comment, shown in new Figures 5 and EV5. In addition, we have modified the text to include a detail description of the points we raise in our individual response to the reviewer.

There were some additional experiments requested by reviewer 1, which have also been addressed, and some further additions that we have chosen to include ourselves. For example, we have established a simple in cell protein-protein interaction assay based on dCas9 to examine protein-protein interactions away from the kinetochore.

All the other comments raised by the reviewers were textual points, which in most case we felt were valid, as outlined in our point-by-point responses below.

Reviewer #1 (Evidence, reproducibility and clarity (Required)):

The proper segregation of chromosomes during mitosis depends on correct kinetochore-microtubule (KT-MT) attachments which is monitored by the spindle assembly checkpoint (SAC). The Bub complex composed of the Bub1-Bub3 and BubR1-Bub3 complexes plays an important role in regulating KT-MT by recruiting Plk1 and PP2A-B56 as well as the SAC components Mad1/Mad2 and Cdc20. The Bub complex is recruited to kinetochores by Bub3 binding directly to phosphorylated MELT repeats on KNL1. The MELT repeats are predominantly phosphorylated by Mps1 but can also be phosphorylated by Plk1.

Here the authors investigate the Plk1 and PP2A-B56 module on BubR1 further. It is already known that Plk1 is recruited to BubR1 T620 (a Cdk1 site) and that this is important for proper chromosome segregation. Furthermore, BubR1 binds to PP2A-B56 through and LxxIxE motif that is phosphorylated by Plk1 to stimulate binding. The main message of the paper is that the Plk1-PP2A-B56 module on BubR1 is crucial for integrating SAC and KT-MT attachments and that a homeostatic negative feedback loop between Plk1 and PP2A-B56 exists to limit the levels of these enzymes. As outlined below I do not think that their experimental evidence/setup can be used to draw these conclusions. Overall, I have limited comments on the technical execution of experiments as this is overall done well but more on interpretations of results and what conclusions can be drawn. Also, most of the phenotypes/observations are consistent with the literature and not suprising.

We thank the reviewer for their comments. We have now performed extensive new experiments to address most of the concerns highlighted below, in particular, to address the role of feedback between PLK1 and PP2A and to better link the data on MELT numbers to PLK1/PP2A levels.

Major comments:

The authors generate a scenario where high levels of Plk1 are recruited to BubR1 by removing the entire C-terminus to remove the PP2A-B56 binding site and a situation of high PP2A-B56 by replacing the BubR1 C-term with B56gamma. Firstly, these are crude mutants generating extreme situations - specifically the fusion of B56gamma which likely creates artificially high levels of BubR1-B56gamma making it difficult to make conclusions on the physiological levels.

The fusion of B56 is admittedly less optimal than increasing PLK1 at its native phospho-binding site, but this does still lock the phosphatase-bound state in a set stoichiometry, crucially in the absence of kinase. This is required to assess direct phosphatase effects. These PLK1/PP2A levels may well be higher than observed physiologically when considering the behaviour of all BUBR1 molecules, since we doubt they ever reach 1:1 stoichiometry with either PLK1 or PP2A. However, the feedback loop is operating within individual molecules (figure 1), which may well individually flip between PLK1 or PP2A bound states. This may occur on certain molecules at specific times (as mentioned in the discussion). Therefore, locking the PLK1 or PP2A-bound state on all molecules is, in our opinion, still a valid and useful perturbation to assess function of these two states.

These mutants are then analysed in a number of assays and phenotypic consequences analysed in the presence of nocodazole + Mps1 to evaluate SAC strength. It would be interesting to see what the phenotypes are without Mps1 inhibition.

The sensitize SAC assay (nocodazole + MPSi) has been used by many groups (originally Santaguida et al, 2011; Saurin et al, 2011), because it reduces SAC signals from all unattached kinetochores which would otherwise produce a saturated response. In this case, we specifically chose a dose of MPS1 inhibitor that gave a partial SAC response from which we could observe strengthening or weakening – a key point of this assay. The only other way to do this, which has been used by some in the literature, is to use taxol or a low dose of nocodazole, which prevents all kinetochores from signalling to the SAC. However, this approach would make it difficult to untangle the effects on SAC and KT-MT attachment stability, and it was critical to do this in in our case. However, we have now repeated experiments in just nocodazole alone, as suggested, and we do still observe SAC weakening in the BUBR1-B56γ cells (Figure EV2G).

I cannot see in any way how the authors from two end points (high kinase or high phosphatase) can reach a conclusion that a homeostatic negative feedback loop exists between these enzymes that is critical for integrating SAC and KT-MT interactions.

We conclude that a negative feedback loop exists that balances PLK1 or PP2A levels on the BUB complex (Figure 1). We use mutants to disrupt this balance, and produce a kinase-dominant or phosphatase-dominant situation, which demonstrates that the respective processes become dysfunctional, as predicted (Figure 2). We do agree with the claim that this alone does not specifically link the feedback to the regulation of these processes, and we have now been careful not to conclude that from these data.

They can only conclude on what happens if you have too much kinase or phosphatase but no data are addressing what happens if you manipulate the cross-talk on BubR1. The experiments to do must be to carefully tune the proposed cross talk and then monitor what happens. This can be done by bypassing Plk1 regulation of the PP2A-B56 binding site or increasing the strength of the Plk1 binding site.

We thank the reviewer for raising this point. To address this, we have introduced a new set of BUBR1 mutants to impair the crosstalk between PLK1 and PP2A binding sites on BUBR1 (new Figures 3 and EV3). We created alanine mutants of the PLK1 sites (S676/T680A, $KARD^{2A}$), of the CDK1 site (S670A) and of both ($KARD^{3A}$) in the PP2A-binding motif to address the role of PLK1 (and CDK1) in controlling the feedback loop. We also mutated the PLK1 sites to phospho-mimicking aspartates (S676/T680D) to see if the aspartates recover the role of PLK1 in controlling the PP2A-binding motif. Our results showed that the feedback loop regulates the levels of the kinase/phosphatase module on BUBR1, with consequences on SAC strength and KT-MT attachments.

Furthermore, there is no data to show that cross-talk is changing in response to changes in KT-MT attachment status - is the levels and activities of Plk1/PP2A-B56 on BubR1 changing. Yes Plk1 and PP2A-B56 can regulate each other on BubR1 but is this regulated as proposed.

We observe a decrease in PLK1 and PP2A-B56 on metaphase kinetochores, compared to unattached kinetochores in nocodazole (Figure 7C), which is consistent with the reduced

BUBR1 observed in this situation (Figure 6A). Therefore, we suspect that the feedback between these enzymes is not changing upon KT-MT attachment.

My second comment relates to the fact that the two parts of the paper are not directly linked although the authors try to do this.

They nicely manipulate the MELT repeats on KNL1 to change the number of Bub complexes. However, they cannot directly link the data to changes in Plk1 and PP2A-B56 levels only as many other things are changing. By increasing MELT numbers Bub complex and Mad1/Mad2 levels increase as well as an example and this makes interpretations complicated. To me these experiments are not addressing the main conclusions of the paper.

There are two elements to this comment – the effect of modulating MELT number on SAC strength (and PLK1) or on KT-MT stability (and PP2A). We will discuss each separately:

For SAC regulation, we feel that the data is clear, and the interpretations are justified, although we have now added new data to support this point. Increasing MELT numbers causes defects in MELT-BUB dissociation (Figure 6) and SAC silencing (Figure 5 and EV5). Importantly, these phenotypes can be rescued by inhibiting PLK1 (Figures 5C, D and 6BE-G). So, we do link the effects of high MELT number to PLK1 activity. Our interpretation is that when MELT numbers are increased, the ability of PLK1 to phosphorylate these motifs and maintain the SAC platform (when MPS1 is inhibited pharmacologically or upon KT-MT attachment) is also enhanced. So, whilst it is true that many factors, such as the kinetochore levels of BUB/MAD1/MAD2, are important for the SAC, the ability of PLK1 to maintain BUB levels (via pMELT) is crucial for the improved SAC response and that changes as MELT number increases.

Regarding the other effects of increasing the MELTs, we do not observe increased MAD1 levels (Figure 6D). We have now also evaluated the levels of phosphorylated MAD1-pT716, which is important for MCC assembly, and our data show that WT and 19xMELT exhibit similar MAD1-pT716 levels during a nocodazole arrest and following MPS1 inhibition (Figure 5E). BUB1 levels clearly do increase at kinetochores when MELT numbers are increased, and we have now also explored the possibility that increased BUB1 activity could contribute to SAC strengthening in this situation (for example, by enhancing Aurora B recruitment to centromeres). Our new data shows that BUB1 inhibition does not alter SAC strength or MELT dephosphorylation kinetics in the 19xMELT (Figure EV5B,C). In summary, the main changes we observe are elevated BUB1 levels due to MELT phosphorylation, and increased BUB1 phosphorylation on pT461 (new data in Figure 5F). All this points towards a localized effect of PLK1 on/around the BUB complex, consistent with the ability of PLK1 inhibition to reverse these effects and weaken the SAC.

We have now been more careful in interpreting all of these data in the new manuscript. Our analysis of SAC silencing upon KT-MT attachment, demonstrates that preservation of the BUB complex on metaphase kinetochores in the 19xMELT is not sufficient to maintain the SAC (new figure 6), but it is associated with defects in KT-MT regulation (figure 7).

For KT-MT attachment regulation, we agree that we do not have a similar way to inhibit PP2A-B56 activity to rescue hyperstable microtubule attachment when MELT numbers are high. For this, we require a rapidly inducible way to inhibit PP2A-B56 activity after

attachments have formed. Something that is not technical feasible at the present. We can also not say for certain that reduced MELT numbers destabilizes microtubule due to lack of PP2A, however we feel this is the most like interpretation for the following reasons. The phenotype of removing PP2A from BUBR1 or removing the MELT from KNL1 (along with all associated factors) on fixed chromosome alignment assays appears to be identical: mutant cells have comparable chromosome misalignment due to unattached kinetochores (compare Figures 2G and 3J with Figure 7B), and in addition, KT-MT stability is very similar between the BUBR1$^{\Delta KARD}$ and KNL1$^{\Delta MELT}$ (Compare Figure 2H with Figure 7E). Therefore, the additional factors lost by removing the MELTs cannot be having such a strong impact in KT-MT attachments. The obvious factor that could affect attachment strength is again BUB1, via Aurora B recruitment to centromeres. However, loss of BUB1 (after MELT removal) is predicted to enhance attachment stability and not decrease it, as we observe. So, whilst we cannot conclude that modulating MELT number affects attachment stability via PP2A, we feel that this is the most likely explanation. We have carefully explained these points and been more careful when interpretating this data in the new version.

Specific comments:

1) I would caution the interpretation of phenotypes being suppressed by Plk1 inhibitors. This does not address whether it is BubR1 bound Plk1 that is specifically affected - several Plk1 substrates could be contributing. Similar for Mad1 phosphorylation they cannot conclude that it is Plk1 bound to BubR1 phosphorylating Mad1.

We thank the reviewer for the suggestion, we have added data to support this at revision and we have now also been more careful in interpreting these data. Regarding MAD1 phosphorylation, we believe the reviewer confused MAD1 for BUB1, since we did not show any phospho-MAD1 data in the original version. We did show that BUB1-pT461 was reduced by PLK1 inhibition in BUBR1$^{\Delta PP2A}$ cells, and we have now added further data to show a similar reduction by mutation of the PLK1-binding site on BUBR1 (T620A; Figure 5G). This demonstrates that it is the localised PLK1 at BUBR1 that is responsible for BUB1 phosphorylation, therefore this has now been incorporated into the final model.

2) On page 4 the authors write: "We sought to modulate MELT numbers in a way that would allow BUB complex levels to be increased or decreased in a graded manner, thereby causing reciprocal changes to PLK1 and PP2A levels." I do not see how this will result in reciprocal levels as total Bub complex levels are increased.

We realised the usage of the word “reciprocal” was not correct in this sentence. We replaced it with “respective”, to highlight our expectation of a positive correlation between BUBs/PLK1/PP2A and the number of MELT motifs.

3) Page 7 first paragraph authors write: "remained high despite inhibition of Mps1". This is not completely correct as levels are dropping dramatically after 5/10 min of Mps1 inhibition. Total drop seems more than WT situation.

We agree the sentence needs rephrasing, although MPS1 inhibition does cause a much stronger decrease in BUB1 and pMELT in WT cells when compared to 19xMELT (see

Figures 5B and EV5A). We have now rephrased this sentence to more accurately reflect the changes shown in the figure.

4) Page 7 second paragraph authors write: "implying the elevated PLK1 in this situation (Figure 3E) is also able to better amplify MELT signalling..." This cannot be concluded as the starting levels are so much higher in 19XMELT than WT and there is no data to show this is due to more Plk1 bound to BuBR1.

We have removed this sentence in the new manuscript, and we have been more careful when discussing MELT amplification by PLK1

5) Page 7 second section authors write: "Figure 5B shows that KNL1deltaMELT causes severe chromosome misalignments, as expected, given the PP2A-B56 binding to KNL1 is inhibited in this situation". Multiple things are changing so this cannot be interpreted only as a readout of PP2A-B56. With no MELTs there is no recruitment of SAC proteins.

We appreciate that many things are changing as well as PP2A-B56, and we have now been more careful in our interpretations, but it is important to acknowledge that the data on chromosome misalignments and KT-MT stability is actually remarkably similar between the BUBR1$^{\Delta PP2A}$ and KNL1$^{\Delta MELT}$ situation (as outlined above). Whilst this is still correlation rather than causation, it does imply that the common factor that leads to these phenotypes is the loss of PP2A-B56 in both cases. There are clearly additional effects in the KNL1$^{\Delta MELT}$ situation that could contribute to the phenotype, but the most obvious changes that have been linked to KT-MT stability are the lack of BUB activity which is predicted to help stabilise attachments by reducing Aurora B (something that we do not observe based on assay in Fig.7E).

6) Page 7 second section authors write: "Therefore, the number of MELT motifs is crucial for determining the stability of KT-MT attachments, most probably by setting the levels of PP2A-B56..." Again many things are changing so impossible to interpret data in light of PP2A-B56.

See relevant points above

7) One possibility not mentioned by authors is that PP1 bound to KNL1 cannot act as efficiently on some MELT repeats and that the dephosphorylation by PP1 of the 19xMELT is different from KNL1 WT which would impact on their results.

Previous data from our lab do not support this hypothesis. In Cordeiro et al. 2020 we show quite carefully that the main contribution of PP1/PP2A to MELT dephosphorylation is the removal of PLK1. We could not find any evidence of MELT dephosphorylation by PP1 and PP2A, because treatment with Calyculin A did not affect MELT dephosphorylation kinetics when PLK1 was removed from the BUB complex.

Reviewer #1 (Significance (Required)):

See above.

Reviewer #2 (Evidence, reproducibility and clarity (Required)):

In this manuscript by Corno et al the authors investigate how the kinase/phosphatase balance is regulated at kinetochores during mitosis. When kinetochores are unattached, mitotic kinases signal through the spindle assembly checkpoint (SAC) to prevent progression through mitosis. Once attachments have occurred, the activities of kinetochore phosphatases silence the SAC and stabilize kinetochore-microtubule attachments to promote exit from mitosis. Key regulators of this signaling are the BUB proteins Bub1 and BubR1, which recruit the kinase Plk1. BubR1 also recruits the phosphatase PP2A-B56 in a Plk1-dependent manner. By analyzing the recruitment of Plk1 and PP2A-B56 at unattached kinetochores, the authors uncovered a mechanism by which Plk1 and PP2A-B56 negatively regulate each other's recruitment, thereby maintaining an optimal kinase/phosphatase balance. Disrupting this balance can lead to either a hyperactive SAC or premature stabilization of erroneous attachments.
Overall, this is a very careful study and the data is clear and consistent. My only comments are in regards to the interpretation of the Knl1 data on the manuscript.

Comments

- In figures 3 and 4, the authors engineer a system to manipulate the levels of BUBs at the kinetochore by modulating the number of MELT repeats on Knl1. For this, they mutate all 19 MELT repeats on Knl1 and then add back discreet number of MELT motifs that follow a consensus sequence. They find that a 6x mutant, which contains 6 repeats of this consensus MELT motif, is sufficient to rescue the functions of Knl1 on the SAC and on chromosome alignment. However, 12x and 19x MELT repeat versions show an increased SAC response and increased stability of kinetochore-microtubule attachments. The authors interpret this as a result of increased kinetochore levels of BUBs, and therefore, increased levels of both Plk1 and PP2A-B56 (Fig. 3). However, from their representative images in Fig. S3A, it does not appear as if BUB levels are significantly increased in cells expressing the 12x and 19x Knl1 mutants, compared to wild-type controls. Considering that in the 12x and 19x mutants Knl1 recruitment is reduced by more than half of controls (Fig. S3B) and because the authors normalized their BUB kinetochore intensity levels by the Knl1 values, it makes it seem as if BUB kinetochore levels are increased in the cell under these conditions. While in the 12x and 19x mutant conditions there are more molecules of BUBs per Knl1, the overall BUB levels are the same as in wild-type controls. Since the MELT repeat used throughout the paper is a consensus sequence that is likely optimal for BUB binding, it is possible that the phenotypes of the 12x and 19x mutants are explained because of an increase in the affinity of BUBs for Knl1 rather than overall levels. This would also help explain why Knl1 and BUBs are observed at the spindle midzone in the 19x mutant (Fig. S4). To distinguish between these possibilities, the authors might consider doing FRAP experiments using fluorescently labelled Bub1 or BubR1 and measure BUB protein dynamics at kinetochores.

This is a good point, and whilst we did state in our original paper that BUB/PLK1/PP2A levels were “enhanced at KNL1” rather than “enhanced at kinetochores”, we have now expanded on this point and performed new experiments and analysis to address it – this now forms the new figure EV4. As part of this new analysis, we measured turnover rates of

BUB1, BUBR1 and KNL1 to/from kinetochores by FRAP, as suggested. This demonstrates slower turnover of BUB1 and BUBR1 in the 19xMELT situation. We believe this is due to the number of the active MELTs – and not to the consensus sequence we used – because wild type KNL1 is estimated to use 6-7 active MELTs (Vleugel et al 2015) and our 6xMELT cells displayed similar BUB1/BUBR1 turnover kinetics to WT Knl1 cells.

- Also in regards to the 12x and 19x mutants, because these reduce Knl1 kinetochore levels, this fact alone might explain some of the observed phenotypes, such as the mild defects in chromosome alignment (Fig. S5).

We feel this is unlikely because knockdown of KNL1 causes hypostable KT-MT attachments (Vleugel et al. 2013), whereas the 19xMELT cause hyperstable KT-MT attachments (Figure 7E), consistent with the predicted effect of enhancing PP2A levels.

Reviewer #2 (Significance (Required)):

This is a significant advancement in our understanding of how the spindle assembly checkpoint is regulated by kinases and phosphatases at the kinetochore. The authors used very precise manipulations to dissect how these components are balanced and they uncovered a very interesting negative feedback mechanism. They also provided significant evidence for the importance of this balance in normal mitotic progression. This work will be of broad interest to cell biologists, as well as cancer biologists. My expertise is on the fields of cell biology, mitosis and cell division mechanisms.

Reviewer #3 (Evidence, reproducibility and clarity (Required)):

A dividing cell relies on both error correction and spindle assembly checkpoints (SACs) to ensure accurate chromosome segregation. The PLK1-PP2A pair resides at the interface of the two pathways and makes these pathways robust and responsive. By generating fusion proteins and mutagenesis to perturb PLK1 and PP2A activities in HeLa cells, the authors found that both enzymes constitute a negative feedback loop on the scaffold BubR1. The docked PLK1 phosphorylates the PP2A binding site to recruit PP2A, while the PLK1 docking site is dephosphorylated after PP2A recruitment. They also manipulate kinetochore recruitment of PP2A to show effects on the SAC and on kinetochore-microtubule attachments. Independently, by varying active MELT repeats on KNL1, the authors found that decreasing MELT repeats weakens the SAC with unstable attachments, while increasing MELT repeats strengthens the SACs with hyperstable attachments. Thus, the recruited level of BubR1 determines SAC strength and attachment stability. The data included in this paper are solid, and the concepts are interesting, but these potential strengths of the manuscript are obscured because the logical progression and presentation are difficult to follow.

We thank the reviewer for taking the time to carefully read the paper and for making some important points about how the manuscript was written and presented. It can often take more effort to criticise presentation style, because it is not easy to precisely pinpoint all the issues, but in this case, we genuinely appreciate these efforts. We agreed with all of the points raised, and we have essentially re-wrote the manuscript to address them, including a new abstract, more focussed introduction, a modified results section that includes better linking between the first and second sections, and a more focussed discussion. We think these changes have great improved the flow and logic of the manuscript.

Major comments:

1. The first three paragraphs of the introduction lead up to the question: "Why the same phosphatase complex is used for both process is still not clearly understood". One conceptually simple answer is that the SAC should be silenced as attachments are stabilized, so it makes sense to use the same enzyme to accomplish both tasks. If the authors have something else in mind, they should clarify.

We have removed this statement in the new introduction.

2. The argument linking the negative feedback loop to biological functions is not straightforward. The authors provide evidence in Figure 1 for regulatory pathways between docked PLK1 and bound PP2A. However, their assays in Figure 2 bypass the feedback loop by directly modulating PP2A activity. These experiment supports an argument that the kinase/phosphatase activity balance is important, but do not address the feedback loop specifically (which could potentially be done using mutations that disrupt the feedback regulation). The claim that "a homeostatic feedback loop maintains an optimal balance of PLK1 and PP2A on the BUB complex" is too strong because there is no direct evidence connecting the feedback loop to optimal function.

We thank the reviewer for raising this point. To address this, we have introduced a new set of BUBR1 mutants to impair the crosstalk between PLK1 and PP2A binding sites on BUBR1 (new Figures 3 and EV3). We created alanine mutants of the PLK1 sites (S676/T680A, $KARD^{2A}$), of the CDK1 site (S670A) and of both ($KARD^{3A}$) in the PP2A-binding motif to address the role of PLK1 (and CDK1) in controlling the feedback loop. We also mutated the PLK1 sites to phospho-mimicking aspartates (S676/T680D) to see if the aspartates recover the role of PLK1 in controlling the PP2A-binding motif. Our results showed that the feedback loop regulates the levels of the kinase/phosphatase module on BUBR1, with consequences on SAC strength and KT-MT attachments.

3. The paragraph starting with "Given the roles of PLK1 and PP2A ...": How does the kinase-dominant situation destabilize KT-MT attachments? Is it by inhibiting PP2A or by phosphorylating some kinetochore components? The former pathway is unclear because the authors show that PLK1 promotes PP2A recruitment in Figure 1.

The relevant KT-MT attachment phenotype in the "kinase dominant" situation is associated with (i) high PLK1 levels, (ii) loss/reduced levels of PP2A and (iii) increased levels of Hec1-pS55 (new data Figure 3I and EV2H). The increased Hec1 phosphorylation is likely to contribute to the unstable KT-MT attachments we observed. Our experiments do not distinguish whether PP2A directly controls Hec1-pS55 levels, or whether this occurs indirectly (for instance, due to enhanced PLK1), or both. We have been careful not to conclude direct effects of PP2A on pHEC1, although we believe that is the simplest interpretation.

4. The paragraph starting with "In summary, a balanced recruitment of PLK1 and PP2A ...": What is meant by "phosphorylation sites that block KT-MT attachment" (used twice in the paragraph)? Block means prevent binding, as opposed to activities that destabilize attachment by promoting unbinding. Which do the authors mean? The following is also unclear: "kinetochores are no longer responsive to MT attachment". If attachment is blocked, as stated in the previous sentence, then what does it mean to say that kinetochores are not responsive to attachment (which never occurred if it was blocked)?

These statements are indeed ambiguous, and we have removed them in the new manuscript.

5. Figures 1-2 and Figures 3-5 are separate concepts, but this is never explained clearly in the manuscript. Specifically, Figures 1-2 focus on the antagonism between PLK1 and PP2A activities, whereas Figures 3-5 focus on changing both activities in the same direction (either increase or decrease). There is no transition from one part to the other. Both concepts should be explained and the hypotheses stated clearly.

We thank the reviewer for pointing this out. We have now more carefully linked the two parts of the paper.

6. Several words are used ambiguously. "Homeostasis" is vague, and it is unclear what exactly the authors mean. As discussed above, the meaning seems different for figures 1-2 vs figures 3-5. "Reciprocal changes" is also unclear (and seems misleading) because the

perturbations of PLK1 and PP2A levels are in the same direction (more MELT motifs means more binding sites for both). For "preserved" (description of figure 4F), it's unclear if the authors refer to the Bub1 and BubR1 levels at metaphase or the change between prometaphase and metaphase. The authors should clarify what they mean and how it is measured.

We thank the reviewer for pointing these issues out. We have removed homeostasis from the manuscript. The word “reciprocal” was incorrect, and has now been changed to “respective”. For “preserved”, we refer to the fact that BUB1 and BUBR1 are reduced less on attached kinetochore in metaphase, in comparison to unattached kinetochores in prometaphase. We have clarified these points, which are now illustrated in Figure 6A.

7. "too much kinase-phosphatase module would cause a strong SAC and hyperstable KT-MT attachment, and too little would cause a weak SAC and hypostable KT-MT attachments": The reasoning behind these predictions is not clear. For example, why does high phosphatase not silence the SAC as explained earlier in the manuscript?

We have now removed this prediction from the text and attempted to clearly explain all stated hypotheses.

8. The mitotic exit assay in Figure 4G is hard to interpret. Mitotic duration depends on establishing correct attachments and then silencing the SAC. 19xMELT could affect both. A better measurement of SAC silencing would be time from metaphase alignment to anaphase.

We thank the reviewers for the comment. We moved the graph to Figure 6C, and we plotted the time from metaphase alignment to anaphase as suggested. The data shows 19xMELT cells do not experience an increase in metaphase duration, despite the high levels of the BUB complex being recruited at kinetochores.

9. The paragraph starting "In summary, PLK1 is able to phosphorylate MELTs to recruit Bub1": The authors should clarify what was already known and what advance they are making. Similarly, the sentence starting with "Therefore, the number of MELT motifs ..." should also clarify the advance relative to previous findings.

We have clarified these points in the new manuscript

Minor comments:

1. Please include pages numbers (and line numbers are also helpful).

The manuscript now includes line and pages numbers.

2. Previous literature (PMID 17785528) suggests that phosphorylation of pT620 is important for KT-MT regulation but not SAC signaling. Can the authors comment on this?

In Cordeiro et al (2020), we observed a SAC defect in BUBR1-T620A mutants, but only using a sensitized SAC assay. This assay was not employed in the Elowe et al (2007) study, which may explain the lack of a SAC phenotype.

3. SAC silencing seems more appropriate than "mitotic exit" in the last sentence of the second paragraph.

We thank the reviewer for point this out. We were indeed referring to SAC silencing in this sentence, since this occurs within seconds after the last attachment, as opposed to mitotic exit which takes minutes. This has now been corrected.

4. Figure 1 would be clearer with images and the relevant quantifications together in the same panel.

We thank the reviewer for the suggestion. Figure 1 has been updated accordingly.

5. In the first paragraph of Results, the authors primarily explain the impacts of PP2A on SAC silencing but not on KT-MT attachment, even though the topic sentence seems equally weighted to both.

We have now removed this paragraph at the start of the results and integrated it in the introduction to properly explain the effects of PP2A on SAC and KT-MT attachments.

6. Some terms are not defined when first introduced, such as the KARD domain and B56gamma.

These terms are now properly defined in the introduction.

7. Figure 1JK: how were mitotic cells enriched and harvested?

Cells were arrested with thymidine 2mM for 24h, then released in nocodazole 3.3µM for 16h to enrich mitotic cells. Doxycycline 1 µg/ml was used to induce the expression of the BUBR1 constructs during and following the thymidine block. Mitotic cells were then harvested by a mitotic shake-off. We thank the reviewer for pointing out this missing information, which is now included in Material and Methods. We have also carefully checked that all other key experimental information is included in the figures, legends and methods.

8. Figure 2C: a log scale may help show changes in both directions.

We thank the reviewer for the suggestion. Figure 2C has been updated accordingly.

9. Chromosome alignment assays in Figure 2 are not so informative because perturbation either way can generate misaligned chromosomes. The primary figures are dense with data, so these results can be made supplemental.

The chromosome alignment assays from live-cell have been moved to Figure EV2.

10. Figure 3F: can the authors comment on the B56gamma decrease between nocodazole and MG132 conditions?

Given the fact that BUBR1 constitutes a platform for B56γ recruitment at kinetochores (Vallardi et al, 2019), we predict that metaphase cells experience a drop in B56γ because BUBR1 levels also decrease in this situation (as shown in Figure 6A). In addition, BUBR1-pS676 (Elowe et al. 2007) and pS670 (Huang et al. 2008) decrease at metaphase kinetochores in comparison to prometaphase cells. We believe these changes could explain the decrease in B56γ levels in attached (MG132) versus unattached (nocodazole) kinetochores.

11. Figure 4A: the data are difficult to interpret as presented. It is not clear whether SAC signaling changes monotonically with number of MELT repeats. It would be better to plot MELT number vs a summary statistic (such as time to 50% mitotic exit or something else that the authors find informative).

We thank the reviewer for the suggestion. To allow a better interpretation of this type of experiment (like in Figures EV2G, 3H, EV3J and 5A), we replaced the cumulative distributions with violin plots showing the individual mitotic timings in the presence of nocodazole +/- MPS1i +/- BUB1i or PLK1i. Cells that did not exit from mitosis before the end of the movie were marked.

12. Figure 6. "uncouplr" should be "uncouple".

This sentence has been removed from the new version

13. Missing references in bibliography: Ghongane et al 2014, Roy et al. 2020.

These references are included in the new version.

Reviewer #3 (Significance (Required)):

The proposed model is conceptually significant, and this mechanistic work bridges several previous findings. Biochemical studies suggest that KNL1 harbors PLK1-PP2A and Bub complex, and functional studies suggest that truncation on MELT motifs generates mitotic errors (PMID 24363448, 24344183). Furthermore, biochemical and functional assays suggest that PLK1 is a key regulator of the SAC (PMID 33125045). In this work, the authors link functional consequences to biochemical interactions among KNL1, BubR1, PLK1, and PP2A, which is an advance.

This work should appeal to the kinetochore and cell cycle communities, but the logical flow needs to be improved.

My most relevant expertise is in mitotic kinases and regulation of kinetochore microtubules.

Dr. Adrian T Saurin
University of Dundee
Division of Cellular Medicine
Jacqui Wood Cancer Centre
Ninewells Hospital and Medical School
Dundee DD1 9SY
United Kingdom

7th Jul 2023

Re: EMBOJ-2022-112630R
A bifunctional kinase-phosphatase module integrates mitotic checkpoint and error-correction signalling

Dear Adrian,

Thank you again for submitting your revised manuscript for our consideration. Two of the original Review Commons referees have now assessed it once more, and are mostly satisfied with the revisions and improvements of the manuscript. As you will see from the comments below, they only retain some minor questions that should be answered via modifications to the text/discussions, during a final round of minor revision.

At this stage, please also make sure to fully address a number of outstanding editorial concerns:

- Pre-acceptance checks by our data editors have raised several queries with the data descriptors in the figure legends, which you will find as comments in the attached edited/commented Word document with activated "Track changes" option. I would appreciate if you incorporated the requested final text modifications and answered the Figure legend queries directly in this version (and modified figures where necessary), uploading the edited main text document upon resubmission with changes/additions still highlighted via the "Track changes" option, to facilitate our final checking.

- Please provide the accession ID or link for the used dataset. It is insufficient to read: "The dataset from (Tromer et al., 2016) was used... " -> please also consult the section dedicated to DATA CITATION in our Guide to Authors: https://www.embopress.org/page/journal/14602075/authorguide#referencesformat

- Please upload each of the main and EV Figures as individual files, with sufficient resolution/quality for production. The legends should remain in the manuscript.

- Please remove the Disclosure and competing interests statement from the "Acknowledgement" section, and provide a separate section "Disclosure and competing interests statement" (next to the Data Availability section) - for details, see https://www.embopress.org/competing-interests

- As we are switching from a free-text author contribution statement towards a more formal statement based on Contributor Role Taxonomy (CRediT) terms, please remove the present Author Contribution section and instead specify each author's contribution(s) directly in the Author Information page of our submission system during upload of the final manuscript. See https://casrai.org/credit/ for more information.

- Please add the following missing callouts: Fig. EV1A-H, EV2G, Appendix Figure S3A-B

- Please start the Appendix PDF with a title page containing a brief Table of Contents (listing all the Appendix figures) with page numbers.

- Movie files should be uploaded individually and (as Expanded View Movies) renamed to "Movie EV1/2/3" with the corresponding in-text callouts. Their legends should be in separate text files, each of them zipped with the corresponding movie file before uploading.

- Please correct Section order: It should be: title page with complete author information, abstract, introduction, results, discussion, materials & methods, data availability section, acknowledgements, disclosure and competing interests statement, references, main figure legends, tables, expanded figure legends.

- Please enter a valid email address for co-author Elena L Harrington into our online system, as the last acknowledgement message sent by out office could not be delivered to e.l.harrington@dundee.ac.uk.

- Finally, please provide suggestions for a short 'blurb' text prefacing and summing up the conceptual aspect of the study in two

sentences (max. 250 characters), followed by 3-5 one-sentence 'bullet points' with brief factual statements of key results of the paper; they will form the basis of an editor-written 'Synopsis' accompanying the online version of the article. Please also upload a synopsis image, which can be used as a "visual title" for the synopsis section of your paper. The image should be in PNG or JPG format, and please make sure that it remains in the modest dimensions of (exactly) 550 pixels wide and 300-600 pixels high.

I am therefore returning the manuscript to you for a final round of minor revision, to allow you to make these adjustments and upload all modified files. Once we will have received them, we should be ready to swiftly proceed with formal acceptance and production of the manuscript.

With kind regards,

Hartmut

Hartmut Vodermaier, PhD
Senior Editor, The EMBO Journal
h.vodermaier@embojournal.org

*** PLEASE NOTE: All revised manuscript are subject to initial checks for completeness and adherence to our formatting guidelines. Revisions may be returned to the authors and delayed in their editorial re-evaluation if they fail to comply to the following requirements (see also our Guide to Authors for further information):

1) Every manuscript requires a Data Availability section (even if only stating that no deposited datasets are included). Primary datasets or computer code produced in the current study have to be deposited in appropriate public repositories prior to resubmission, and reviewer access details provided in case that public access is not yet allowed. Further information: embopress.org/page/journal/14602075/authorguide#dataavailability

2) Each figure legend must specify
- size of the scale bars that are mandatory for all micrograph panels
- the statistical test used to generate error bars and P-values
- the type error bars (e.g., S.E.M., S.D.)
- the number (n) and nature (biological or technical replicate) of independent experiments underlying each data point
- Figures may not include error bars for experiments with n<3; scatter plots showing individual data points should be used instead.

3) Revised manuscript text (including main tables, and figure legends for main and EV figures) has to be submitted as editable text file (e.g., .docx format). We encourage highlighting of changes (e.g., via text color) for the referees' reference.

4) Each main and each Expanded View (EV) figure should be uploaded as individual production-quality files (preferably in .eps, .tif, .jpg formats). For suggestions on figure preparation/layout, please refer to our Figure Preparation Guidelines: http://bit.ly/EMBOPressFigurePreparationGuideline

5) Point-by-point response letters should include the original referee comments in full together with your detailed responses to them (and to specific editor requests if applicable), and also be uploaded as editable (e.g., .docx) text files.

6) Please complete our Author Checklist, and make sure that information entered into the checklist is also reflected in the manuscript; the checklist will be available to readers as part of the Review Process File. A download link is found at the top of our Guide to Authors: embopress.org/page/journal/14602075/authorguide

7) All authors listed as (co-)corresponding need to deposit, in their respective author profiles in our submission system, a unique ORCiD identifier linked to their name. Please see our Guide to Authors for detailed instructions.

8) Please note that supplementary information at EMBO Press has been superseded by the 'Expanded View' for inclusion of additional figures, tables, movies or datasets; with up to five EV Figures being typeset and directly accessible in the HTML version of the article. For details and guidance, please refer to: embopress.org/page/journal/14602075/authorguide#expandedview

9) Digital image enhancement is acceptable practice, as long as it accurately represents the original data and conforms to community standards. If a figure has been subjected to significant electronic manipulation, this must be clearly noted in the figure legend and/or the 'Materials and Methods' section. The editors reserve the right to request original versions of figures and the original images that were used to assemble the figure. Finally, we generally encourage uploading of numerical as well as gel/blot

image source data; for details see: embopress.org/page/journal/14602075/authorguide#sourcedata

At EMBO Press, we ask authors to provide source data for the main manuscript figures. Our source data coordinator will contact you to discuss which figure panels we would need source data for and will also provide you with helpful tips on how to upload and organize the files.

Further information is available in our Guide For Authors:
https://www.embopress.org/page/journal/14602075/authorguide

In the interest of ensuring the conceptual advance provided by the work, we recommend submitting a revision within 3 months (5th Oct 2023). Please discuss the revision progress ahead of this time with the editor if you require more time to complete the revisions. Use the link below to submit your revision:

Link Not Available

Referee #1:

The authors has addressed some of my concerns and that of the other reviewers. I think the reading and logic of the manuscript has improved a lot and the authors are more careful in their interpretations. One thing I am missing from the discussion and model is why Cdk1 activity is integrated by the KARD - is this Cdk1 associated with Mad1? I am not asking for more expriments but a comment/thought in the discussion also since they see a more strong effect of BubR1 S670A mutation vs BubR1 S676/T680 (Fig. 3). In the discussion the authors suggest that the PLK1-PP2A module was acquired in metazoans - do we know for sure that this is not used in yeast? There is a putative PP2A-B56 binding motif in MAD3 which to my knowledge has not been tested.

A few things:
There is no statistical analysis of many of the experiments and sometimes this would be important to know. As an example looking at data in Fig. 3 I would like to know if there is a difference between KARD 2A and S670 in several of the experiments.

Few errors I spotted:
Page 6 line 132 - that should be the
Page 18 line 456 - C.elegans

Referee #3:

The authors have rewritten the manuscript to improve presentation and clarity. They also added important new data directly addressing the feedback regulation (KARD-2A mutant), which have further strengthened the manuscript. My only comment is that it is not clear why the Cdk1 site (included in the KARD-3A mutant) is considered part of the feedback loop. No additional experiments are necessary, but I suggest that the authors discuss how they interpret the Cdk1 site (BubR1-S670) in the context of the feedback loop that is the focus of this section (beginning on line 183).

Referee #1:

The authors has addressed some of my concerns and that of the other reviewers. I think the reading and logic of the manuscript has improved a lot and the authors are more careful in their interpretations. One thing I am missing from the discussion and model is why Cdk1 activity is integrated by the KARD - is this Cdk1 associated with Mad1? I am not asking for more expriments but a comment/thought in the discussion also since they see a more strong effect of BubR1 S670A mutation vs BubR1 S676/T680 (Fig. 3).

This is an interesting point and we have now discussed the key role of CDK1 in initiating the feedback, by recruiting both PLK1 and PP2A, and we have added further discussion about the possibility that localised CDK1 restricts the feedback to kinetochores or to specific phases of mitosis. It will be important to test these possibilities in future.

In the discussion the authors suggest that the PLK1-PP2A module was acquired in metazoans - do we know for sure that this is not used in yeast? There is a putative PP2A-B56 binding motif in MAD3 which to my knowledge has not been tested.

Figure S8 in Cordeiro et al (2020) depicts the putative PP2A-B56 and PLK1 binding domains in a large number of eukaryotic MADBUB homologues. Although there are indeed putative PP2A-B56 binding motifs in some fungal species, these are rarely present together with a PLK1 binding region, unlike in metazoa. An exception is actually BUB1 in *Saccharomyces cerevisiae,* which has a putative co-localised PP2A and PLK1-binding motifs. However, in this case the PP2A-B56 motif precedes the PLK1 motif, which is very rarely observed in metazoa. Therefore, we feel that the PLK1 binding module has most likely been acquired in metazoa, although we cannot rule out a role in some fungal species.

A few things:

There is no statistical analysis of many of the experiments and sometimes this would be important to know. As an example looking at data in Fig. 3 I would like to know if there is a difference between KARD 2A and S670 in several of the experiments.

Most of the experiments are visualised using a Violin Plot. This allows the spread of data to be accurately visualised along with the 95% confidence intervals (thick vertical bars) calculated around the median (thin horizontal lines). We chose this format because it allows easy statistical comparison between any treatments and timepoints by eye, since when the vertical bar of one condition does not overlap with the bar in another condition the difference between the medians is considered statistically significant (at $p<0.05$). We have now included a line to state this in the main text because it is a key point that could otherwise easily be missed by the reader.

Few errors I spotted:

Page 6 line 132 - that should be the

Page 18 line 456 - C.elegans

We thank the reviewer for highlighting the typos which have now been corrected.

Referee #3:

The authors have rewritten the manuscript to improve presentation and clarity. They also added important new data directly addressing the feedback regulation (KARD-2A mutant), which have further strengthened the manuscript. My only comment is that it is not clear why the Cdk1 site (included in the KARD-3A mutant) is considered part of the feedback loop. No additional experiments are necessary, but I suggest that the authors discuss how they interpret the Cdk1 site (BubR1-S670) in the context of the feedback loop that is the focus of this section (beginning on line 183).

We have now mentioned how CDK1 initiates the feedback by recruiting both PLK1 and PP2A, and we have discussed how this could be used to limit the feedback to specific subcellular localisations or mitotic phases.

Dr. Adrian T Saurin
University of Dundee
Division of Cellular Medicine
Jacqui Wood Cancer Centre
Ninewells Hospital and Medical School
Dundee DD1 9SY
United Kingdom

28th Aug 2023

Re: EMBOJ-2022-112630R1
A bifunctional kinase-phosphatase module balances mitotic checkpoint strength and microtubule attachment dynamics

Dear Dr. Saurin,

Thank you for submitting your final revised manuscript for our consideration. I am pleased to inform you that we have now accepted it for publication in The EMBO Journal.

Your article will be processed for publication in The EMBO Journal by EMBO Press and Wiley, who will contact you with further information regarding production/publication procedures and license requirements. You will also be provided with page proofs after copy-editing and typesetting of main manuscript and expanded view figure files.

Should you be planning a Press Release on your article, please get in contact with embojournal@wiley.com as early as possible, in order to coordinate publication and release dates.

Thank you again for this contribution to The EMBO Journal and congratulations on a successful publication! Please consider us again in the future for your most exciting work.

Yours sincerely,

Hartmut Vodermaier

Hartmut Vodermaier, PhD
Senior Editor, The EMBO Journal
h.vodermaier@embojournal.org

Please note that it is EMBO Journal policy for the transcript of the editorial process (containing referee reports and your response letter) to be published as an online supplement to each paper. If you do NOT want this, you will need to inform the Editorial Office via email immediately. More information is available here:
https://www.embopress.org/page/journal/14602075/authorguide#transparentprocess

EMBO Press Author Checklist

Corresponding Author Name: Adrian T Saurin	
Journal Submitted to: EMBO J	
Manuscript Number: EMBOJ-2022-112630R	

USEFUL LINKS FOR COMPLETING THIS FORM
The EMBO Journal - Author Guidelines
EMBO Reports - Author Guidelines
Molecular Systems Biology - Author Guidelines
EMBO Molecular Medicine - Author Guidelines

Reporting Checklist for Life Science Articles (updated January
This checklist is adapted from Materials Design Analysis Reporting (MDAR) Checklist for Authors. MDAR establishes a minimum set of requirements in transparent reporting in the life sciences (see Statement of Task: 10.31222/osf.io/9sm4x). Please follow the journal's guidelines in preparing your
Please note that a copy of this checklist will be published alongside your article.

Abridged guidelines for figures
1. Data
The data shown in figures should satisfy the following conditions:
- ➞ the data were obtained and processed according to the field's best practice and are presented to reflect the results of the experiments in an accurate and unbiased manner.
- ➞ ideally, figure panels should include only measurements that are directly comparable to each other and obtained with the same assay.
- ➞ plots include clearly labeled error bars for independent experiments and sample sizes. Unless justified, error bars should not be shown for technical
- ➞ if n<5, the individual data points from each experiment should be plotted. Any statistical test employed should be justified.
- ➞ Source Data should be included to report the data underlying figures according to the guidelines set out in the authorship guidelines on Data

2. Captions
Each figure caption should contain the following information, for each panel where they are relevant:
- ➞ a specification of the experimental system investigated (eg cell line, species name).
- ➞ the assay(s) and method(s) used to carry out the reported observations and measurements.
- ➞ an explicit mention of the biological and chemical entity(ies) that are being measured.
- ➞ an explicit mention of the biological and chemical entity(ies) that are altered/varied/perturbed in a controlled manner.
- ➞ the exact sample size (n) for each experimental group/condition, given as a number, not a range;
- ➞ a description of the sample collection allowing the reader to understand whether the samples represent technical or biological replicates (including how many animals, litters, cultures, etc.).
- ➞ a statement of how many times the experiment shown was independently replicated in the laboratory.
- ➞ definitions of statistical methods and measures:
  - common tests, such as t-test (please specify whether paired vs. unpaired), simple χ2 tests, Wilcoxon and Mann-Whitney tests, can be unambiguously identified by name only, but more complex techniques should be described in the methods section;
  - are tests one-sided or two-sided?
  - are there adjustments for multiple comparisons?
  - exact statistical test results, e.g., P values = x but not P values < x;
  - definition of 'center values' as median or average;
  - definition of error bars as s.d. or s.e.m.

Please complete ALL of the questions below.
Select "Not Applicable" only when the requested information is not relevant for your study.

Materials

Newly Created Materials	Information included in the manuscript?	In which section is the information available? (Reagents and Tools Table, Materials and Methods, Figures, Data Availability Section)
New materials and reagents need to be available; do any restrictions apply?	Not Applicable	

Antibodies	Information included in the manuscript?	In which section is the information available? (Reagents and Tools Table, Materials and Methods, Figures, Data Availability Section)
For **antibodies** provide the following information:<br>- Commercial antibodies: RRID (if possible) or supplier name, catalogue number and or/clone number<br>- Non-commercial: RRID or citation	Yes	Materials and Methods

DNA and RNA sequences	Information included in the manuscript?	In which section is the information available? (Reagents and Tools Table, Materials and Methods, Figures, Data Availability Section)
**Short novel DNA or RNA including primers, probes**: provide the sequences.	Yes	Materials and Methods

Cell materials	Information included in the manuscript?	In which section is the information available? (Reagents and Tools Table, Materials and Methods, Figures, Data Availability Section)
**Cell lines:** Provide species information, strain. Provide accession number in repository **OR** supplier name, catalog number, clone number, and/**OR** RRID.	Yes	Materials and Methods
**Primary cultures:** Provide species, strain, sex of origin, genetic modification status.	Not Applicable	
Report if the cell lines were recently **authenticated** (e.g., by STR profiling) and tested for mycoplasma contamination.	Yes	Materials and Methods

Experimental animals	Information included in the manuscript?	In which section is the information available? (Reagents and Tools Table, Materials and Methods, Figures, Data Availability Section)
**Laboratory animals or Model organisms:** Provide species, strain, sex, age, genetic modification status. Provide accession number in repository **OR** supplier name, catalog number, clone number, **OR** RRID.	Not Applicable	
**Animal observed in or captured from the field:** Provide species, sex, and age where possible.	Not Applicable	
Please detail **housing and husbandry conditions**.	Not Applicable	

Plants and microbes	Information included in the manuscript?	In which section is the information available? (Reagents and Tools Table, Materials and Methods, Figures, Data Availability Section)
**Plants:** provide species and strain, ecotype and cultivar where relevant, unique accession number if available, and source (including location for collected wild specimens).	Not Applicable	
**Microbes:** provide species and strain, unique accession number if available, and source.	Not Applicable	

Human research participants	Information included in the manuscript?	In which section is the information available? (Reagents and Tools Table, Materials and Methods, Figures, Data Availability Section)
If collected and within the bounds of privacy constraints report on age, sex and gender or ethnicity for all study participants.	Not Applicable	

Core facilities	Information included in the manuscript?	In which section is the information available? (Reagents and Tools Table, Materials and Methods, Figures, Data Availability Section)
If your work benefited from core facilities, was their service mentioned in the acknowledgments section?	Yes	Acknowledgements

Design

Study protocol	Information included in the manuscript?	In which section is the information available? (Reagents and Tools Table, Materials and Methods, Figures, Data Availability Section)
If study protocol has been **pre-registered, provide DOI in the manuscript**. For clinical trials, provide the trial registration number **OR** cite DOI.	Not Applicable	
Report the **clinical trial registration number** (at ClinicalTrials.gov or equivalent), where applicable.	Not Applicable	

Laboratory protocol	Information included in the manuscript?	In which section is the information available? (Reagents and Tools Table, Materials and Methods, Figures, Data Availability Section)
Provide DOI OR other citation details if **external detailed step-by-step protocols** are available.	Not Applicable	

Experimental study design and statistics	Information included in the manuscript?	In which section is the information available? (Reagents and Tools Table, Materials and Methods, Figures, Data Availability Section)
Include a statement about **sample size** estimate even if no statistical methods were used.	Yes	Figure Legends
Were any steps taken to minimize the effects of subjective bias when allocating animals/samples to treatment (e.g. **randomization procedure**)? If yes, have they been described?	Not Applicable	
Include a statement about **blinding** even if no blinding was done.	Yes	Materials and Methods
Describe **inclusion/exclusion criteria** if samples or animals were excluded from the analysis. Were the criteria pre-established?<br><br>If sample or data points were omitted from analysis, report if this was due to attrition or intentional exclusion and provide justification.	Yes	Materials and Methods
For every figure, are **statistical tests** justified as appropriate? Do the data meet the assumptions of the tests (e.g., normal distribution)? Describe any methods used to assess it. Is there an estimate of variation within each group of data? Is the variance similar between the groups that are being statistically compared?	Yes	Materials and Methods

Sample definition and in-laboratory replication	Information included in the manuscript?	In which section is the information available? (Reagents and Tools Table, Materials and Methods, Figures, Data Availability Section)
In the figure legends: state number of times the experiment was **replicated** in laboratory.	Yes	Figure Legends
In the figure legends: define whether data describe **technical or biological replicates**.	Yes	Figure Legends

Ethics

Ethics	Information included in the manuscript?	In which section is the information available? (Reagents and Tools Table, Materials and Methods, Figures, Data Availability Section)
Studies involving **human participants**: State details of **authority granting ethics approval** (IRB or equivalent committee(s), provide reference number for approval.	Not Applicable	
Studies involving **human participants**: Include a statement confirming that **informed consent** was obtained from all subjects and that the experiments conformed to the principles set out in the WMA Declaration of Helsinki and the Department of Health and Human Services Belmont Report.	Not Applicable	
Studies involving **human participants:** For publication of **patient photos**, include a statement confirming that consent to publish was obtained.	Not Applicable	
Studies involving experimental **animals**: State details of **authority granting ethics approval** (IRB or equivalent committee(s), provide reference number for approval. Include a statement of compliance with ethical regulations.	Not Applicable	
Studies involving **specimen and field samples:** State if relevant **permits** obtained, provide details of authority approving study; if none were required, explain why.	Not Applicable	

Dual Use Research of Concern (DURC)	Information included in the manuscript?	In which section is the information available? (Reagents and Tools Table, Materials and Methods, Figures, Data Availability Section)
Could your study fall under dual use research restrictions? Please check biosecurity documents and list of **select agents and toxins** (CDC): https://www.selectagents.gov/sat/list.htm	Not Applicable	
If you used a select agent, is the security level of the lab appropriate and reported in the manuscript?	Not Applicable	
If a study is subject to dual use research of concern regulations, is the name of the **authority granting approval and reference number** for the regulatory approval provided in the manuscript?	Not Applicable	

Reporting

The MDAR framework recommends adoption of discipline-specific guidelines, established and endorsed through community initiatives. Journals have their own policy about requiring specific guidelines and recommendations to complement MDAR.

Adherence to community standards	Information included in the manuscript?	In which section is the information available? (Reagents and Tools Table, Materials and Methods, Figures, Data Availability Section)
State if relevant guidelines or checklists (e.g., **ICMJE, MIBBI, ARRIVE, PRISMA**) have been followed or provided.	Not Applicable	
For **tumor marker prognostic studies,** we recommend that you follow the **REMARK** reporting guidelines (see link list at top right). See author guidelines, under 'Reporting Guidelines'. Please confirm you have followed these guidelines.	Not Applicable	
For **phase II and III randomized controlled trials**, please refer to the **CONSORT** flow diagram (see link list at top right) and submit the CONSORT checklist (see link list at top right) with your submission. See author guidelines, under 'Reporting Guidelines'. Please confirm you have submitted this list.	Not Applicable	

Data Availability

Data availability	Information included in the manuscript?	In which section is the information available? (Reagents and Tools Table, Materials and Methods, Figures, Data Availability Section)
Have **primary datasets** been deposited according to the journal's guidelines (see 'Data Deposition' section) and the respective accession numbers provided in the Data Availability Section?	Not Applicable	
Were **human clinical and genomic datasets** deposited in a public access-controlled repository in accordance to ethical obligations to the patients and to the applicable consent agreement?	Not Applicable	
Are **computational models** that are central and integral to a study available without restrictions in a machine-readable form? Were the relevant accession numbers or links provided?	Not Applicable	
If publicly available data were reused, provide the respective **data citations in the reference list**.	Not Applicable	
